# Synthesis and Antiproliferative Activity of Novel Dehydroabietic Acid-Chalcone Hybrids

**DOI:** 10.3390/molecules27113623

**Published:** 2022-06-05

**Authors:** Sophia Grigoropoulou, Dimitra Manou, Antonia I. Antoniou, Artemis Tsirogianni, Carlo Siciliano, Achilleas D. Theocharis, Constantinos M. Athanassopoulos

**Affiliations:** 1 Synthetic Organic Chemistry Laboratory, Department of Chemistry, University of Patras, GR-26504 Patras, Greece; chem3240@upnet.gr (S.G.); tonadoniou@upatras.gr (A.I.A.); a.tsirogian@upnet.gr (A.T.); 2 Biochemistry, Biochemical Analysis & Matrix Pathobiology Research Group, Laboratory of Biochemistry, Department of Chemistry, University of Patras, GR-26504 Patras, Greece; manou.d@upnet.gr; 3 Department of Pharmacy, Health and Nutritional Sciences, Edificio Polifunzionale, I-87036 Arcavacata di Rende, CS, Italy; carlo.siciliano@unical.it

**Keywords:** dehydroabietic acid, chalcones, hybrids, natural products, breast cancer, 5-FU, MCF-7, MDA-MB-231, Hs578T, anticancer activity

## Abstract

Dehydroabietic Acid (DHA, **1**) derivatives are known for their antiproliferative properties, among others. In the context of this work, DHA was initially modified to two key intermediates bearing a C18 methyl ester, a phenol moiety at C12, and an acetyl or formyl group at C13 position. These derivatives allowed us to synthesize a series of DHA-chalcone hybrids, suitable for structure–activity relationship studies (SARS), following their condensation with a variety of aryl-aldehydes and methyl ketones. The antiproliferative evaluation of the synthesized DHA-chalcone hybrids against three breast cancer cell lines (the estrogen-dependent MCF-7 and the estrogen-independent MDA-MB-231 and Hs578T) showed that eight derivatives (**33, 35, 37, 38, 39, 41, 43, 44**) exhibit low micromolar activity levels (IC_50_ 2.21–11.5 μΜ/MCF-7). For instance, some of them showed better activity compared to the commercial anticancer drug 5-FU against MCF-7 cells (**33, 41, 43, 44**) and against MDA-MB231 (**33** and **41**). Hybrid **38** is a promising lead compound for the treatment of MCF-7 breast cancer, exhibiting comparable activity to 5-FU and being 12.9 times less toxic (SI = 22.7). Thus, our findings suggest that DHA-chalcone hybrids are drug candidates worth pursuing for further development in the search for novel breast cancer therapies.

## 1. Introduction

**Figure 1:**
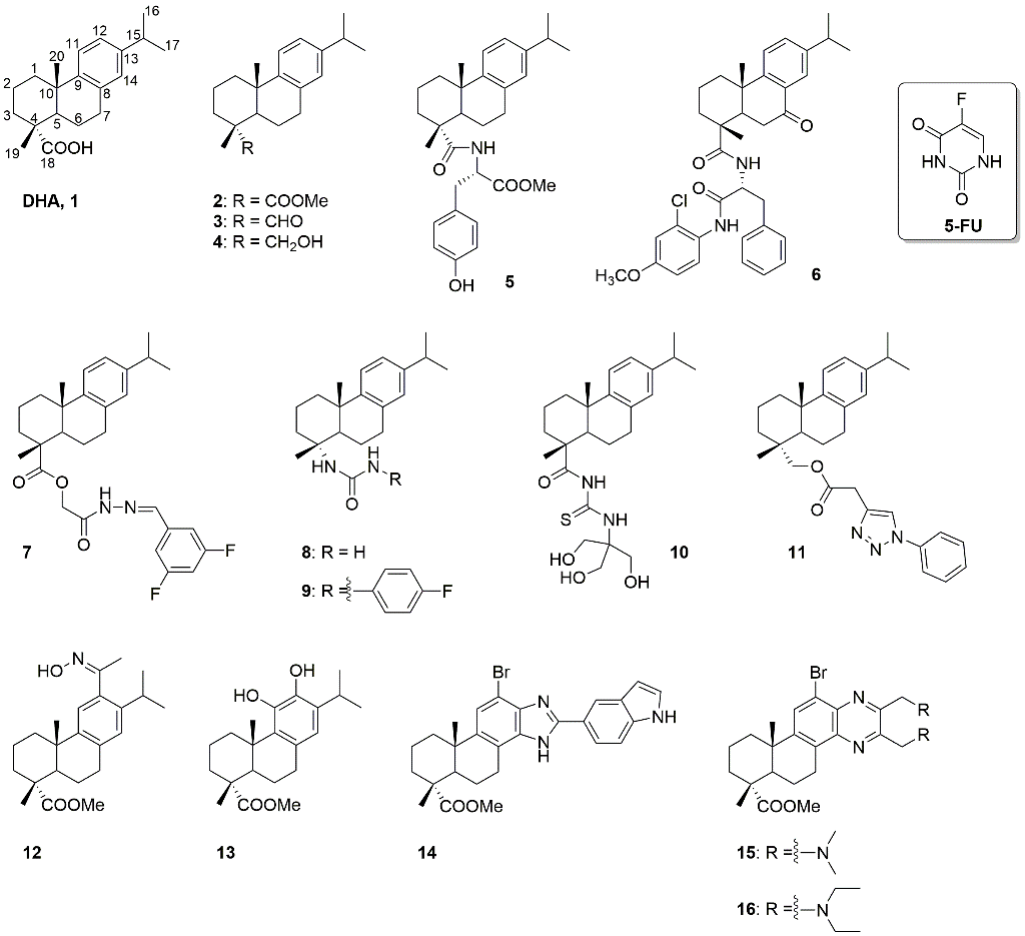
Structures of DHA (**1**) and its analogues (**2**–**16**) with anticancer activity.

Cancer is the second leading cause of deaths worldwide, after cardiovascular diseases [1]. One of the main reasons for the failure of the conventionally used chemotherapeutic agents to treat cancer is the development of resistance and the lack of selectivity against normal cells, which is also accompanied by high cytotoxicity and undesired side effects. Thus, there is a considerable need for the design and development of new, more effective molecules displaying high selectivity for cancer cells. Over the last decades, natural products have played a crucial role in the field of drug discovery, and they became the major source of new anticancer drug candidates [2,3,4,5]. Among them, a special place is taken by dehydroabietic acid (DHA, **1**; Figure 1), a natural diterpenic resin acid, and its derivatives. They have shown a plethora of biological activities, such as antioxidant [6], anti-microbial [7], anti-inflammatory [8], anti-proliferative [9], and anti-cancer [6,10,11,12].

In Figure 1, some representative DHA analogues are depicted, featuring functional and structural modifications in one or more positions of the parent molecule. These compounds are active against several cancer cell lines, with their corresponding IC_50_ values ranging between 0.08 and 83.3 μM. The most studied modifications of the DHA skeleton concern the carboxyl group at C(18) and involve its transformation to esters (**2**, **7**, **12**–**16**) and amides (**5**, **6**), as well as its reduction to the aldehyde **3** or alcohol **4**. Comparing the antitumor effectiveness against HeLa and Jurkat cell lines of **2**, **3** and **4**, the latter showed to be the most active [13]. In another study, DHA amides with natural amino acids were evaluated against HL60, A549, AZS21, and SK-BR-3 cancer cell lines, and the *L*-tyrosine derivative **5** proved to be the most potent with IC_50_ values ranging from 2.3 to 8.1 μM [14]. On the other hand, replacement of the amino acid moiety at C(18) amides with a dipeptide and oxidation of C-7 resulted in a lead compound **6** [15], which exhibited higher cytotoxicity against HeLa, NCl-H460, and MGC-803 human cancer cell lines in the range of 7.7–24.3 μM compared to the positive control 5-fluorouracil (5-FU), a well-established anticancer drug (IC_50_ = 30.4–36.5 μM).

Li et al. reported a series of DHA derivatives functionalized with an acylhydrazone moiety at C(18) [16]. Among them, compound **7** showed the most potent activity against CNE-2, HepG2, HeLa, and BeL-7402 cancer cells, while it exhibited low cytotoxicity against normal HL-7702 human liver cells. It is also worth noting that the urea and acylthiourea derivatives **8**–**9** and **10**, respectively, showed enhanced cytotoxic activity against SMMC7721 cells [17,18]. A series of reduced DHA triazolyl derivatives, suitable for structure–activity relationship studies (SARS), has been prepared by Pertino et al. using click chemistry strategies [19]. Among them, derivative **11** showed the highest activity against three human cancer cell lines, MRC-5, AGS, and SK-MES-1.

Modifications at positions C(11) and C(12) led to other potential anticancer agents, such as the oxime **12** [20], with cytotoxic activity against Aspc-1 pancreatic cancer cells, and catechol **13** [21] against MCF-7 breast cancer cells. Functionalization of the C(13) and C(14) positions has also been reported. Miao et al. prepared a library of 2-aryl-benzimidazole derivatives with compound **14** [22] to be the most potent against SMMC-7721, MDA-MB-231, HeLa, and CT-26 cancer cell lines (IC_50_ = 0.08–0.42 μΜ). Moreover, a series of quinoxaline derivatives was synthesized by Gu et al. [23] and evaluated for cytotoxic activity against three cancer cell lines (MCF-7, SMMC-7721, and HeLa), two of which (**15** and **16**) are considered as lead compounds with IC_50_ values between 0.7 and 2.4 μΜ.

**Figure 2:**
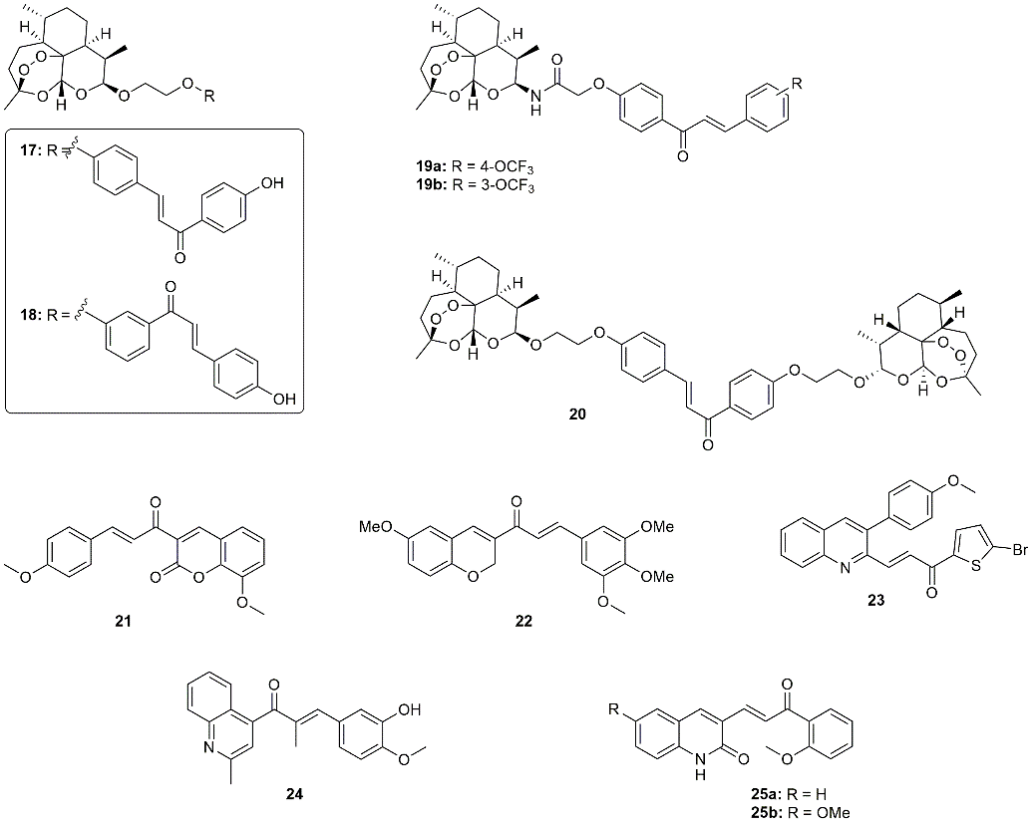
Structures of artemisinin-, coumarin- and quinoline-chalcone hybrids **17**–**25** with anticancer activity.

Main cancer therapy limitations, such as the therapeutical efficacy and the drug resistance, can be dealt with the development of hybrid molecules bearing more than one pharmacophore, thus allowing them to act simultaneously on multiple targets [24]. In this field, the chalcone moiety has proven to be a useful template for the development of novel anticancer agents. Thus, various conjugates and hybrids of natural products bearing a chalcone moiety have been synthesized and evaluated for their anticancer activity. Artemisinin derivatives conjugated with chalcone moieties, e.g., compounds **17**–**19** [25,26] (Figure 2), as well as dimeric artemisinin derivatives containing substituted chalcones as a linker, such as compound **20**, displayed enhanced and selective cytotoxicity against human cancer cell lines, compared to dihydroartemisinin [26]. Moreover, coumarin and quinoline scaffolds have played an important role in anticancer drug development for the treatment of breast, colon, lung, and stomach cancers [27,28]. Thus, hybridization of the above natural products with chalcone provided new potent anticancer candidates, e.g., compounds **21**–**25** [29,30,31,32,33] (Figure 2), possessing bioactivities that have been found to be comparable to those of commercially available anticancer drugs, such as combrestatin [24], topotecan [26], and paclitaxel [28].

In connection to our previous studies where we used dehydroabietic acid as a chiral template to prepare either anticancer [34] or antimicrobial agents [35] and considering the potential of a chalcone pharmacophore, we thought it of interest to synthesize a series of hybrid DHA-chalcone molecules (suitable for structure–activity relationship studies) and to evaluate them for their antiproliferative activity against three breast cancer cell lines (MCF-7, MDA-MB-231, and Hs578T). The synthetic methodology is described below and involves two key intermediates derived from DHA, the DHA C13 ketone **28** and the DHA C13 aldehyde **30**.

## 2. Results and Discussion

### 2.1. Synthesis of the DHA-Chalcone Hybrids ***31***–***53***

**Scheme 1:**
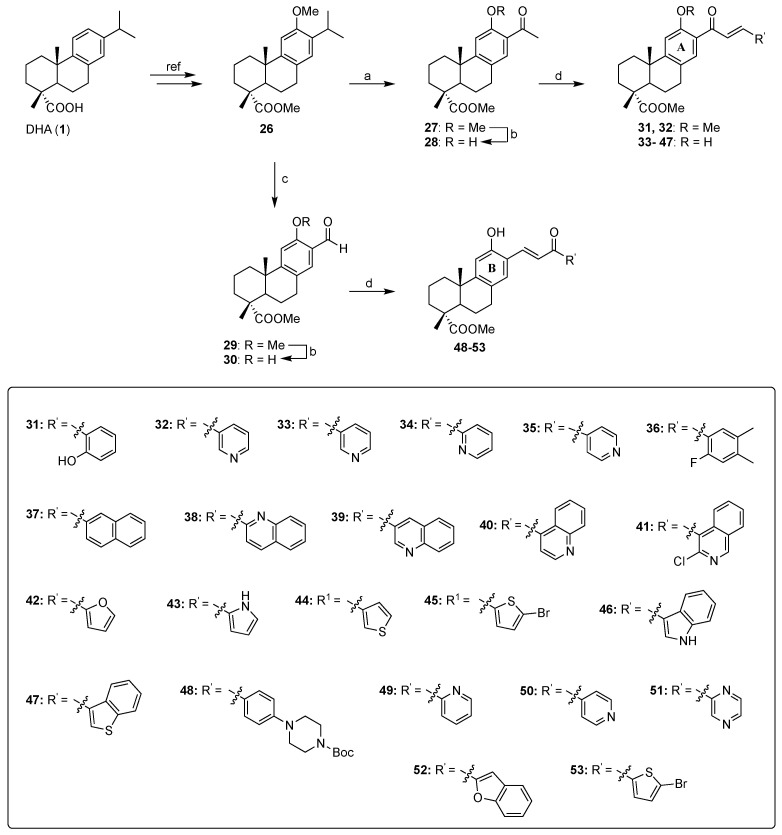
Synthesis of the DHA-chalcone hybrids **31**–**53**; Reagents and conditions: (**a**) CH_3_COCl, AlCl_3_, DCM, 0 °C to rt, 7 h, 93% (**b**) AlCl_3_, DCM, reflux, 2 h, 97%; (**c**) CH_3_OCHCl_2_, AlCl_3_, DCM, −35 °C, 1 h, 85%; (**d**) aryl methyl ketone or arylaldehyde, Ba(OH)_2_ · 8H_2_O, EtOH, rt, 24 h, 62–90%.

The proposed DHA-chalcone hybrids (Scheme 1) were synthesized from the readily available DHA (**1**), which was easily transformed to the C13 acetyl **28** and formyl **30** derivatives the based on experimental procedures described previously [34]. Cross-aldol condensation of **27** or **28** with arylaldehydes in the presence of barium hydroxide octahydrate gave the corresponding hybrid compounds **31**–**47**, where the DHA’s aromatic ring coincides with the chalcone “A” ring [36].

On the other hand, ipso-formylation of **26** by treatment with dichloromethyl methyl ether/AlCl_3_ at −35 °C provided the 12-methoxy aldehyde **29** [37], which upon further treatment with excess AlCl_3_ under reflux in DCM gave the desired 12-hydroxy aldehyde **30**. Condensation of **29** or **30** with suitable aryl methyl ketones mediated by barium hydroxide octahydrate afforded the corresponding hybrid compounds **48**–**53**, where the DHA’s aromatic ring coincides with the chalcone “B” ring.

### 2.2. Biological Evaluation

The antiproliferative activity of the synthesized DHA-chalcone hybrids **31**–**53** was evaluated using three characteristic breast carcinoma cell lines, MDA-MB-231, MCF-7, and Hs578T. For the sake of comparison, 5-FU, a widely used drug in clinical practice, was selected as positive control.

Regarding the different characteristics of the used breast cancer cell lines, we can notice that MDA-MB-231 and Hs578T are triple negative. Nevertheless, MDA-MB-231 is (ERβ)-positive and both cell lines exhibit high metastatic and aggressive potential, whereas MCF-7 cells are estrogen receptor alpha (Erα)-positive and exhibit low metastatic potential and low aggressiveness. The estimated IC_50_ values of these compounds are provided in Table 1.

**Figure 3:**
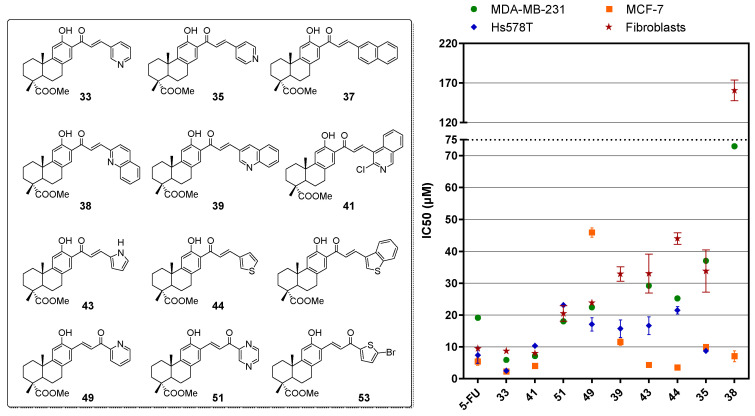
Structures of the most potent compounds and their IC_50_ values against all tested cell lines including the positive control (5-FU).

In order to calculate the selectivity index of the most potent compounds (**33**, **35**, **38**, **39**, **41**, **43**, **44**, **49**, and **51**), their cytotoxicity was evaluated against normal primary fibroblasts (FB) and the corresponding results (FB-IC_50_ and Sis) are presented again in Table 1 and Figure 3.

A general comment regarding the activity of the hybrid chalcones is that the presence of a phenol at the C12 position of DHA, as well as the incorporation of the aromatic “A” ring of the chalcone to the skeleton of DHA (compounds **31**–**47**), represent two essential structural elements for the potency of the hybrids. Moreover, we notice an enhanced cytotoxicity of our compounds against MCF-7 cells, in comparison to the other two cancer cell lines, MDA-MB231 and Hs578T.

According to Figure 3 and Table 1, is evident that compound **33** is the most potent hybrid for all three cancer cell lines and shows a slightly better activity, in comparison to the control drug 5-FU (2.4–3.3 times more active). Regarding its selectivity indices (SIs), over the three tested breast cancer lines, compound **33** seems to be 2.2–3 times less toxic than 5-FU. 

**Figure 4:**
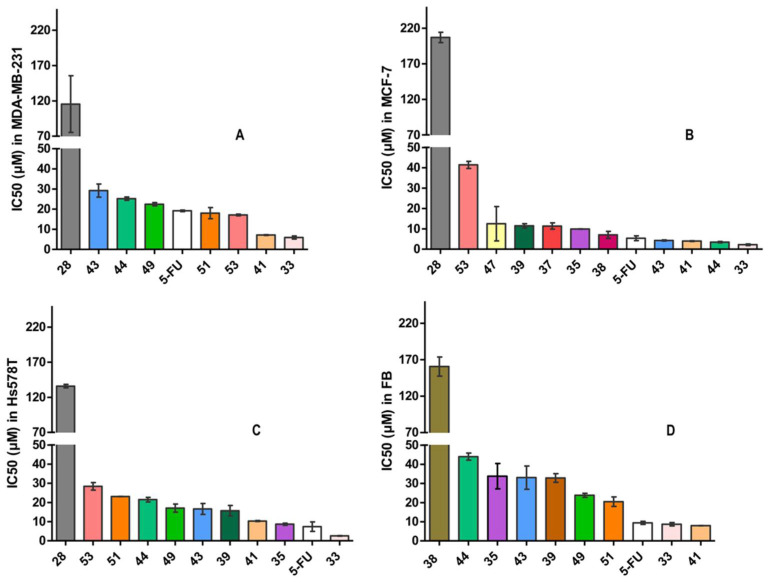
IC_50_ values of the most potent compounds and 5-FU (positive control) against each cancer cell line; (**A**) MDA-MB-231; (**B**) MCF-7; (**C**) Hs578T; and (**D**) against normal primary FB.

The IC_50_ values presented in Figure 4A suggest that the compounds **51** and **53** exhibit a comparable 5-FU activity against MDA-MB-231 cells, whereas compounds **33** and **41** a better one. Regarding the MCF-7 evaluation results (Figure 4B), we notice that compound **33,** together with compounds **41**, **43,** and **44**, showed higher activity than 5-FU. 

Accordingly, the bar graph for the Hs578T cell line shows that 5-FU is more effective than all the synthesized compounds with the exception of **33** (Figure 4C).

Finally, in terms of selectivity, it is apparent that the less toxic compounds are **35**, **38**, **39**, **43**, **44**, **49**, and **51** (Figure 4D). It is worth mentioning that compound **38** (IC_50_ = 7.06 μM) combines a comparable to 5-FU (IC_50_ = 5.37 μM) activity for MCF-7 with a very low toxicity (IC_50_ = 160.65 μΜ, SI = 22.75), being 12.9 times less toxic than 5-FU against normal fibroblasts.

### 2.3. Structure-Activity Relationships

Structurally, the hybrid chalcones can be classified in two series, Series A, where the DHA’s aromatic ring coincides with the chalcone “A” ring (compounds **31**–**47**), and Series B, where the DHA’s aromatic ring coincides with chalcone “B” ring (compounds **48**–**53**).

Regarding the structural elements of the Series A hybrids, which affect their activity, we notice the following:

The introduction of a heteroatom into the aromatic system of ring B remarkably improved their cytotoxicity. More specifically, the pyrrole (**43**) and thiophene (**44**) derivatives are highly cytotoxic against the MCF-7 cancer cell line. However, a fused heterocyclic form for ring B, such as an indole (**46**) or benzothiophene (**47**), as well as the presence of a bromo substituent on the thiophene heterocycle of **45**, led to less active compounds or to complete loss of activity.

Among pyridyl type derivatives **32**–**35**, the 3-pyridyl derivative **33** showed to be the most active compound against all the cancer cell lines (IC_50_ = 2.21–5.89 μM) and had similar cytotoxicity with 5-FU (Figure 3, Table 1).

On the other hand, the quinoline derivatives **38**–**41** showed moderate activity, except hybrid **41**, which was active against all cancer cell lines, with IC_50_ = 7.12 μΜ (ΜDA-MB-231), 3.99 μΜ (MCF-7), and 10.31 μΜ (Hs578T), however it showed high toxicity against normal fibroblasts (IC_50_ = 7.96 μΜ).

Regarding Series B hybrids (**48**–**53**), we observe that all of them presented low to moderate activity (over 17 μΜ).

## 3. Materials and Methods

### 3.1. General Methods

^1^H NMR spectra were obtained at 600.13 MHz and ^13^C NMR spectra at 150.90 MHz on a Bruker AVANCEIII HD spectrometer. Chemical shifts (*δ*) are indicated in parts per million (ppm) downfield from TMS and coupling constants (*J*) are reported in hertz. ESI mass spectra were recorded at 30 V, on a Micromass-Platform LC spectrometer using MeOH as solvent. HR mass spectra were performed using an ESI-LTQ-ORBITRAP XL unit (Thermo Scientific, Bremen, Germany). The Orbitrap Unit was operated in positive mode, with a spay voltage of 3.2 kV, while the sheath gas flow rate and auxiliary gas flow rate were adjusted to 12 and 2 arbitrary units, respectively. The capillary voltage and the tube lens voltage were set to 10 and 110 V, respectively. The scan ranged from m/z 150 up to 2000. All solvents were dried and/or purified according to standard procedures prior to use. Anhydrous Na_2_SO_4_ was used for drying solutions, and the solvents were then routinely removed at ca. 40 °C under reduced pressure using a rotary vacuum evaporator. All reagents employed in the present work were commercially available and used without further purification. When required, reactions were carried out under dry argon atmosphere in preflamed glassware. Flash column chromatography (FCC) was performed on Merck silica gel 60 (230–400 mesh) and analytical thin layer chromatography (TLC) was performed on Merck silica gel 60F_254_ (0.2 mm) precoated on aluminum foil. Spots on the TLC plates were visualized with UV light at 254 nm using ninhydrin solution or para-anisaldehyde solution (Supplementary Material). 

### 3.2. Experimental Procedures

#### General Procedure for the Synthesis of the DHA-Chalcone Hybrids

To a stirred solution of **27** or **28** or **29** or **30** (1 eq) and carboxaldehyde or aromatic methyl ketone (1 eq) in absolute EtOH was added portion of Ba(OH)_2_·8H_2_O (1 eq) every 10 min. The reaction mixture was stirred at room temperature for 24–48 h and the progress of the reaction was monitored by TLC. In case of non-completion of the reaction, a 10% excess of Ba(OH)_2_·8H_2_O was added. After completion of the reaction, the mixture was diluted with AcOEt and the organic layer was washed sequentially with cold 5% aqueous citric acid, H_2_O, and brine. After being dried over anhydrous Na_2_SO_4_, the organic extracts were filtered and evaporated to dryness under reduced pressure. The residue was subjected to FCC, using system PhMe/AcOEt, to give the pure product as yellow oil in a yield range of 62–90%.

(1R,4aS)-methyl 7-((E)-3-(2-hydroxyphenyl)-3-oxoprop-1-en-1-yl)-6-methoxy-1,4a-dimethyl-1,2,3,4,4a,9,10,10a-octahydrophenanthrene-1-carboxylate (**31**): 84%; MS (ESI, 30eV): *m/z* 471.21 [M + Na], 449.25 [M + H]; ^1^H NMR (CDCl_3_) δ 13.00 (s, 1H), 8.16 (d, *J* = 15.5 Hz, 1H), 7.93 (dd, *J* = 8.1, 1.7 Hz, 1H), 7.74 (d, *J* = 15.6 Hz, 1H), 7.49–7.45 (m, 1H), 7.01 (dd, *J* = 8.3, 1.2 Hz, 1H), 6.95–6.91 (m, 1H), 6.82 (s, 1H), 3.91 (s, 3H), 3.69 (s, 3H), 2.91–2.85 (m, 2H), 2.30 (d, *J* = 11.7 Hz, 1H), 2.23 (dd, *J* = 12.5, 2.2 Hz, 1H), 1.88–1.72 (m, 5H), 1.70–1.65 (m, 1H), 1.48–1.43 (m, 1H), 1.30 (s, 3H), 1.25 (s, 3H); ^13^C NMR (CDCl_3_) δ 194.5, 179.0, 163.7, 157.6, 154.4, 141.4, 136.1, 130.5, 129.8, 127.6, 121.5, 120.4, 120.1, 118.8, 118.6, 107.2, 55.8, 52.2, 47.8, 44.7, 38.1, 36.7, 29.1, 24.9, 21.7, 18.7, 16.7.

(1R,4aS)-methyl 6-methoxy-1,4a-dimethyl-7-((E)-3-(pyridin-3-yl)acryloyl)-1,2,3,4,4a, 9,10,10a-octahydrophenanthrene-1-carboxylate (**32**): 76%; MS (ESI, 30eV): *m/z* 456.12 [M + Na], 434.09 [M + H]; ^1^H NMR (CDCl_3_) δ 8.81 (s, 1H), 8.60–8.57 (m, 1H), 7.89–7.86 (m, 1H), 7.62 (d, *J* = 15.9 Hz, 1H), 7.52 (d, *J* = 15.9 Hz, 1H), 7.38 (s, 1H), 7.34–7.31 (m, 1H), 6.86 (s, 1H), 3.88 (s, 3H), 3.68 (s, 3H), 2.89–2.84 (m, 2H), 2.31 (d, *J* = 11.5 Hz, 1H), 2.22 (dd, *J* = 12.5, 2.3 Hz, 1H), 1.83–1.73 (m, 5H), 1.47–1.41 (m, 2H), 1.29 (s, 3H), 1.25 (s, 3H); ^13^C NMR (CDCl_3_) δ 191.3, 178.8, 156.8, 155.5, 150.5, 149.7, 138.2, 134.7, 131.4, 131.2, 129.0, 127.8, 126.4, 123.7, 107.6, 55.8, 52.0, 47.6, 44.5, 38.0, 37.9, 36.5, 28.8, 24.8, 21.5, 18.5, 16.6.

(1R,4aS)-methyl 6-hydroxy-1,4a-dimethyl-7-((E)-3-(pyridin-3-yl)acryloyl)-1,2,3,4,4a, 9,10,10a-octahydrophenanthrene-1-carboxylate (**33**): 74%; MS (ESI, 30eV): *m/z* 442.21 [M + Na]; ^1^H NMR (CDCl_3_) δ 12.30 (s, 1H), 8.90 (s, 1H), 8.65 (d, *J* = 4.9 Hz, 1H), 8.01 (dt, *J* = 7.9, 4.0 Hz, 1H), 7.86 (d, *J* = 15.6 Hz, 1H), 7.69 (d, *J* = 15.6 Hz, 1H), 7.55 (s, 1H), 7.42 (dd, *J* = 8.0, 4.7 Hz, 1H), 6.92 (s, 1H), 3.68 (s, 3H), 2.95–2.86 (m, 2H), 2.28 (d, *J* = 12.8 Hz, 1H), 2.21 (dd, *J* = 12.5, 2.4 Hz, 1H), 1.88–1.64 (m, 5H), 1.56–1.43 (m, 2H), 1.29 (s, 3H), 1.23 (s, 3H); ^13^C NMR (CDCl_3_) δ 192.5, 179.0, 161.7, 160.0, 150.8, 149.6, 140.8, 135.5, 131.0, 130.0, 126.2, 124.1, 122.8, 118.1, 114.0, 52.2, 47.8, 38.2, 37.7, 36.7, 29.2, 24.7, 21.7, 18.6, 16.8; HR-ESI: *m/z* 420.2164; [M + H^+^] for the compound C_26_H_29_NO_4_ requires 420.2169.

(E)-1-((4bS,8R)-3-hydroxy-8-methoxy-4b,8-dimethyl-4b,5,6,7,8,8a,9,10-octahydrophenanthren-2-yl)-3-(pyridin-2-yl)prop-2-en-1-one (**34**): 86%; MS (ESI, 30eV): *m/z* 442.39 [M + Na], 420.46 [M + H]; ^1^H NMR (CDCl_3_) δ 12.31 (s, 1H), 8.76 (d, *J* = 5.1 Hz, 1H), 8.47 (br s, 1H), 7.91 (s, 1H), 7.83 (s, 1H), 7.80 (d, *J* = 15.2 Hz, 1H), 7.58 (d, *J* = 7.7 Hz, 1H), 7.44 (s, 1H), 6.91 (s, 1H), 3.68 (s, 3H), 2.99–2.85 (m, 2H), 2.35 (t, *J* = 7.6 Hz, 1H), 2.27 (d, *J* = 11.6 Hz, 1H), 2.21 (dd, *J* = 12.5, 2.5 Hz, 1H), 1.87–1.64 (m, 5H), 1.48–1.43 (m, 1H), 1.29 (s, 3H), 1.22 (s, 3H); ^13^C NMR (CDCl_3_) δ 192.9, 178.8, 161.5, 159.9, 153.4, 151.0, 130.8, 126.5, 126.3, 124.9, 118.2, 113.5, 52.0, 47.6, 44.2, 38.1, 37.6, 36.5, 28.9, 24.5, 21.5, 18.4, 16.6.

(1R,4aS)-methyl 6-hydroxy-1,4a-dimethyl-7-((E)-3-(pyridin-4-yl)acryloyl)-1,2,3,4,4a, 9,10,10a-octahydrophenanthrene-1-carboxylate (**35**): 85%; MS (ESI, 30eV): *m/z* 442.34 [M + Na], 420.18 [M + H]; ^1^H NMR (CDCl_3_) δ 12.22 (s, 1H), 8.71 (d, *J* = 6.1 Hz, 2H), 7.76 (d, *J* = 3.8 Hz, 2H), 7.53 (s, 1H), 7.50 (d, *J* = 6.1 Hz, 2H), 6.93 (s, 1H), 3.69 (s, 3H), 2.93–2.87 (m, 2H), 2.28 (d, *J* = 12.8 Hz, 1H), 2.21 (dd, *J* = 12.5, 2.6 Hz, 1H), 1.88–1.64 (m, 5H), 1.54–1.43 (m, 2H), 1.29 (s, 3H), 1.23 (s, 3H); ^13^C NMR (CDCl_3_) δ 178.6, 177.2, 160.5, 157.8, 154.7, 150.6, 133.9, 129.0, 128.2, 125.7, 122.4, 120.3, 113.2, 52.1, 47.5, 44.1, 38.2, 38.0, 36.5, 31.1, 29.1, 25.0, 21.2, 18.4, 16.6.

(1R,4aS)-methyl 6-hydroxy-1,4a-dimethyl-7-((E)-3-(2,4,5-trifluorophenyl)acryloyl)-1,2,3,4,4a,9,10,10a-octahydrophenanthrene-1-carboxylate (**36**): 89%; MS (ESI, 30eV): *m/z* 495.23 [M + Na], 473.44 [M + H]; ^1^H NMR (CDCl_3_) δ 12.30 (s, 1H), 7.86 (d, *J* = 15.7 Hz, 1H), 7.62 (d, *J* = 15.7 Hz, 1H), 7.53–7.46 (m, 2H), 7.06–7.00 (m, 1H), 6.92 (s, 1H), 3.69 (s, 3H), 2.94–2.87 (m, 2H), 2.27 (d, *J* = 12.8 Hz, 1H), 2.21 (dd, *J* = 12.5, 2.4 Hz, 1H), 1.88–1.73 (m, 4H), 1.69–1.66 (m, 1H), 1.55–1.45 (m, 2H), 1.29 (s, 3H), 1.23 (s, 3H); ^13^C NMR (CDCl_3_) δ 192.6, 179.0, 161.7, 160.0, 135.4, 130.0, 128.1, 126.2, 123.7, 118.2, 113.9, 106.5, 52.2, 47.8, 44.3, 38.2, 37.7, 36.7, 29.2, 24.7, 21.7, 18.6, 16.8.

(1R,4aS)-6-hydroxy-1,4a-dimethyl-7-((E)-3-(naphthalen-2-yl)acryloyl)-1,2,3,4,4a,9,10, 10a-octahydrophenanthrene-1-carboxylic acid (**37**): 76%; MS (ESI, 30eV): *m/z* 507.33 [M + K], 469.36 [M + H]; ^1^H NMR (CDCl_3_) δ 12.52 (s, 1H), 8.06 (d, *J* = 12.8 Hz, 1H), 7.92–7.81 (m, 5H), 7.73–7.32 (d, *J* = 15.5 Hz, 1H), 7.62 (s, 1H), 7.59–7.52 (m, 2H), 6.93 (s, 1H), 3.69 (s, 3H), 2.96–2.90 (m, 2H), 2.29 (d, *J* = 11.6 Hz, 1H), 2.23 (dd, *J* = 12.5, 2.3 Hz, 1H), 1.90–1.65 (m, 5H), 1.53–1.45 (m, 2H), 1.30 (s, 3H), 1.24 (s, 3H); ^13^C NMR (CDCl_3_) δ 193.2, 179.0, 161.6, 159.5, 145.2, 134.6, 133.5, 132.4, 131.1, 130.0, 129.0, 128.9, 128.0, 127.7, 127.00, 126.0, 123.9, 120.6, 118.5, 113.9, 52.2, 47.8, 44.4, 38.2, 37.8, 36.7, 29.2, 24.7, 21.8, 18.6, 16.8.

(1R,4aS)-methyl 6-hydroxy-1,4a-dimethyl-7-((E)-3-(quinolin-2-yl)acryloyl)-1,2,3,4,4a, 9,10,10a-octahydrophenanthrene-1-carboxylate (**38**): 71%; MS (ESI, 30eV): *m/z* 492.11 [M + Na], 470.38 [M + H]; ^1^H NMR (CDCl_3_) δ 12.30 (s, 1H), 8.99 (d, *J* = 4.4 Hz, 1H), 8.60 (d, *J* = 15.4 Hz, 1H), 8.21 (d, *J* = 14.3 Hz, 1H), 8.18 (d, *J* = 9.7 Hz, 1H), 7.81–7.76 (m, 2H), 7.69–7.64 (m, 2H), 7.57 (s, 1H), 6.95 (s, 1H), 3.68 (s, 3H), 2.95–2.87 (m, 2H), 2.30–2.26 (m, 1H), 2.21 (dd, *J* = 12.5, 2.6 Hz, 1H), 1.88–1.66 (m, 5H), 1.60–1.51 (m, 1H), 1.50–1.45 (m, 1H), 1.29 (s, 3H), 1.24 (s, 3H); ^13^C NMR (CDCl_3_) δ 193.4, 179.0, 161.5, 159.4, 138.4, 138.2, 129.9, 129.8, 129.2, 128.4, 127.3, 125.9, 125.5, 120.2, 118.4, 113.8, 52.2, 47.8, 44.4, 38.2, 37.8, 36.7, 29.2, 24.7, 21.8, 18.6, 16.8; HR-ESI: *m/z* 470.2305; [M + H^+^] for the compound C_30_H_31_NO_4_ requires 470.2326.

(1R,4aS)-methyl 6-hydroxy-1,4a-dimethyl-7-((E)-3-(quinolin-3-yl)acryloyl)-1,2,3,4,4a, 9,10,10a-octahydrophenanthrene-1-carboxylate (**39**): 62%; MS (ESI, 30eV): *m/z* 492.29 [M + Na], 470.29 [M + H]; ^1^H NMR (CDCl_3_) δ 12.33 (s, 1H), 8.48 (s, 1H), 8.33 (s, 1H), 8.29–8.14 (m, 1H), 8.03 (d, *J* = 15.7 Hz, 1H), 7.96 (d, *J* = 8.7 Hz, 1H), 7.91 (d, *J* = 6.8 Hz, 1H), 7.85–7.81 (m, 1H), 7.70–7.64 (m, 1H), 7.62 (s, 1H), 6.94 (s, 1H), 3.69 (s, 3H), 2.63–2.58 (m, 2H), 2.98–2.90 (m, 2H), 2.37–2.32 (m, 2H), 2.29 (d, *J* = 12.4 Hz, 1H), 2.23 (dd, *J* = 12.5, 2.4 Hz, 1H), 1.70–1.47 (m, 3H), 1.30 (s, 3H), 1.24 (s, 3H); ^13^C NMR (CDCl_3_) δ 194.8, 178.8, 161.6, 159.9, 150.0, 148.2, 131.8, 131.3, 129.9, 129.8, 129.2, 127.9, 127.8, 126.2, 124.5, 120.8, 118.0, 113.8, 52.0, 47.6, 44.2, 38.1, 37.6, 36.6, 29.6, 24.5, 21.6, 18.4, 16.6.

(1R,4aS)-methyl 6-hydroxy-1,4a-dimethyl-7-((E)-3-(quinolin-4-yl)acryloyl)-1,2,3,4,4a, 9,10,10a-octahydrophenanthrene-1-carboxylate (**40**): 68%; MS (ESI, 30eV): *m/z* 508.37 [M + K], 497.47 [M + Na], 470.25 [M + H]; ^1^H NMR (CDCl_3_) δ 12.28 (s, 1H), 8.60 (d, *J* = 15.4 Hz, 1H),8.24–8.18 (m, 2H), 7.82–7.75 (m, 2H), 7.70–7.63 (m, 3H), 7.57 (s, 1H), 6.95 (s, 1H), 3.68 (s, 3H), 2.94–2.86 (m, 2H), 2.29 (d, *J* = 12.6 Hz, 1H), 2.22 (dd, *J* = 12.6, 2.4 Hz, 1H), 1.94–1.63 (m, 5H), 1.56–1.40 (m, 2H), 1.29 (s, 3H), 1.24 (s, 3H); ^13^C NMR (CDCl_3_) δ 192.4, 179.0, 161.8, 160.3, 159.4, 150.1, 140.8, 139.2, 131.2, 130.2, 127.7, 126.9, 126.4, 126.0, 123.6, 118.4, 114.0, 113.9, 52.2, 47.8, 44.3, 38.3, 37.7, 36.7, 29.1, 24.7, 21.7, 18.6, 16.8; HR-ESI: *m/z* 470.2304; [M + H^+^] for the compound C_30_H_31_NO_4_ requires 470.2326.

(1R,4aS)-methyl 7-((E)-3-(2-chloroquinolin-4-yl)acryloyl)-6-hydroxy-1,4a-dimethyl-1,2,3,4, 4a,9,10,10a-octahydrophenanthrene-1-carboxylate (**41**): 67%; MS (ESI, 30eV): *m/z* 526.40 [M + Na], 504.23 [M + H]; ^1^H NMR (CDCl_3_) δ 12.30 (s, 1H), 8.52 (s, 1H), 8.29 (d, *J* = 15.5 Hz, 1H), 8.05 (d, *J* = 8.5 Hz, 1H), 7.93 (d, *J* = 8.0 Hz, 1H), 7.82–7.78 (m, 1H), 7.71 (d, *J* = 15.5 Hz, 1H), 7.64–7.61 (m, 1H), 7.59 (s, 1H), 6.95 (s, 1H), 3.69 (s, 3H), 2.95–2.91 (m, 2H), 2.29 (d, *J* = 12.6 Hz, 1H), 2.22 (dd, *J* = 12.6, 2.4 Hz, 1H), 1.90–1.67 (m, 6H), 1.51–1.49 (m, 1H), 1.60 (s, 3H), 1.24 (s, 3H); ^13^C NMR (CDCl_3_) δ 192.4, 179.0, 161.8, 160.0, 157.4, 148.2, 139.6, 136.6, 131.9, 130.1, 128.7, 128.2, 127.9, 127.2, 126.2, 125.0, 118.0, 114.0, 52.2, 47.8, 44.3, 38.3, 37.7, 36.7, 29.2, 24.7, 21.8, 18.6, 16.8; HR-ESI: *m/z* 504.1908; [M + H^+^] for the compound C_25_H_29_NO_4_ requires 504.1936.

(E)-3-(furan-2-yl)-1-((4bS,8R)-3-hydroxy-8-methoxy-4b,8-dimethyl-4b,5,6,7,8,8a,9,10-octahydrophenanthren-2-yl)prop-2-en-1-one (**42**): 90%; MS (ESI, 30eV): *m/z* 431.36 [M + Na]; ^1^H NMR (CDCl_3_) δ 12.53 (s, 1H), 7.65 (d, *J* = 15.2 Hz, 1H), 7.56 (s, 2H), 7.51 (d, *J* = 15.2 Hz, 1H), 6.90 (s, 1H), 6.75 (d, *J* = 3.5 Hz, 1H), 6.55–6.52 (m, 1H), 3.68 (s, 3H), 2.92–2.88 (m, 2H), 2.27 (d, *J* = 11.1 Hz, 1H), 2.20 (dd, *J* = 12.5, 2.5 Hz, 1H), 1.88–1.61 (m, 6H), 1.55–1.44 (m, 1H), 1.29 (s, 3H), 1.23 (s, 3H); ^13^C NMR (CDCl_3_) δ 192.8, 179.0, 161.5, 159.4, 151.8, 145.4, 130.8, 130.0, 125.9, 118.4, 118.0, 117.0, 113.7, 113.0, 52.2, 47.8, 44.4, 38.2, 37.8, 36.7, 29.2, 24.7, 21.8, 18.6, 16.8.

(1R,4aS)-methyl 7-((E)-3-(1H-pyrrol-3-yl)acryloyl)-6-hydroxy-1,4a-dimethyl-1,2,3,4, 4a,9,10,10a-octahydrophenanthrene-1-carboxylate (**43**): 72%; MS (ESI, 30eV): *m/z* 446.24 [M + K], 430.19 [M + Na], 408.23 [M + H]; ^1^H NMR (CDCl_3_) *δ* 12.71 (s, 1H), 8.76 (s, 1H), 7.79 (d, *J* = 15.4 Hz, 1H), 7.50 (s, 1H), 7.03–6.99 (m, 1H), 6.89 (s, 1H), 6.77–6.74 (m, 1H), 3.69 (s, 3H), 2.91–2.86 (m, 2H), 2.26 (d, *J* = 11.5 Hz, 1H), 2.21 (dd, *J* = 12.4, 2.3 Hz, 1H), 1.88–1.63 (m, 5H), 1.50–1.43 (m, 2H), 1.29 (s, 3H), 1.22 (s, 3H); ^13^C NMR (CDCl_3_) *δ* 192.6, 179.0, 161.4, 158.8, 138.0, 134.4, 129.2, 128.4, 125.5, 123.6, 118.5, 116.2, 113.8, 111.9, 52.2, 47.8, 44.4, 38.1, 37.8, 36.7, 29.2, 24.7, 21.8, 18.6, 16.8; HR-ESI: *m/z* 408.2152; [M + H^+^] for the compound C_25_H_29_NO_4_ requires 408.2169.

(1R,4aS)-methyl 6-hydroxy-1,4a-dimethyl-7-((E)-3-(thiophen-3-yl)acryloyl)-1,2,3,4, 4a,9,10,10a-octahydrophenanthrene-1-carboxylate (**44**): 83%; MS (ESI, 30eV): *m/z* 463.40 [M + K], 447.09 [M + Na], 425.38 [M + H]; ^1^H NMR (CDCl_3_) δ 12.49 (s, 1H), 7.88 (d, *J* = 15.4 Hz, 1H), 7.66–7.63 (m, 1H), 7.53 (s, 1H), 7.47–7.45 (m, 1H), 7.43 (d, *J* = 15.4 Hz, 1H), 7.40–7.38 (m, 1H), 6.91 (s, 1H), 3.68 (s, 3H), 2.94–2.87 (m, 2H), 2.28 (d, *J* = 11.9 Hz, 1H), 2.21 (dd, *J* = 12.5, 2.4 Hz, 1H), 1.89–1.65 (m, 5H), 1.55–1.45 (m, 2H), 1.29 (s, 3H), 1.23 (s, 3H); ^13^C NMR (CDCl_3_) δ 193.2, 178.9, 161.4, 159.2, 138.3, 138.1, 129.8, 129.6, 127.1, 125.7, 125.3, 120.0, 118.2, 113.7, 52.0, 47.6, 44.2, 38.0, 37.6, 36.6, 29.0, 24.5, 21.6, 18.4, 16.6; HR-ESI: *m/z* 425.1764; [M + H^+^] for the compound C_25_H_28_SO_4_ requires 425.1781.

(1R,4aS)-methyl 7-((E)-3-(5-bromothiophen-2-yl)acryloyl)-6-hydroxy-1,4a-dimethyl-1,2,3,4,4a,9,10,10a-octahydrophenanthrene-1-carboxylate (**45**): 67%; MS (ESI, 30eV): *m/z* 541.47 [M + K], 525.40 [M + Na], 503.33 [M + H]; ^1^H NMR (CDCl_3_) δ 12.41 (s, 1H), 7.87 (d, *J* = 15.2 Hz, 1H), 7.47 (s, 1H), 7.28 (d, *J* = 15.2 Hz, 1H), 7.13 (d, *J* = 3.9 Hz, 1H), 7.08 (d, J = 3.9 Hz, 1H), 6.90 (s, 1H), 3.69 (s, 3H), 2.93–2.86 (m, 2H), 2.27 (d, *J* = 11.3 Hz, 1H), 2.21 (dd, *J* = 12.5, 2.4 Hz, 1H), 1.88–1.71 (m, 4H), 1.69–1.64 (m, 1H), 1.54–1.45 (m, 2H), 1.29 (s, 3H), 1.22 (s, 3H); ^13^C NMR (CDCl_3_) δ 192.1, 178.9, 161.4, 159.4, 141.8, 136.3, 123.6, 131.4, 129.6, 125.9, 119.4, 118.0, 116.9, 113.7, 52.0, 47.6, 44.2, 38.0, 37.6, 36.5, 29.0, 24.5, 21.6, 18.4, 16.6.

(1R,4aS)-methyl 7-((E)-3-(1H-indol-3-yl)acryloyl)-6-hydroxy-1,4a-dimethyl-1,2,3,4, 4a,9,10,10a-octahydrophenanthrene-1-carboxylate (**46**): 62%; MS (ESI, 30eV): *m/z* 496.12 [M + K], 480.18 [M + Na], 458.26 [M + H]; ^1^H NMR (CDCl_3_) δ 12.87 (s, 1H), 8.82 (s, 1H), 8.17 (d, *J* = 15.3 Hz, 1H), 8.04–7.99 (m, 1H), 7.66–7.58 (s, 3H), 7.47–7.44 (m, 1H), 7.34–7.31 (m,2H), 6.92 (s, 1H), 3.70 (s, 3H), 2.98–2.88 (m, 2H), 2.28 (d, *J* = 11.8 Hz, 1H), 2.23 (dd, *J* = 12.6, 2.5 Hz, 1H), 1.90–1.65 (m, 5H), 1.57–1.45 (m, 2H), 1.31 (s, 3H), 1.24 (s, 3H); ^13^C NMR (CDCl_3_) δ 193.3, 179.0, 161.3, 158.5, 139.0, 137.3, 130.6, 129.7, 129.0, 128.2, 123.7, 121.9, 120.6, 118.5, 115.8, 114.6, 113.5, 112.1, 52.0, 47.7, 44.3, 37.9, 37.7, 36.6, 29.1, 24.5, 21.6, 18.5, 16.6.

(1R,4aS)-methyl 7-((E)-3-(benzo[b]thiophen-3-yl)acryloyl)-6-hydroxy-1,4a-dimethyl-1,2,3,4,4a,9,10,10a-octahydrophenanthrene-1-carboxylate (**47**): 72%; MS (ESI, 30eV): *m/z* 497.26 [M + Na], 475.48 [M + H]; ^1^H NMR (CDCl_3_) δ 12.81 (s, 1H), 8.58 (s, 1H), 8.18 (d, *J* = 15.4 Hz, 1H), 8.04–7.99 (m, 1H), 7.67–7.65 (m, 1H), 7.62 (d, *J* = 19.3 Hz, 1H), 7.48–7.45 (m, 1H), 7.36–7.32 (m, 2H), 6.91 (s, 1H), 3.69 (s, 3H), 2.98–2.92 (m, 2H), 2.29 (d, *J* = 11.6 Hz, 1H), 2.24 (dd, *J* = 12.5, 2.5 Hz, 1H), 1.91–1.82 (m, 1H), 1.79–1.72 (m, 3H), 1.68–1.66 (m, 1H), 1.57–1.51 (m, 2H), 1.30 (s, 3H), 1.24 (s, 3H); ^13^C NMR (CDCl_3_) δ 193.4, 179.1, 161.5, 158.7, 139.0, 138.0, 137.4, 130.5, 129.8, 129.2, 127.1, 125.6, 125.5, 123.9, 122.9, 120.8, 118.6, 113.7, 52.2, 47.8, 44.5, 38.1, 37.8, 36.7, 29.3, 24.7, 21.8, 18.6, 16.8; HR-ESI: *m/z* 475.1925; [M + H^+^] for the compound C_25_H_29_SO_4_ requires 475.1938.

Tert-butyl 4-(4-((E)-3-((4bS,8R)-3-methoxy-8-(methoxycarbonyl)-4b,8-dimethyl-4b,5, 6,7,8,8a,9,10-octahydrophenanthren-2-yl)acryloyl)phenyl)piperazine-1-carboxylate (**48**): 88%; MS (ESI, 30eV): *m/z* 625.37 [M + Na], 603.61 [M + H]; ^1^H NMR (CDCl_3_) δ 8.03–7.99 (m, 2H), 7.90 (d, *J* = 9.1 Hz, 1H), 7.58 (d, *J* = 15.8 Hz, 1H), 7.28 (s, 1H), 7.07 (d, *J* = 8.2 Hz, 1H), 6.80 (s, 1H), 3.88 (s, 3H), 3.68 (s, 3H), 3.63 (t, *J* = 5.3 Hz, 2H), 3.40–3.31 (m, 5H), 2.89–2.84 (m, 2H), 2.29 (d, *J* = 12.4 Hz, 1H), 2.23 (dd, *J* = 12.5, 2.2 Hz, 1H), 1.87–1.72 (m, 5H), 1.68–1.65 (m, 1H), 1.57–1.52 (m, 1H), 1.49 (s, 9H), 1.46–1.41 (m, 1H), 1.29 (s, 3H), 1.24 (s, 3H); ^13^C NMR (CDCl_3_) δ 196.6, 189.1, 179.1, 157.3, 154.7, 153.5, 139.6, 130.8, 130.6, 130.0, 127.5, 125.4, 122.1, 115.2, 114.5, 107.2, 80.5, 55.8, 52.1, 47.8, 44.8, 38.1, 38.0, 36.7, 29.1, 28.5, 26.3, 25.0, 21.8, 21.6, 18.7, 16.7.

(1R,4aS)-methyl 6-hydroxy-1,4a-dimethyl-7-((E)-3-oxo-3-(pyridin-2-yl)prop-1-en-1-yl)-1,2,3,4,4a,9,10,10a-octahydrophenanthrene-1-carboxylate (**49**): 74%; ^1^H NMR (CDCl_3_) δ 10.65 (s, 1H), 9.79 (s, 1H), 7.45 (d, *J* = 8.0 Hz, 1H), 7.34–7.29 (m, 1H), 7.28–7.26 (m, 2H), 7.21 (s, 1H), 6.87 (s, 1H), 3.68 (s, 3H), 2.90–2.85 (m, 2H), 2.26 (d, *J* = 12.9 Hz, 1H), 2.19 (dd, *J* = 12.5, 2.4 Hz, 1H), 1.86–1.64 (m, 5H), 1.53–1.43 (m, 2H), 1.28 (s, 3H), 1.22 (s, 3H); ^13^C NMR (CDCl_3_) δ 195.9, 182.9, 182.0, 164.6, 15.9, 159.4, 137.5, 136.6, 134.0, 127.9, 127.0, 119.0, 112.9, 92.0, 52.0, 47.6, 44.1, 38.2, 37.6, 36.5, 28.7, 24.5, 21.4, 18.4, 16.6.

(1R,4aS)-methyl 6-hydroxy-1,4a-dimethyl-7-((E)-3-oxo-3-(pyridin-3-yl)prop-1-en-1-yl)-1,2,3,4,4a,9,10,10a-octahydrophenanthrene-1-carboxylate (**50**): 65%; MS (ESI, 30eV): *m/z* 442.47 [M + Na]; ^1^H NMR (CDCl_3_) δ 9.22 (d, *J* = 46.1 Hz, 1H), 8.79 (br s, 1H), 8.37–8.24 (m, 1H), 8.00 (d, *J* = 15.7 Hz, 1H), 7.75 (d, *J* = 15.7 Hz, 1H), 7.58 (s, 1H), 7.51–7.47 (m, 1H), 7.46–7.42 (m, 1H),. 6.87 (s, 1H), 3.68 (s, 3H), 2.87–2.81 (m, 2H), 2.26 (d, *J* = 12.5 Hz, 1H), 2.21 (dd, *J* = 12.5, 2.4 Hz, 1H), 1.80–1.64 (m, 5H), 1.54–1.41 (m, 2H), 1.28 (s, 3H), 1.25 (s, 3H); ^13^C NMR (CDCl_3_) δ 195.9, 159.9, 159.4, 137.3, 136.6, 134.0, 128.6, 127.9, 126.4, 117.0, 112.9, 52.0, 47.6, 44.1, 38.2, 37.6, 36.5, 28.7, 24.5, 21.6, 18.4, 16.6.

(1R,4aS)-methyl 6-hydroxy-1,4a-dimethyl-7-((E)-3-oxo-3-(pyrazin-2-yl)prop-1-en-1-yl)-1,2,3,4,4a,9,10,10a-octahydrophenanthrene-1-carboxylate (**51**): 69%; MS (ESI, 30eV): *m/z* 459.56 [M + K], 421.44 [M + H]; ^1^H NMR (CDCl_3_) *δ* 9.25 (s, 1H), 8.78 (d, *J* = 2.4 Hz, 1H), 8.75 & 8.71 (2d, *J* = 2.6 & 2.5 Hz, 1H), 8.67–8.46 (m, 1H), 7.56 (s, 1H), 7.17 (d, *J* = 7.3 Hz, 2H), 6.87 (s, 1H), 3.68 (s, 3H), 2.87–2.80 (m, 2H), 2.25 (d, *J* = 12.3 Hz, 1H), 2.19 (dd, *J* = 12.5, 2.7 Hz, 1H), 1.84–1.61 (m, 7H), 1.28 (s, 3H), 1.21 (s, 3H); ^13^C NMR (CDCl_3_) *δ* 178.9, 159.9, 159.1, 147.9, 143.6, 142.9, 142.5, 137.9, 130.9, 129.0, 128.2, 126.4, 125.3, 112.8, 52.0, 47.6, 44.2, 37.9, 37.6, 36.5, 28.7, 24.6, 21.4, 18.4, 16.6; HR-ESI: *m/z* 421.2117; [M + H^+^] for the compound C_25_H_29_N_2_O_4_ requires 421.2122.

(1R,4aS)-methyl 7-((E)-3-(benzofuran-2-yl)acryloyl)-6-hydroxy-1,4a-dimethyl-1,2,3, 4,4a,9,10, 10a-octahydrophenanthrene-1-carboxylate (**52**): 74%; MS (ESI, 30eV): *m/z* 481.21 [M + Na], 459.18 [M + H]; ^1^H NMR (CDCl_3_) δ 7.53 (dd, *J* = 7.6, 3.5 Hz, 1H), 7.45 (dd, *J* = 8.1, 2.8 Hz, 1H), 7.38–7.35 (m, 1H), 7.29 (dd, *J* = 8.2, 4.0 Hz, 1H), 7.22–7.19 (m, 1H), 7.10 (t, *J* = 7.1 Hz, 1H), 7.05 (t, *J* = 7.1 Hz, 1H), 6.68 (s, 1H), 6.23 (s, 1H), 3.67 (s, 3H), 3.63 (s, 1H), 2.80–2.73 (m, 1H), 2.56 (dd, *J* = 6.5, 2.4 Hz, 1H), 2.54 (dd, *J* = 6.5, 2.4 Hz, 1H), 1.69–1.63 (m, 2H), 1.51–1.44 (m, 2H), 1.41–1.35 (m, 1H), 1.25–1.18 (m, 2H), 1.11 (s, 3H), 1.10 (s, 3H); ^13^C NMR (CDCl_3_) δ 196.5, 179.0, 161.4, 155.7, 154.6, 152.2, 149.5, 128.8, 128.7, 128.4, 127.9, 126.4, 123.3, 122.8, 122.7, 112.2, 111.1, 103.1, 75.5, 51.9, 47.5, 44.4, 37.4, 36.7, 36.3, 21.6, 21.4, 18.3, 16.4.

(1R,4aS)-methyl 7-((E)-3-(5-bromothiophen-2-yl)-3-oxoprop-1-en-1-yl)-6-hydroxy-1,4a-dimthyl-1,2,3,4,4a,9,10,10a-octahydrophenanthrene-1-carboxylate (**53**): 73%; MS (ESI, 30eV): *m/z* 541.28 [M + K], 525.98 [M + Na], 503.12 [M + H]; ^1^H NMR (CDCl_3_) δ 8.02 (d, *J* = 15.5 Hz, 1H), 7.58 (d, *J* = 4.0 Hz, 1H), 7.44 (d, *J* = 15.6 Hz, 1H), 7.12 (d, *J* = 4.0 Hz, 1H), 6.74 (s, 1H), 5.89 (s, 1H), 3.68 (s, 3H), 2.87–2.78 (m, 2H), 2.22–2.16 (m, 2H), 1.85–1.63 (m, 5H), 1.52–1.46 (m, 1H), 1.45–1.40 (m, 1H), 1.27 (s, 3H), 1.20 (s, 3H); ^13^C NMR (CDCl_3_) δ 181.8, 179.0, 153.8, 147.4, 140.4, 131.7, 131.3, 130.4, 129.0, 128.2, 122.4, 120.7, 119.6, 112.1, 52.0, 47.6, 44.4, 37.8, 37.5, 36.5, 28.8, 24.7, 21.6, 18.5, 16.5.

### 3.3. Biological Assays

MCF-7 (ER-positive breast cancer cells, epithelial, low metastatic), MDA-MB-231, and Hs578T (triple negative breast cancer cells, mesenchymal, high metastatic) were obtained from American Type Culture Collection (ATCC) and cultured with DMEM high glucose/10% Fetal Bovine Serum (FBS) (supplied by Biosera). Primary skin normal fibroblasts were kindly provided by Prof. Emer. A. Aletras, Laboratory of Biochemistry, University of Patras, Greece and cultured with DMEM low glucose/10% FBS (supplied by Biosera). Cancer cells were seeded in 24 well plates (30,000 MCF-7 cells/well, 30.000 MDA-MB-231 cells/well, 25,000 Hs578T cells/well) and fibroblasts in 12 well plates (40,000 cells/well) and incubated in proper medium completed with 10% FBS for 48 h. Compounds were diluted in DMSO and working dilutions were prepared ranging from 5 to 200 μΜ for cancer cells and 1 to 100 μM for fibroblasts. All cells were incubated with working dilutions for 48 h. Afterwards, cells were detached by trypsinization, centrifuged at 3000 rpm for 3 min, and counted using a hemocytometer. Antiproliferation values were then calculated relatively to cancer or normal cells treated only with DMSO. The DMSO concentration equivalents were tested for their toxicity in all cells and no statistical significance at the cellular viability was observed at the used concentrations. IC_50_ values were calculated using GraphPad Prism 5 (Graph Pad Software).

## 4. Conclusions

In conclusion, from the total of 23 synthetic DHA-chalcone hybrids tested, two compounds, **33** and **41**, exhibited high activity levels in all three breast cancer cell lines, namely the estrogen-dependent MCF-7 and the estrogen-independent MDA-MB-231 and Hs578T. Four DHA-chalcone hybrids (**38**, **43**, **44**, and **47**) present selective strong antiproliferative activity only against MCF-7 breast cancer cells. Finally, one compound (**51**) displays a selective moderate anticancer activity against the triple negative cell lines MDA-MB-231 and Hs578T. Compound **38** appears to be a promising drug lead for the treatment of hormone dependent breast cancer cases, which account for the majority of breast cancers, taking into consideration it’s comparable activity with the control drug 5-FU on MCF-7 breast cancer cells and its very high selectivity index (SI = 22.7), which makes it 12.9 times less toxic than 5-FU. Further studies aiming to reveal new hybrid lead compounds are currently in progress, focusing on the structural elements of the chalcone’s ring B (taking into consideration the so far SARS) and the replacement of the C18 ester moiety with various amides and carbamates.

## Figures and Tables

**Table 1:** Antiproliferation activity of synthesized compounds **32**–**54** in the MDA-MB-231, MCF-7 and Hs578T breast cancer cell lines as well as against normal human fibroblast cell line. Selectivity index values are also provided for each IC_50_ value.

Compound	IC_50_ (μM)				Selectivity Index for		
	MDA-MB-231	MCF-7	Hs578T	Fibroblasts	MDA-MB-231	MCF-7	Hs578T
**31**	>75	23.90 ± 4.47	>75	n/a	n/a	n/a	n/a
**32**	>75	>75	>75	n/a	n/a	n/a	n/a
**33**	5.89 ± 0.77	2.21 ± 0.40	2.48 ± 0.13	8.68 ± 0.88	1.47	3.93	3.50
**34**	>75	21.36 ± 0.60	28.09 ± 7.76	n/a	n/a	n/a	n/a
**35**	37.05 ± 1.82	9.90 ± 0.05	8.69 ± 0.55	33.83 ± 6.63	0.91	3.42	3.89
**36**	>75	>75	>75	n/a	n/a	n/a	n/a
**37**	>75	11.39 ± 1.55	>75	n/a	n/a	n/a	n/a
**38**	73.00 ± 2.31	7.06 ± 1.71	>75	160.65 ± 13.08	2.20	22.75	-
**39**	>75	11.50 ± 1.02	15.71 ± 2.74	32.88 ± 2.27	-	2.86	2.09
**40**	>75	>75	>75	92.11 ± 5.47	-	-	-
**41**	7.12 ± 0.20	3.99 ± 0.15	10.31 ± 0.25	7.96 ± 0.10	1.12	1.99	0.77
**42**	>75	37.01 ± 6.89	43.33 ± 4.32	n/a	n/a	n/a	n/a
**43**	29.20 ± 3.26	4.29 ± 0.29	16.64 ± 2.83	33.05 ± 6.10	1.13	7.70	1.99
**44**	25.21 ± 0.84	3.45 ± 0.36	21.48 ± 1.20	44.03 ± 1.84	1.75	12.76	2.05
**45**	>75	29.94 ± 5.26	>75	n/a	n/a	n/a	n/a
**46**	>75	>75	>75	n/a	n/a	n/a	n/a
**47**	40.37 ± 0.67	12.52 ± 8.43	>75	n/a	n/a	n/a	n/a
**48**	>75	25.62 ± 0.09	23.15 ± 2.50	n/a	n/a	n/a	n/a
**49**	22.43 ± 0.81	45.93 ± 1.52	17.07 ± 2.13	23.81 ± 1.07	1.06	0.52	1.39
**50**	>75	>75	>75	192.25 ± 51.41	-	-	-
**51**	18.01 ± 2.78	>75	23.11 ± 0.04	20.47 ± 2.46	1.14	-	0.88
**52**	>75	>75	60.21 ± 6.94	n/a	n/a	n/a	n/a
**53**	17.12 ± 0.41	41.44 ± 1.74	28.44 ± 1.97	n/a	n/a	n/a	n/a
**28**	115.44 ± 40.11	206.95 ± 7.28	135.95 ± 2.47	n/a	n/a	n/a	n/a
**5-FU**	19.17 ± 0.39	5.37 ± 1.16	7.38 ± 2.49	9.43 ± 0.80	0.49	1.76	1.28

## Data Availability

Not applicable.

## Supplementary Materials

The following supporting information can be downloaded at: https://www.mdpi.com/article/10.3390/molecules27113623/s1. Figure S1–S54: Copies of ^1^H and ^13^C NMR spectra of compounds.

## Sample Availability

Samples of the compounds are not available from the authors.
