# Supplementary material for: Synthesis and Antiproliferative Activity of Novel Dehydroabietic Acid-Chalcone Hybrids
Source: Molecules. 2022 Jun 5;27(11):3623. doi: 10.3390/molecules27113623 (PMC9181926; doi:10.3390/molecules27113623)
Supplement: Supplementary file 1 [file molecules-27-03623-s001.zip › molecules-1724271-supplementary.pdf]

SUPPORTING INFORMATION

Synthesis and Antiproliferative Activity of Novel Dehydroabietic Acid- Chalcone hybrids

Sophia Grigoropoulou [1], Dimitra Manou [2], Antonia I. Antoniou [1], Artemis Tsirogianni [1], Carlo Siciliano [3], Achilleas D. Theocharis [2,]* and Constantinos M. Athanassopoulos [1,]*

[1] Synthetic Organic Chemistry Laboratory, Department of Chemistry,University of Patras, GR-26504 Patras, Greece; grigorsophia@hotmail.com (S.G.); tonadoniou@upatras.gr (A.I.A.); kath@chemistry.upatras.gr (C.M.A.)

[2] Biochemistry, Biochemical Analysis & Matrix Pathobiology Research Group, Laboratory of Biochemistry, Department of Chemistry, University of Patras, GR-26504 Patras, Greece; dimitramanou@outlook.com (D.M.); atheoch@upatras.gr (A.D.T.)

[3] Department of Pharmacy, Health and Nutritional Sciences, Edificio Polifunzionale, I-87036 Arcavacata di Rende (CS), Italy; carlo.siciliano@unical.it (C.S)

* Correspondence: kath@chemistry.upatras.gr; Tel.: +30-2610-997909 and atheoch@upatras.gr; Tel.: +30-2610-996029

Copies of NMR spectra

Table of contents

Figure S1. ^{1}H-NMR spectrum of compound 31. 4
Figure S2. ^{13}C-NMR spectrum of compound 31. 4
Figure S3. ^{1}H-NMR spectrum of compound 32. 5
Figure S4. ^{13}C-NMR spectrum of compound 32. 5
Figure S5. ^{1}H-NMR spectrum of compound 33. 6
Figure S6. ^{13}C-NMR spectrum of compound 33. 6
Figure S7. APT-NMR spectrum of compound 33. 7
Figure S8. ^{1}H-NMR spectrum of compound 34. 7
Figure S9. ^{13}C-NMR spectrum of compound 34. 8
Figure S10. ^{1}H-NMR spectrum of compound 35. 8
Figure S11. ^{13}C-NMR spectrum of compound 35. 9
Figure S12. ^{1}H-NMR spectrum of compound 36. 9
Figure S13. ^{13}C-NMR spectrum of compound 36. 10
Figure S14. ^{1}H-NMR spectrum of compound 37. 10
Figure S15. ^{13}C-NMR spectrum of compound 37. 11
Figure S16. APT-NMR spectrum of compound 37. 11
Figure S17. ^{1}H-NMR spectrum of compound 38. 12
Figure S18. ^{13}C-NMR spectrum of compound 38. 12
Figure S19. APT-NMR spectrum of compound 38. 13
Figure S20. ^{1}H-NMR spectrum of compound 39. 13
Figure S21. ^{13}C-NMR spectrum of compound 39. 14
Figure S22. ^{1}H-NMR spectrum of compound 40. 14
Figure S23. ^{13}C-NMR spectrum of compound 40. 15
Figure S24. ^{1}H-NMR spectrum of compound 41. 15
Figure S25. ^{13}C-NMR spectrum of compound 41. 16
Figure S26. APT-NMR spectrum of compound 41. 16
Figure S27. ^{1}H-NMR spectrum of compound 42. 17
Figure S28. ^{13}C-NMR spectrum of compound 42. 17
Figure S29. ^{1}H-NMR spectrum of compound 43. 18

Figure S30. ^{13}C-NMR spectrum of compound 43. 18
Figure S31. APT-NMR spectrum of compound 43. 19
Figure S32. ^{1}H-NMR spectrum of compound 44. 19
Figure S33. ^{13}C-NMR spectrum of compound 44. 20
Figure S34. APT-NMR spectrum of compound 44. 20
Figure S35. ^{1}H-NMR spectrum of compound 45. 21
Figure S36. ^{13}C-NMR spectrum of compound 45. 21
Figure S37. ^{1}H-NMR spectrum of compound 46. 22
Figure S38. ^{13}C-NMR spectrum of compound 46. 22
Figure S39. ^{1}H-NMR spectrum of compound 47. 23
Figure S40. ^{13}C-NMR spectrum of compound 47. 23
Figure S40. APT-NMR spectrum of compound 47. 24
Figure S42. ^{1}H-NMR spectrum of compound 48. 24
Figure S43. ^{13}C-NMR spectrum of compound 48. 25
Figure S44. ^{1}H-NMR spectrum of compound 49. 25
Figure S45. ^{13}C-NMR spectrum of compound 49. 26
Figure S46. ^{1}H-NMR spectrum of compound 50. 26
Figure S47. ^{13}C-NMR spectrum of compound 50. 27
Figure S48. ^{1}H-NMR spectrum of compound 51. 27
Figure S49. ^{13}C-NMR spectrum of compound 51. 28
Figure S50. APT-NMR spectrum of compound 51. 28
Figure S51 ^{1}H-NMR spectrum of compound 52. 29
Figure S52. ^{13}C-NMR spectrum of compound 52. 29
Figure S53. ^{1}H-NMR spectrum of compound 53. 30
Figure S54. ^{13}C-NMR spectrum of compound 53. 30

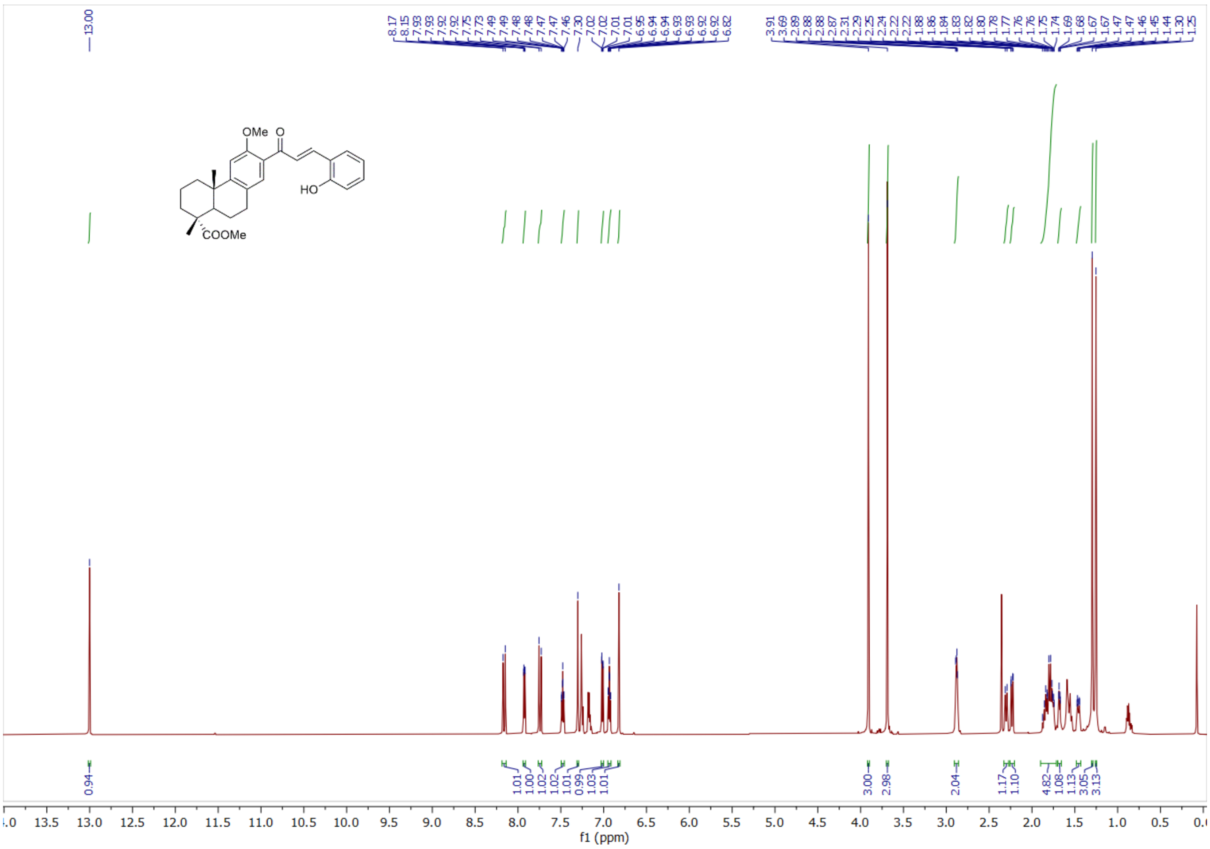

Figure S1. ¹H-NMR spectrum of compound 31.

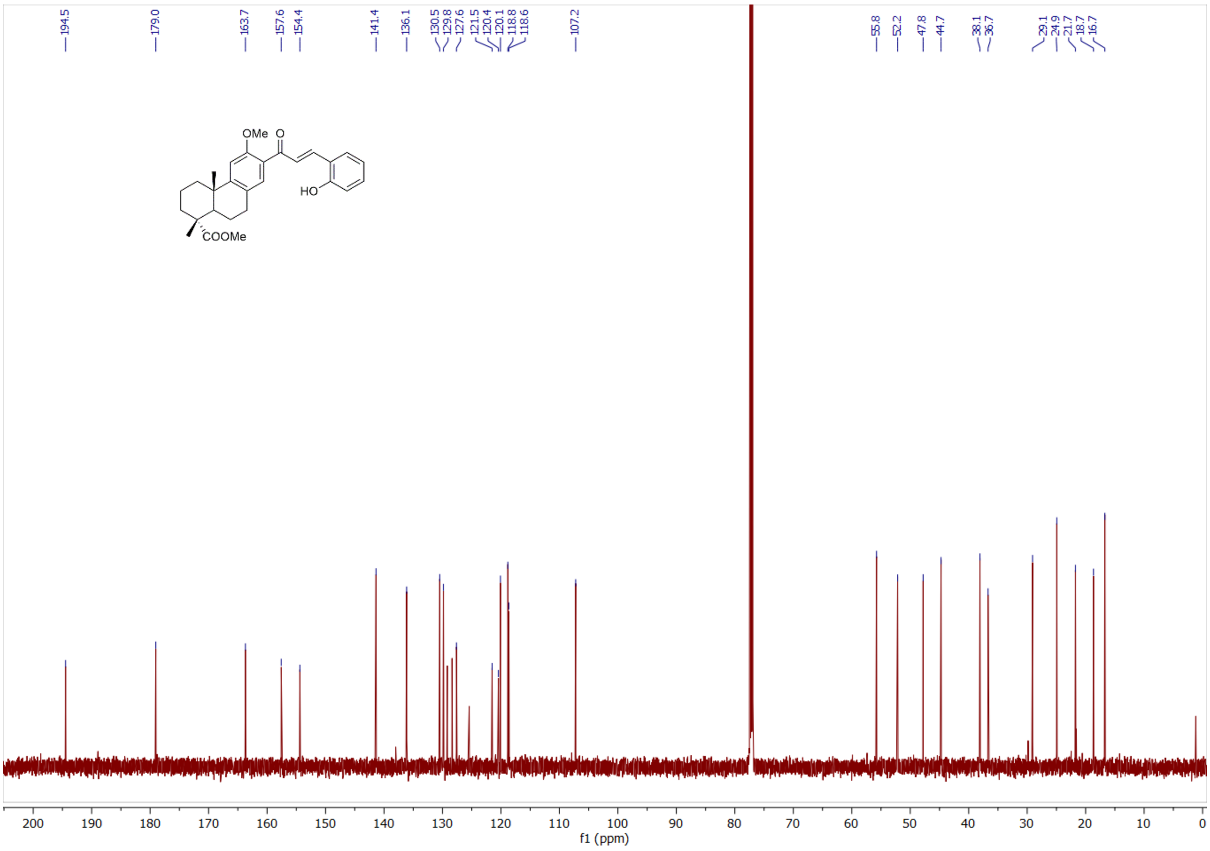

Figure S2. ¹³C-NMR spectrum of compound 31.

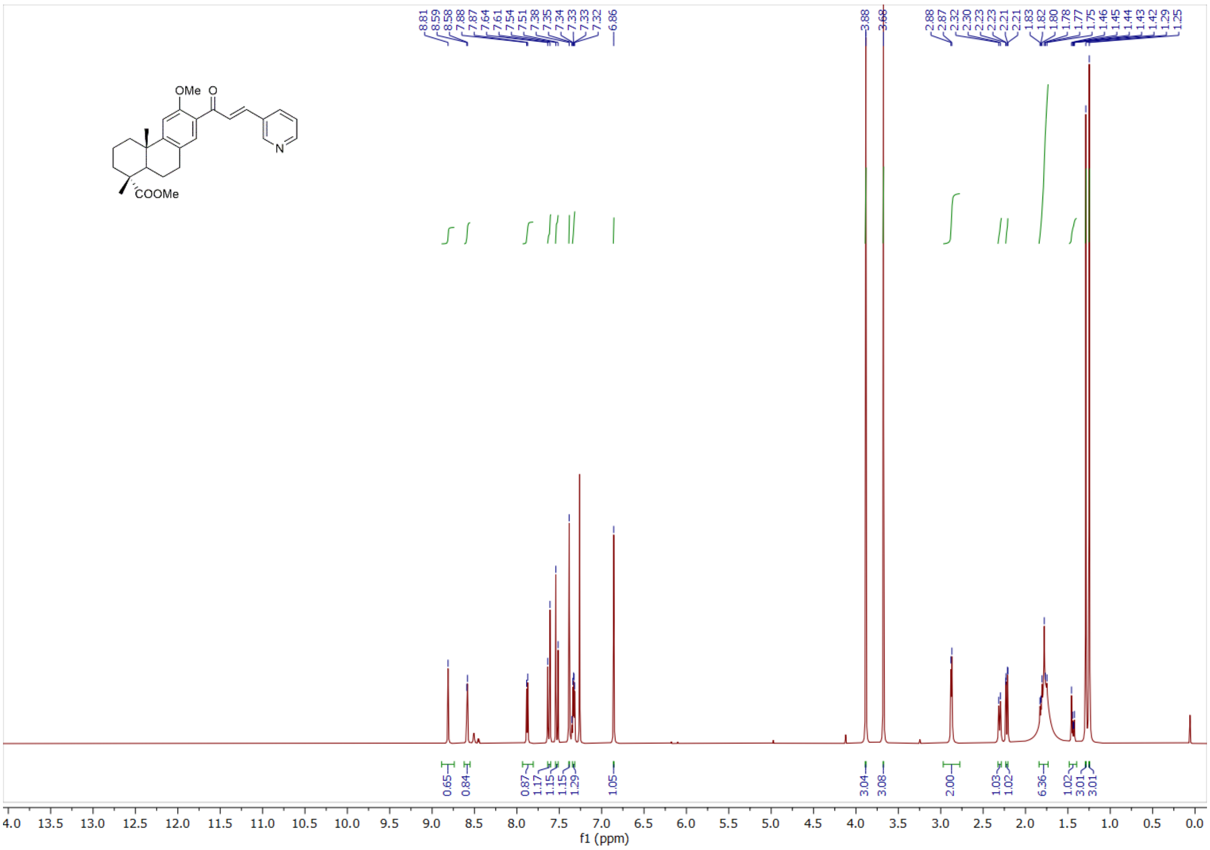

Figure S3. **^{1}H-NMR spectrum of compound 32.**

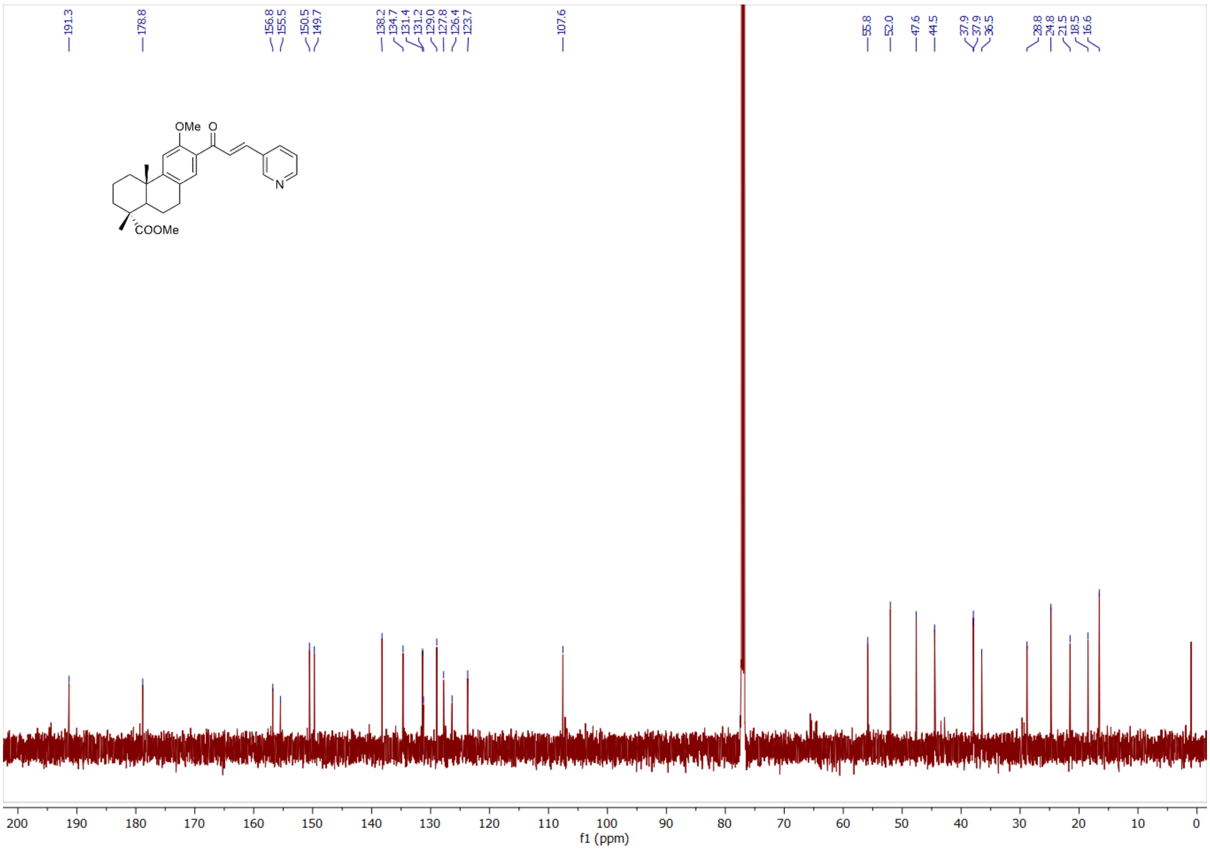

Figure S4. **^{13}C-NMR spectrum of compound 32.**

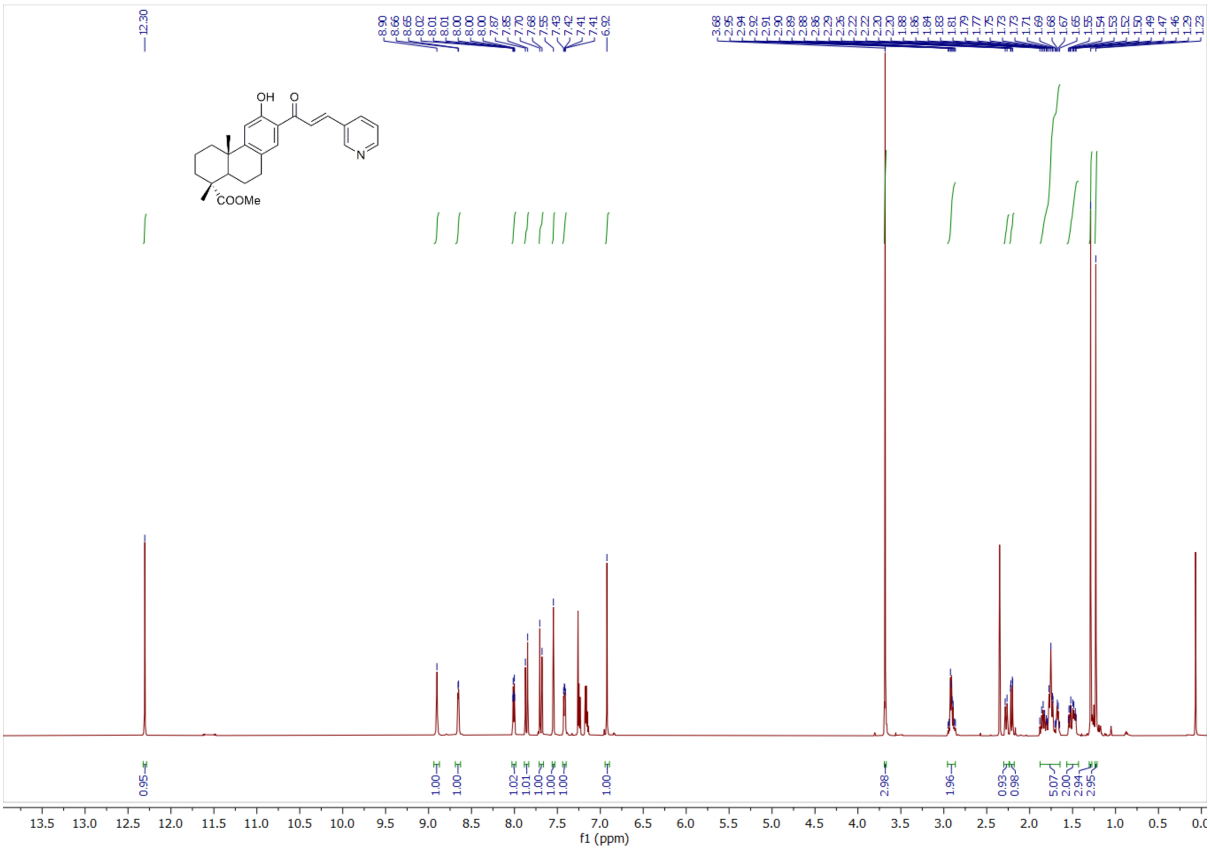

Figure S5. ^{1}H-NMR spectrum of compound 33.

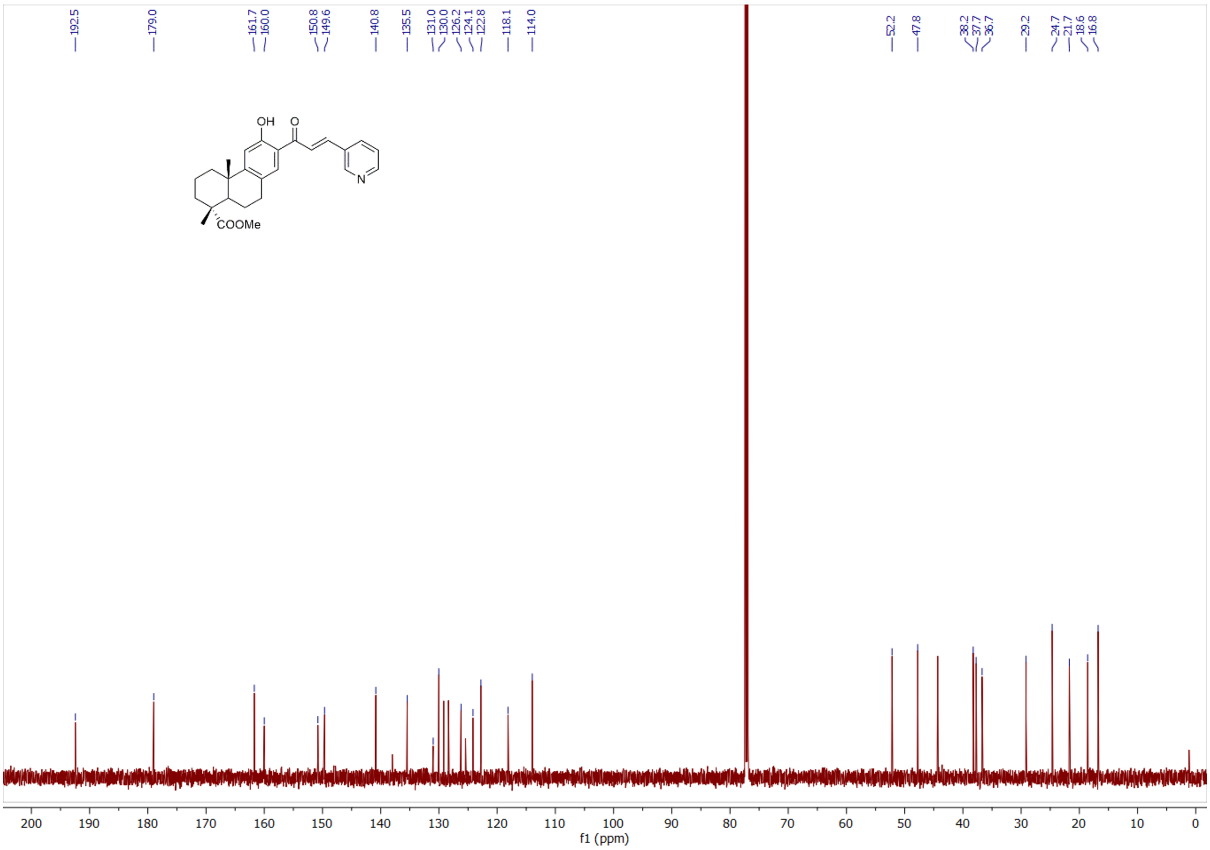

Figure S6. ^{13}C-NMR spectrum of compound 33.

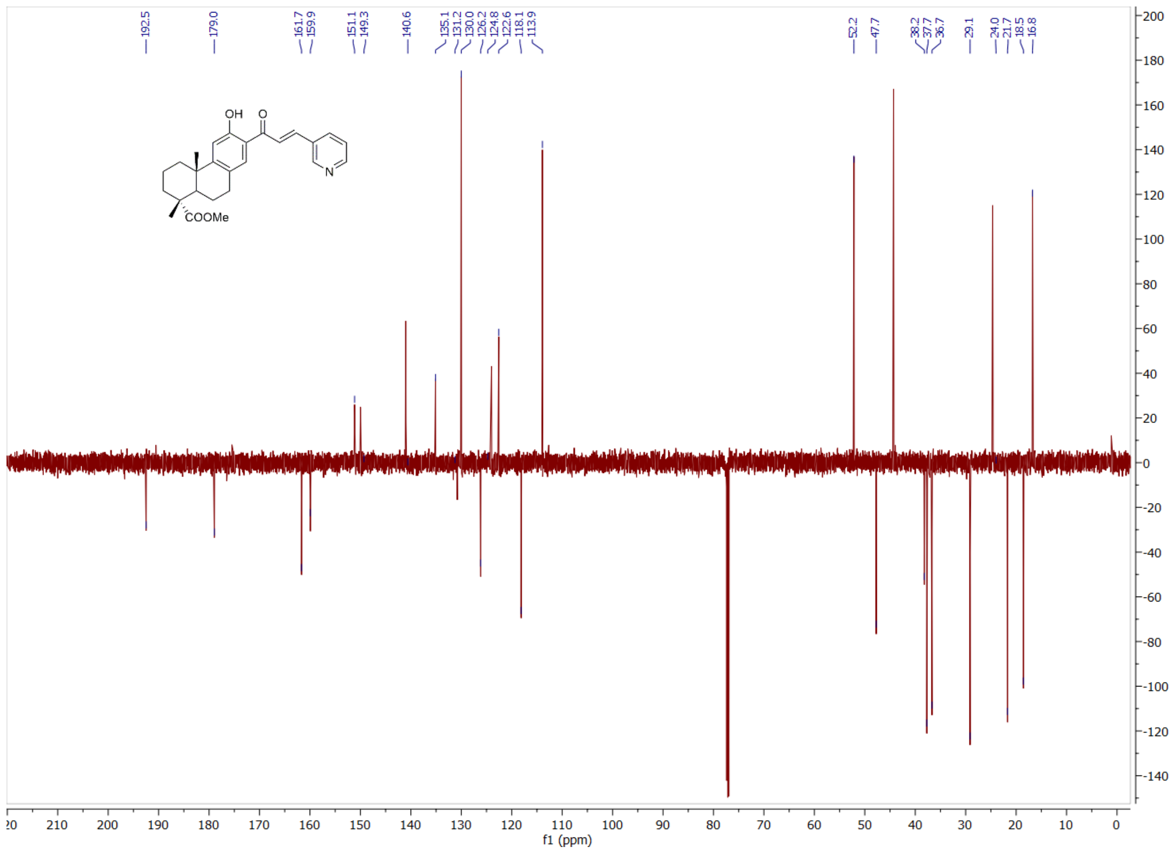

Figure S7. APT-NMR spectrum of compound 33.

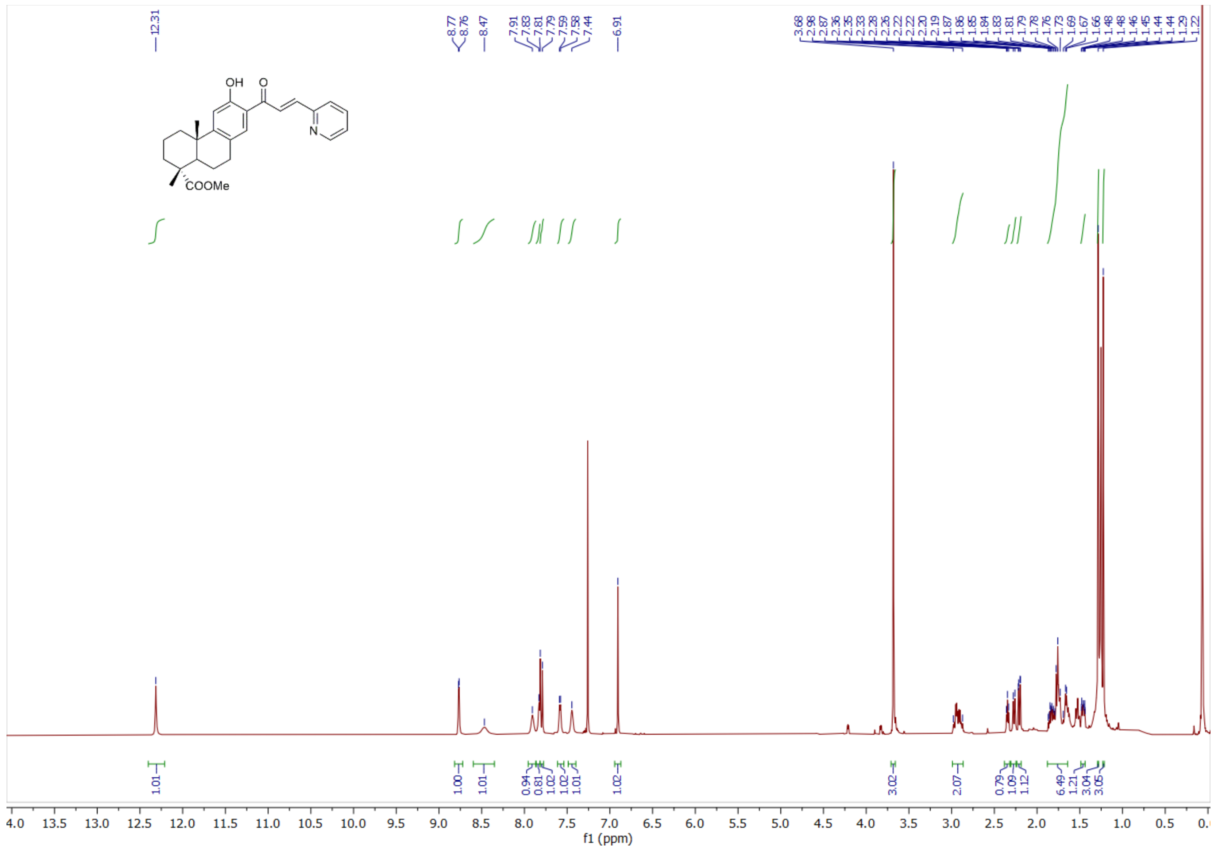

Figure S8. ^{1}H-NMR spectrum of compound 34.

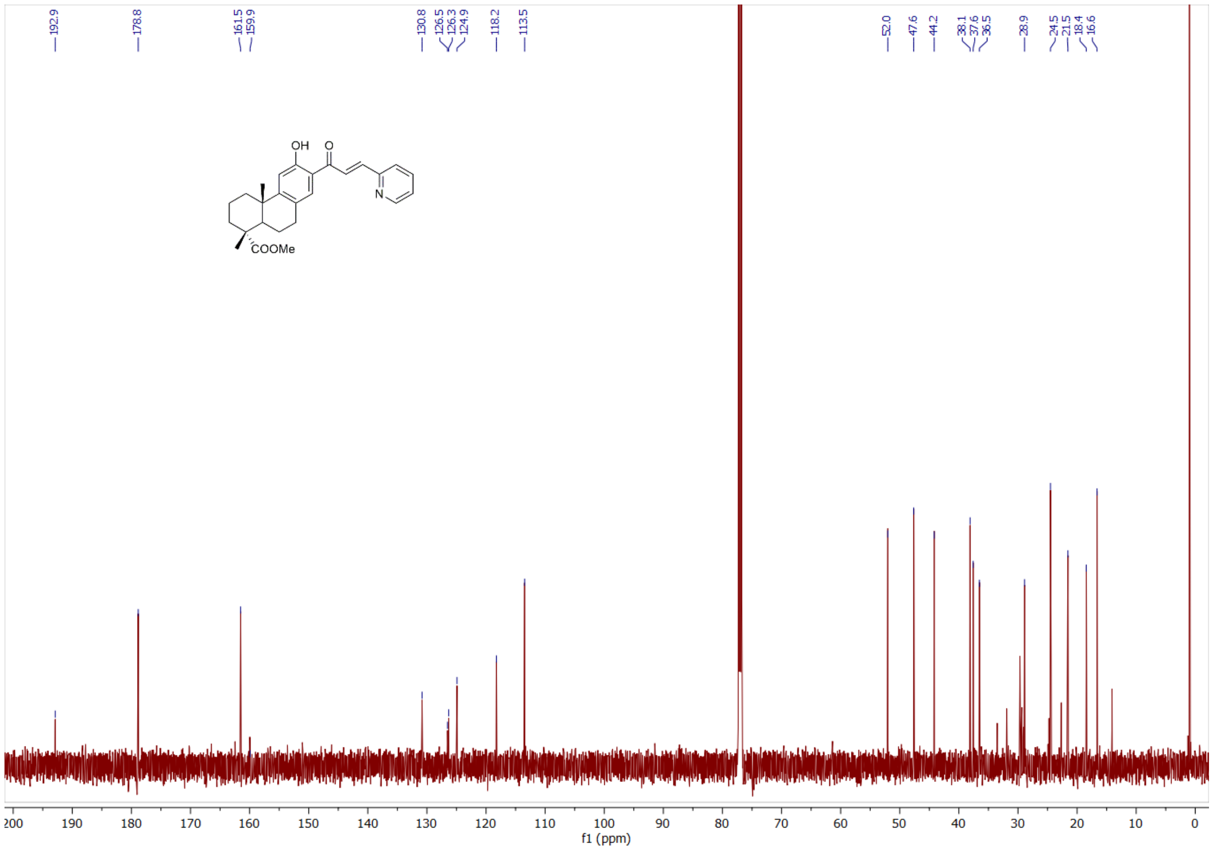

Figure S9. ^{13}C-NMR spectrum of compound 34.

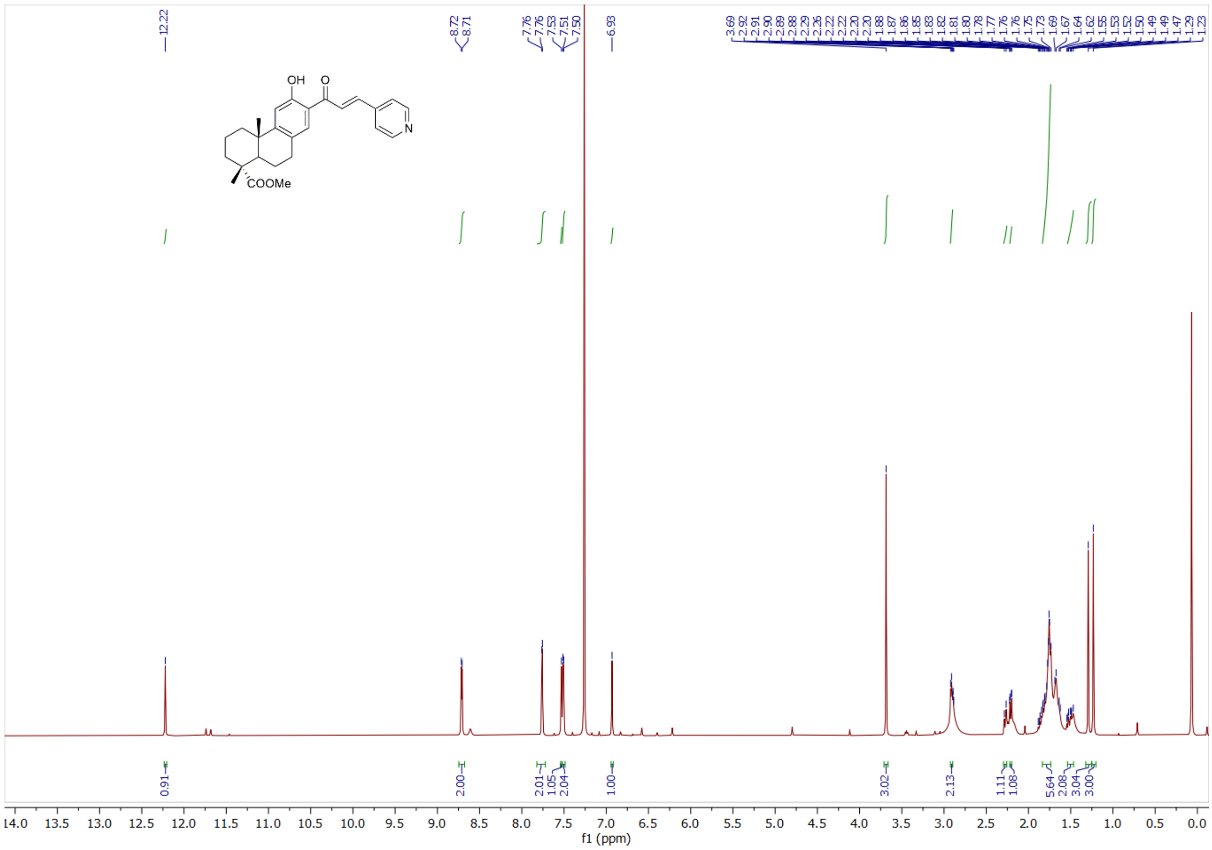

Figure S10. ^{1}H-NMR spectrum of compound 35.

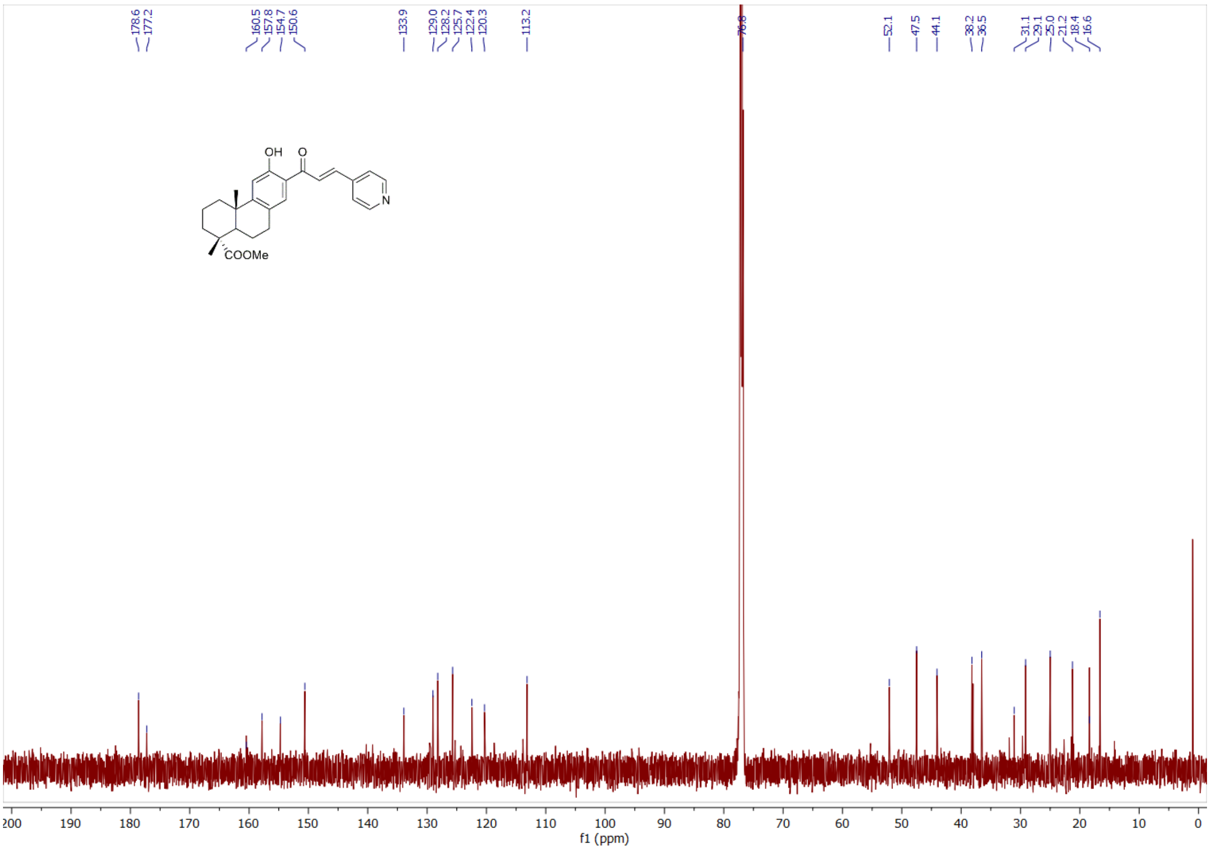

Figure S11. ^{13}**C-NMR spectrum of compound 35.**

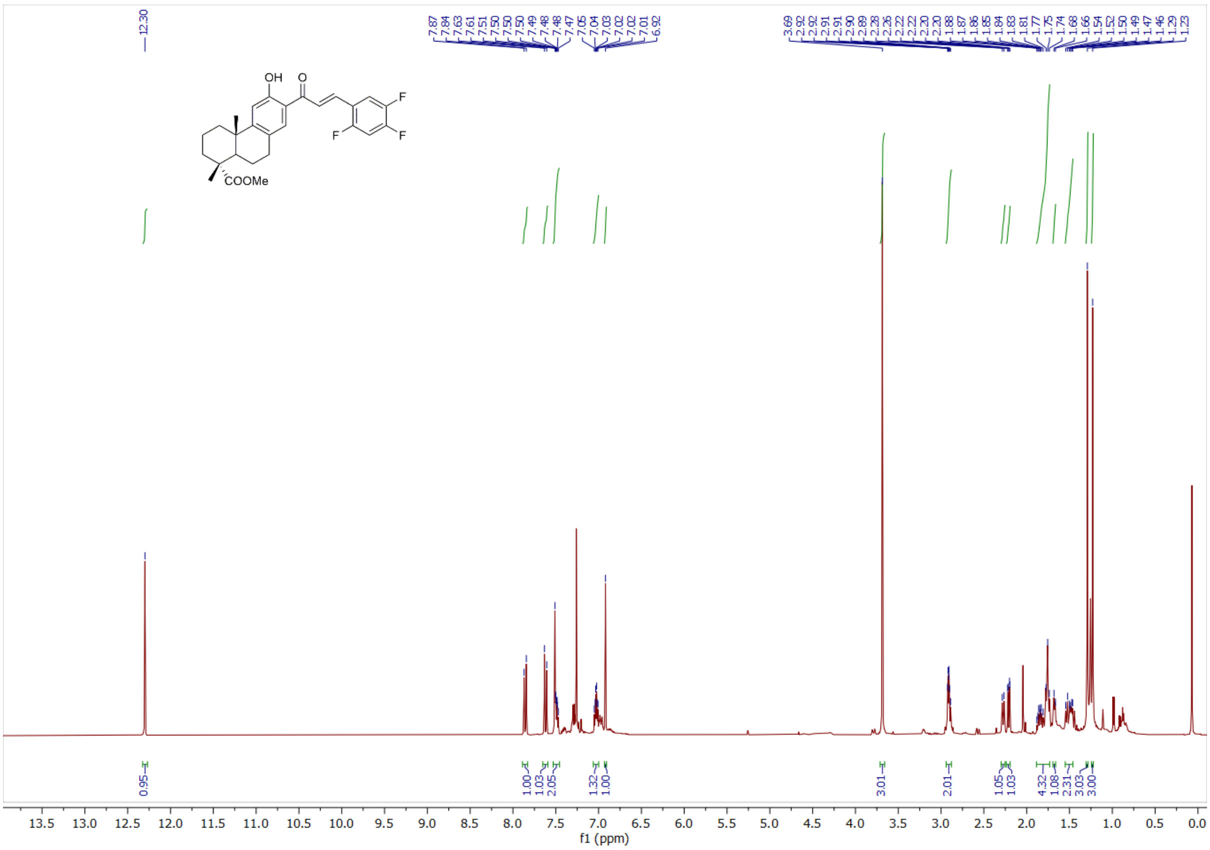

Figure S12. ^{1}**H-NMR spectrum of compound 36.**

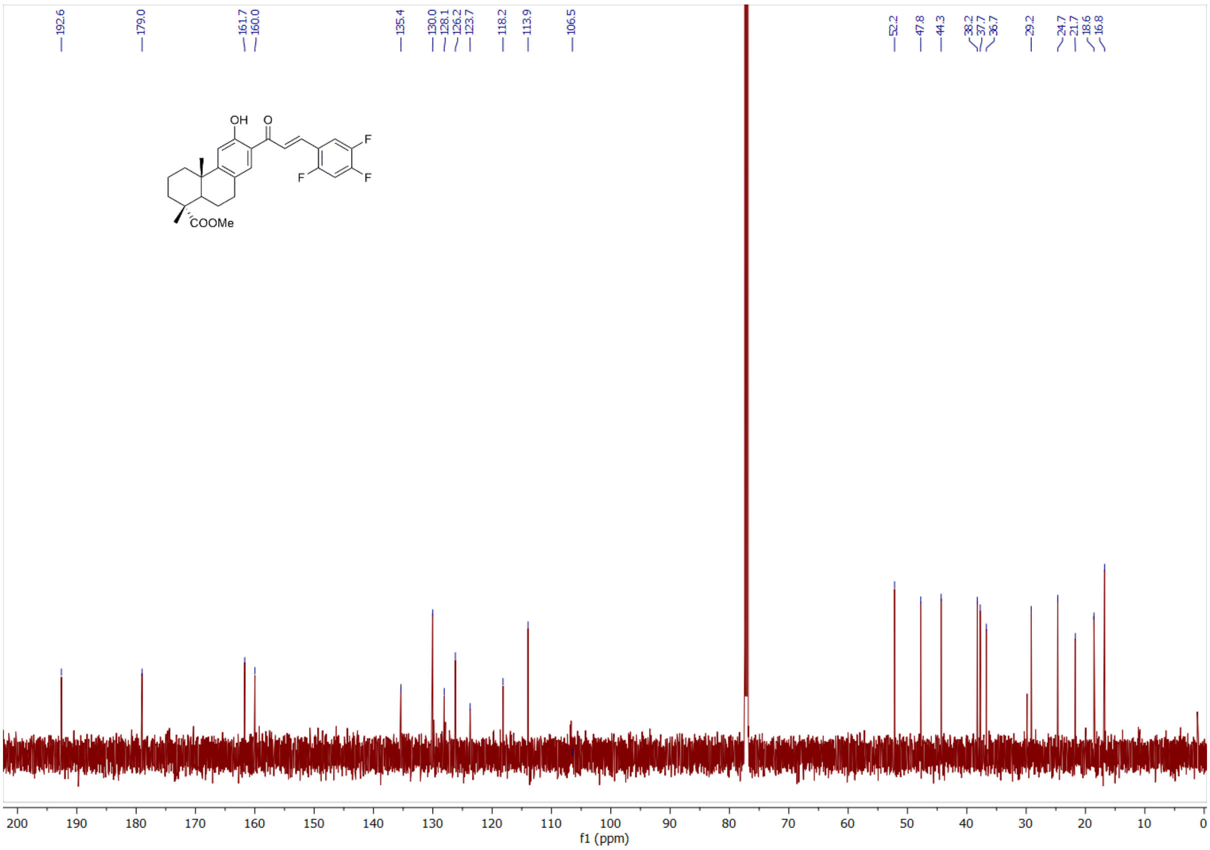

Figure S13. ^{13}C-NMR spectrum of compound 36.

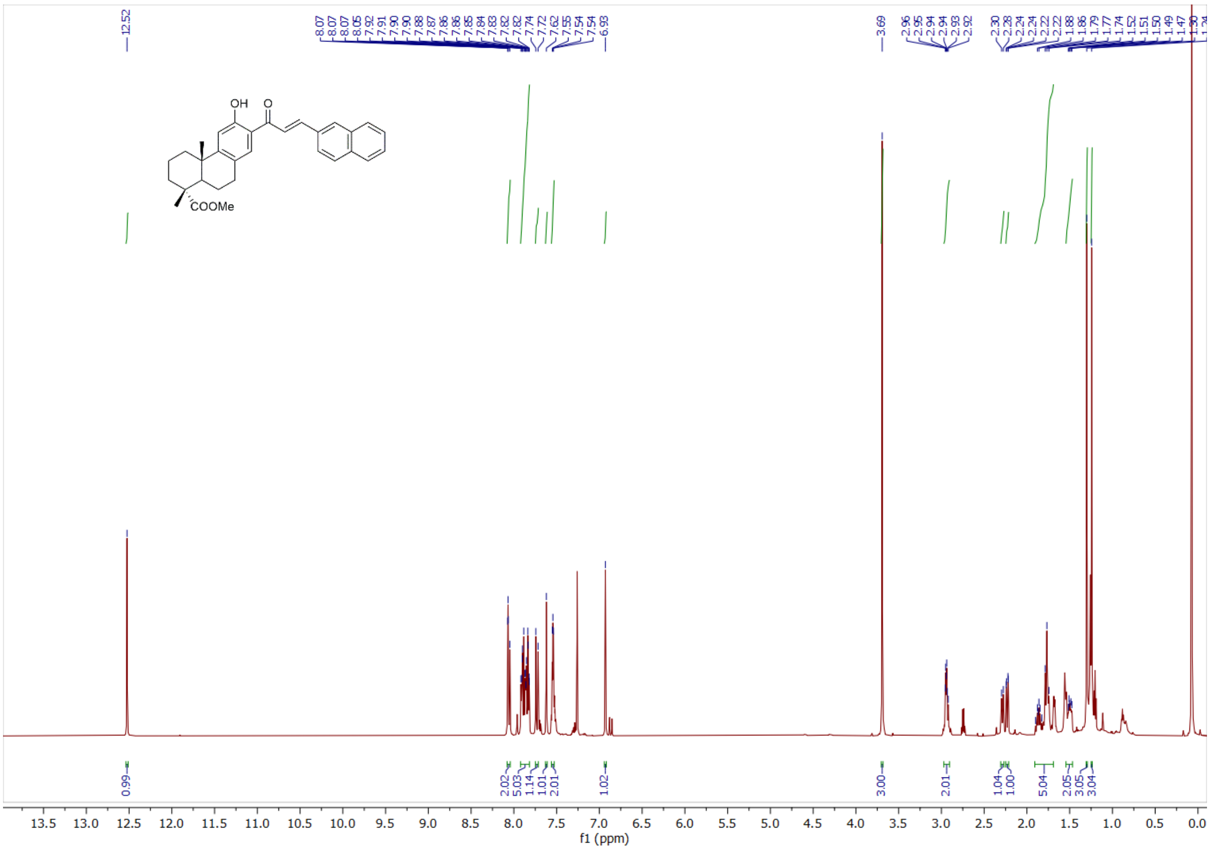

Figure S14. ^{1}H-NMR spectrum of compound 37.

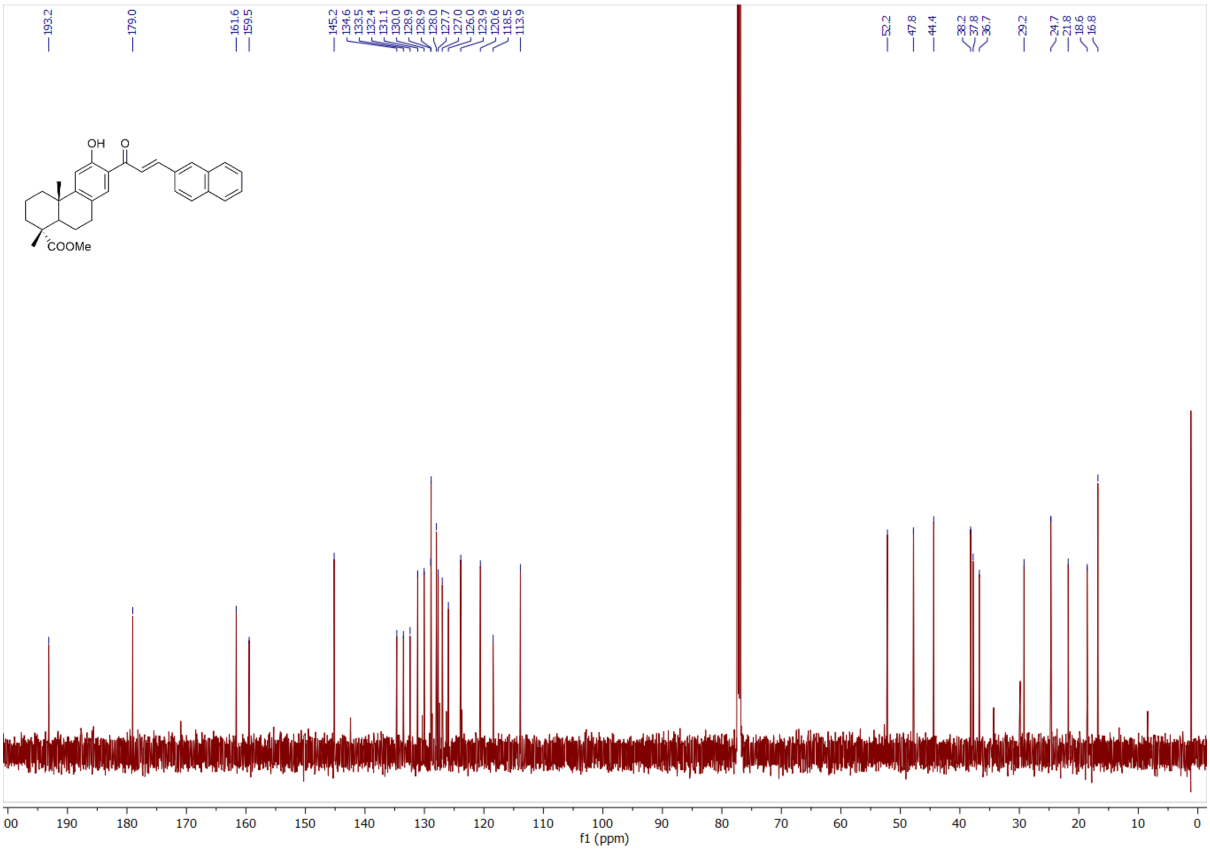

Figure S15. ^{13}C-NMR spectrum of compound 37.

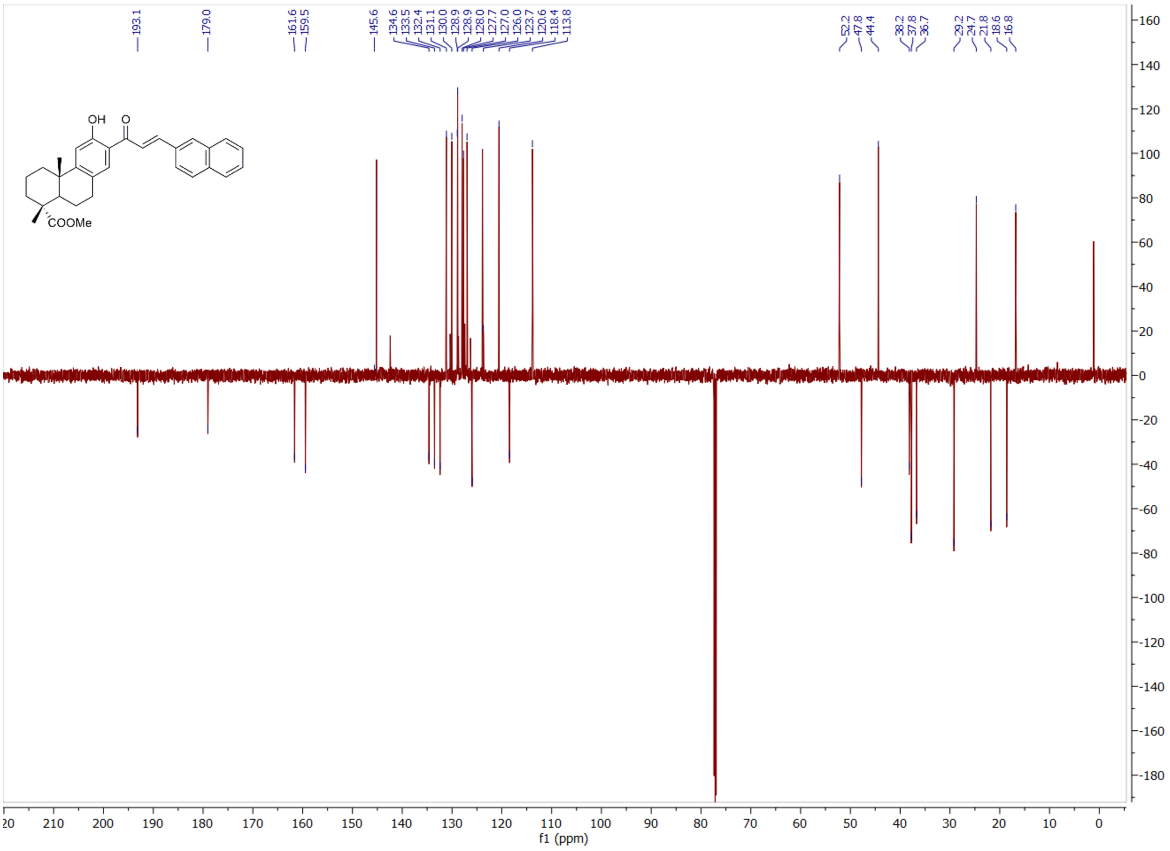

Figure S16. APT-NMR spectrum of compound 37.

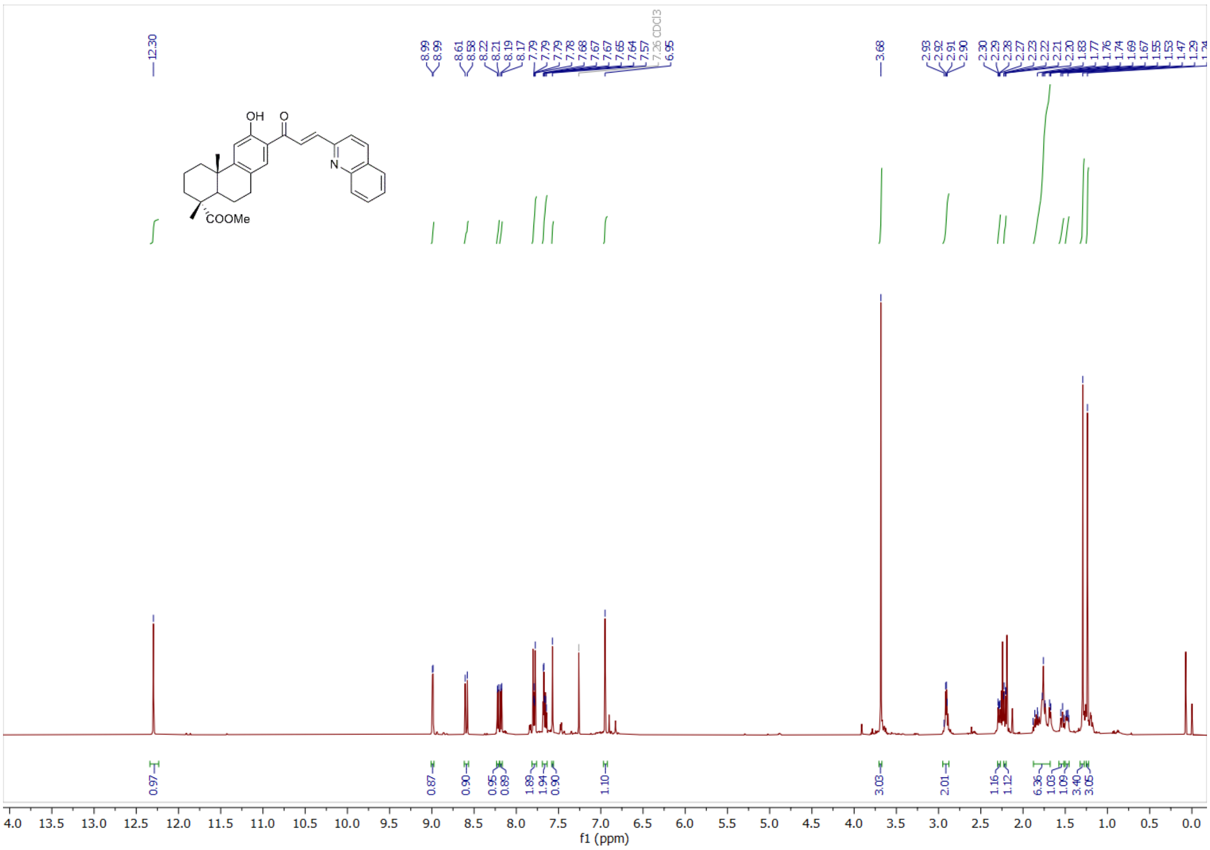

Figure S17. ^{1}H-NMR spectrum of compound 38.

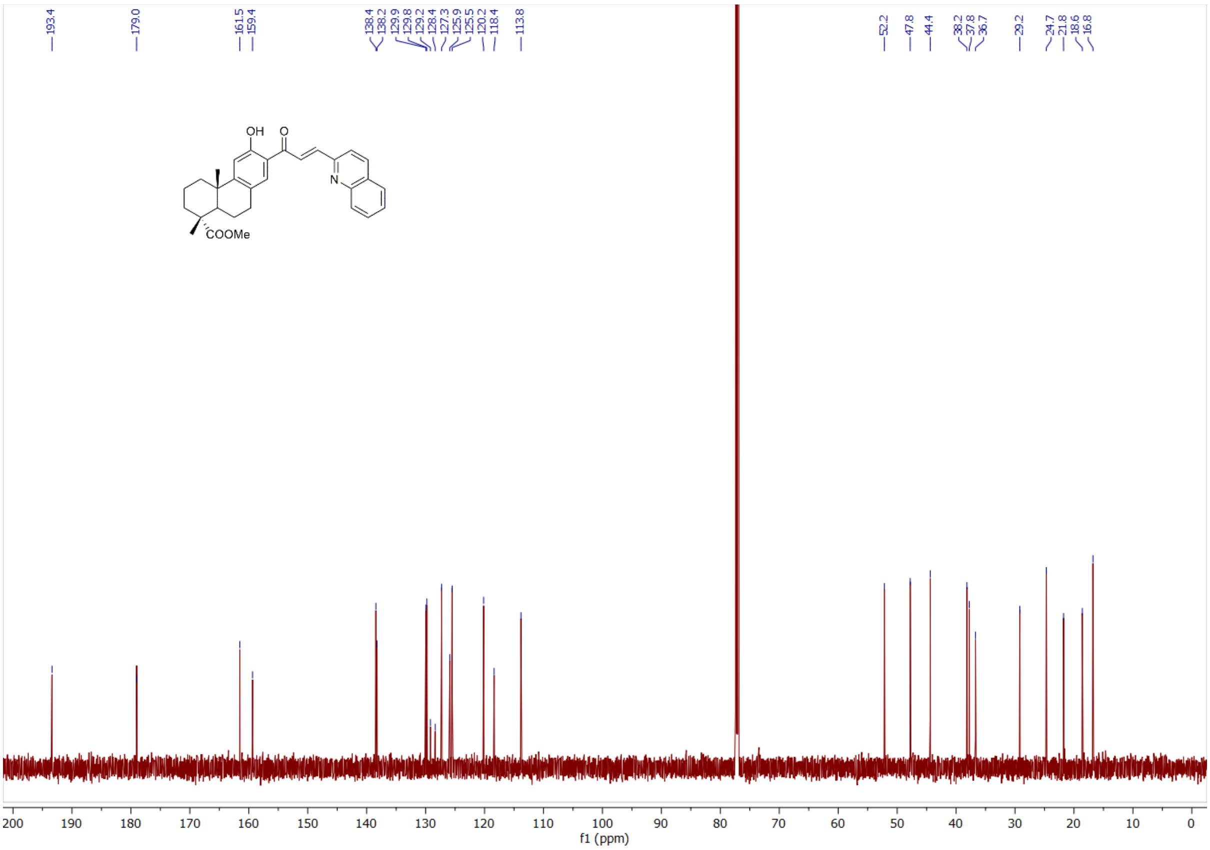

Figure S18. ^{13}C-NMR spectrum of compound 38.

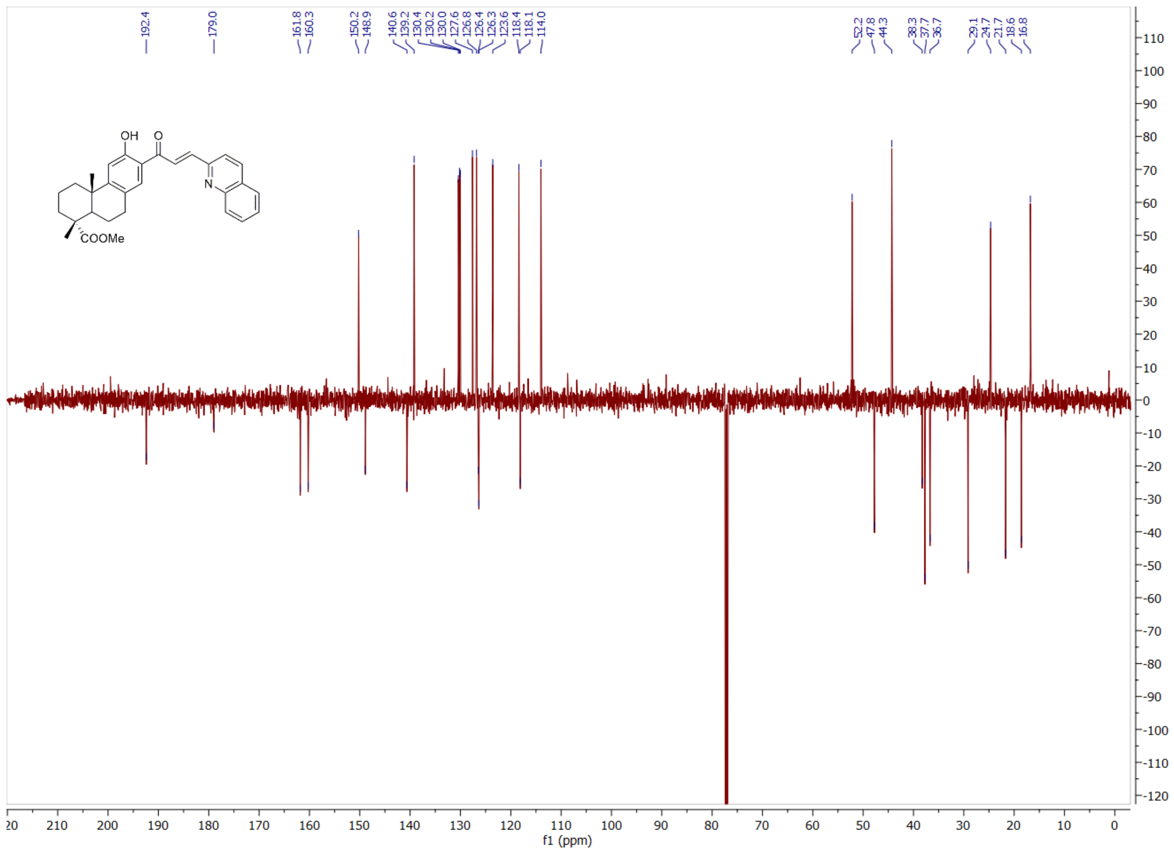

Figure S19. APT-NMR spectrum of compound 38.

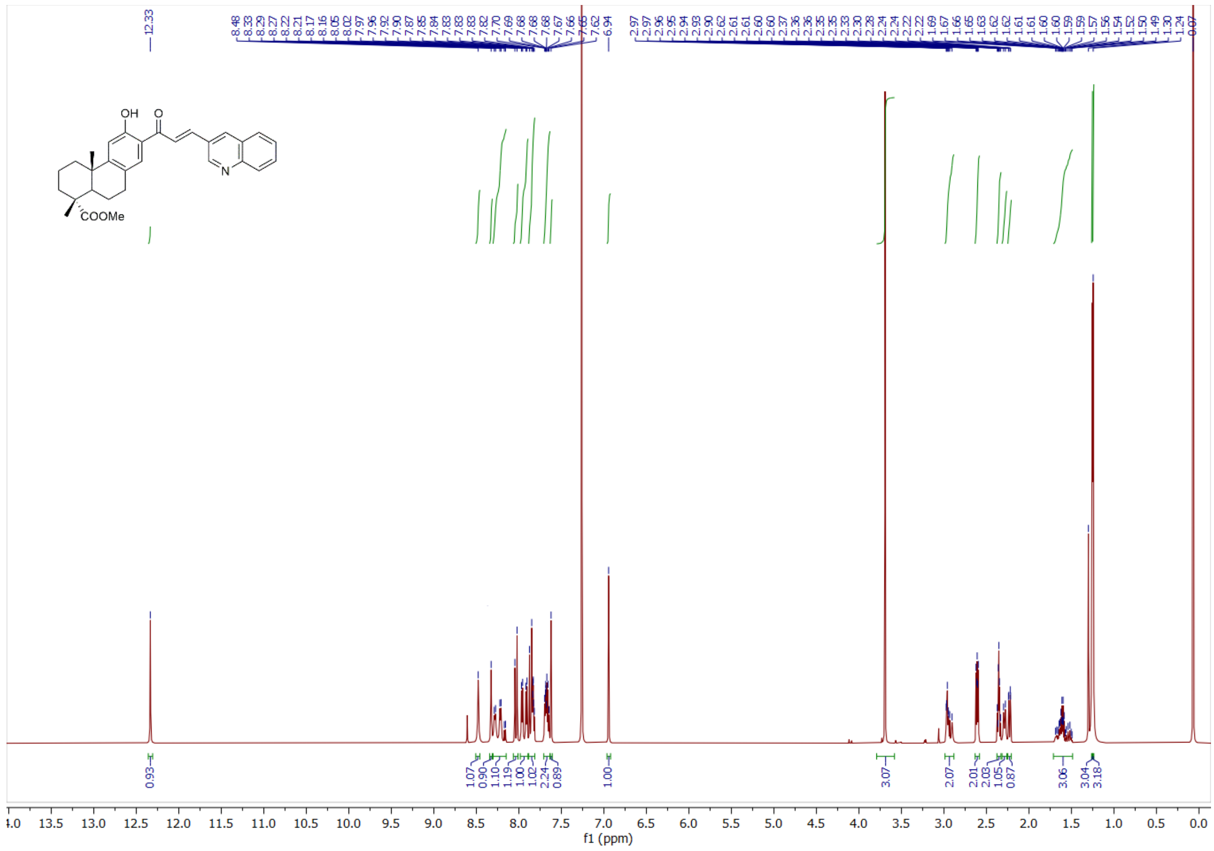

Figure S20. ^{1}H-NMR spectrum of compound 39.

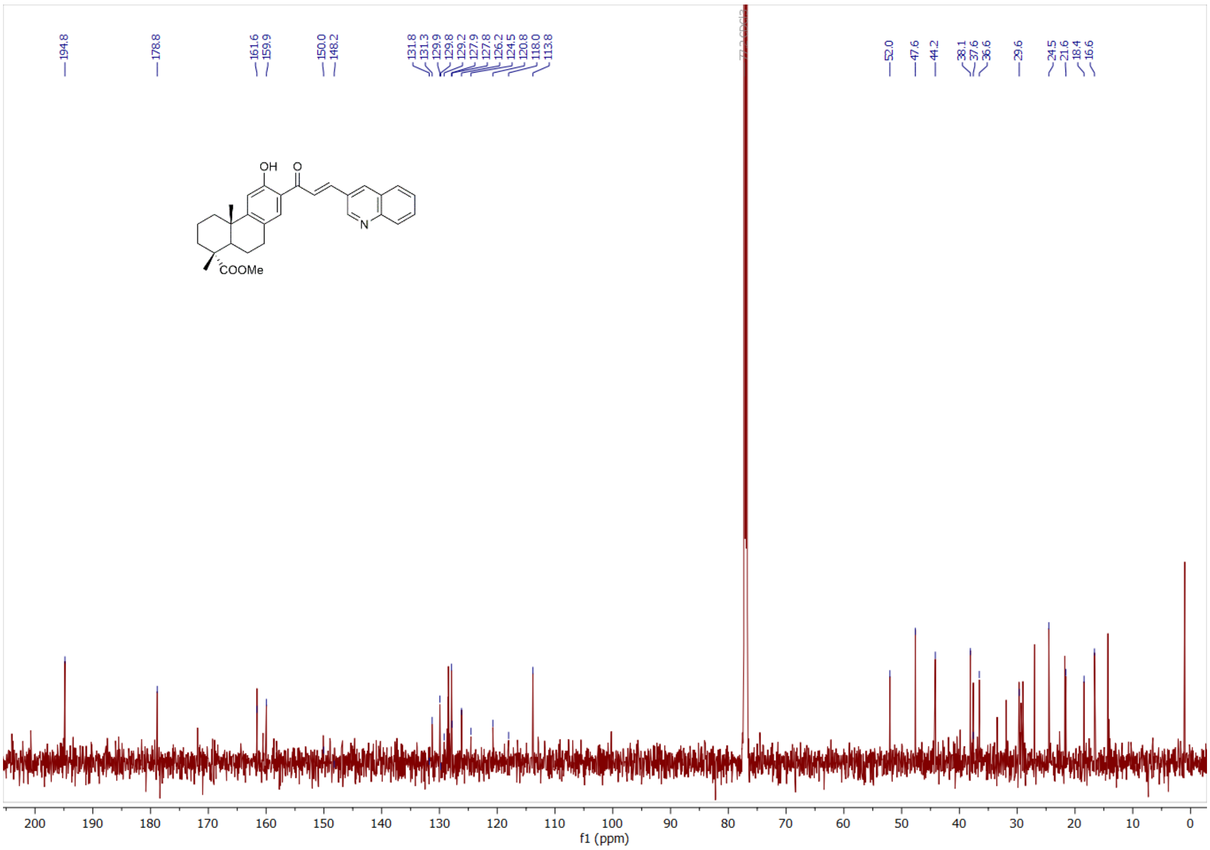

Figure S21. ^{13}C-NMR spectrum of compound 39.

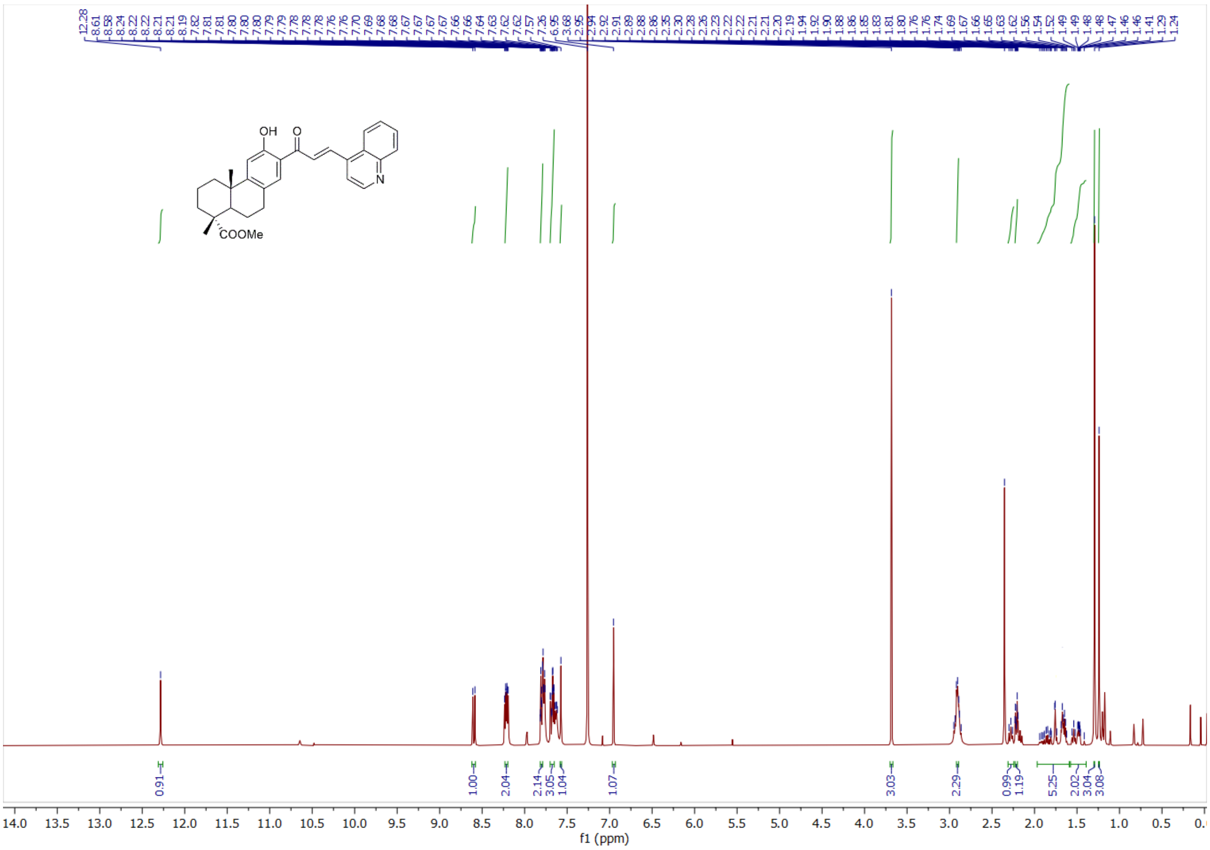

Figure S22. ^{1}H-NMR spectrum of compound 40.

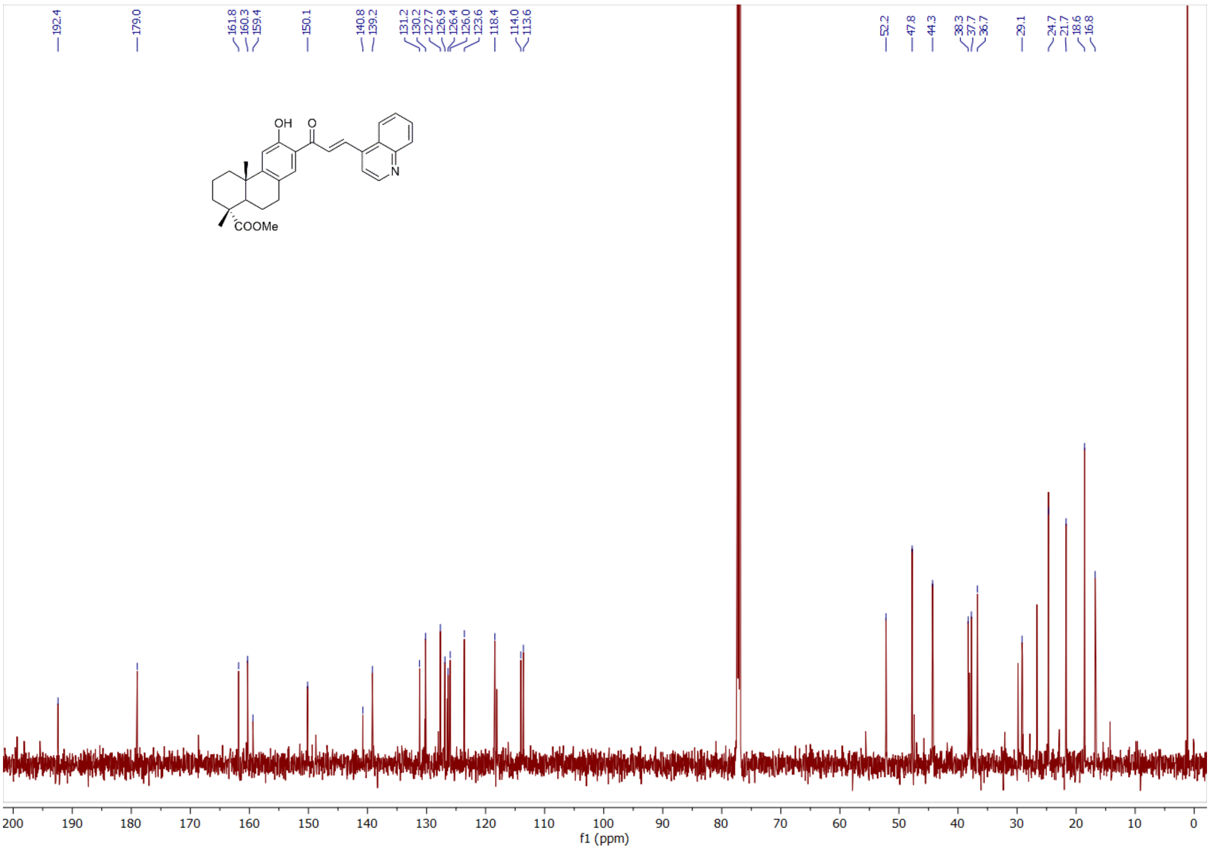

Figure S23. **^{13}C-NMR spectrum of compound 40.**

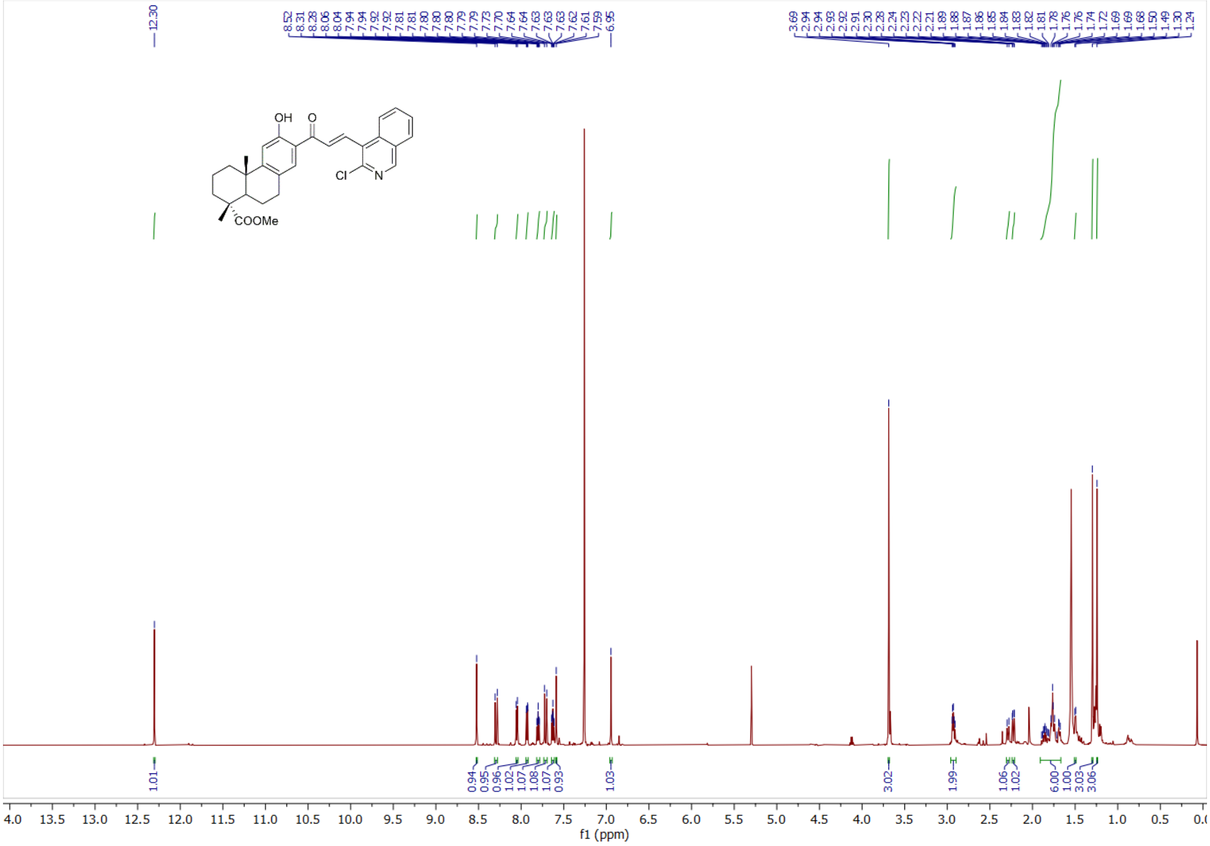

Figure S24. **^{1}H-NMR spectrum of compound 41.**

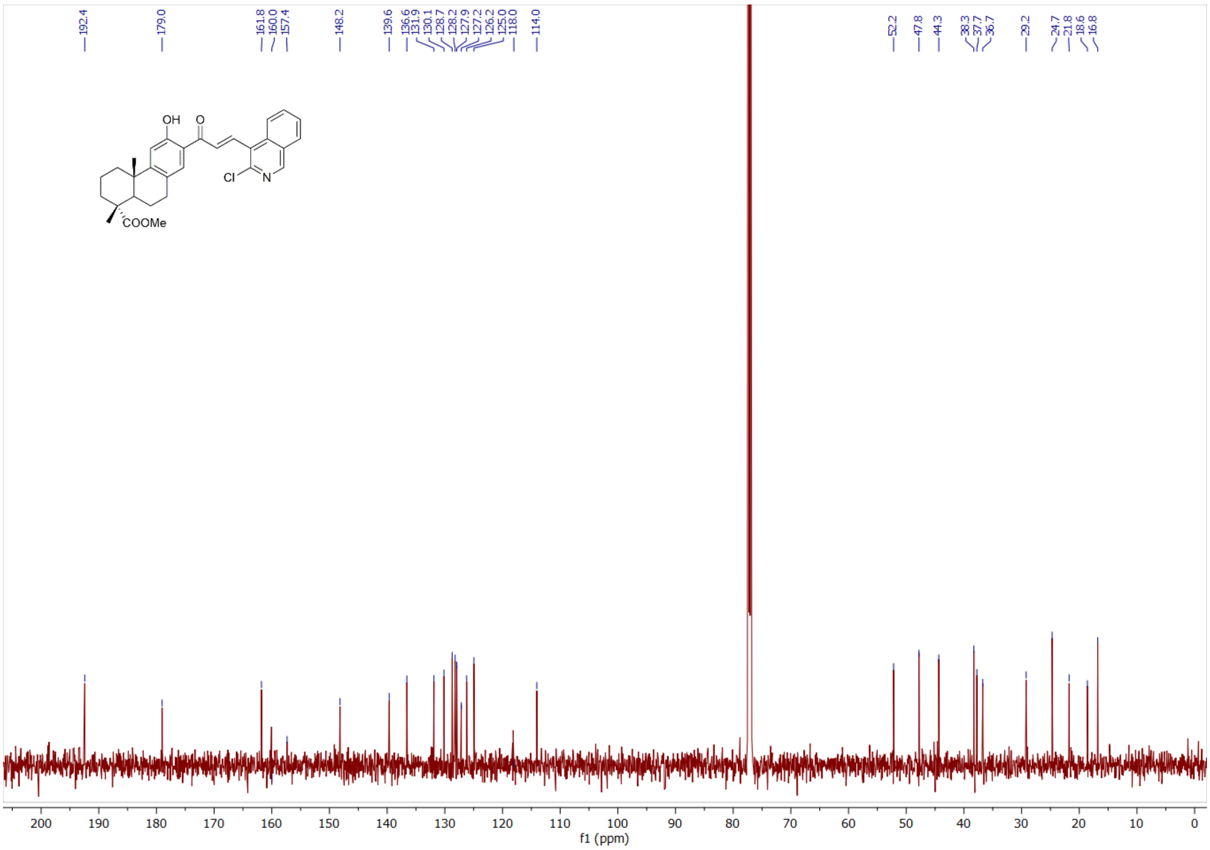

Figure S25. ^{13}C-NMR spectrum of compound 41.

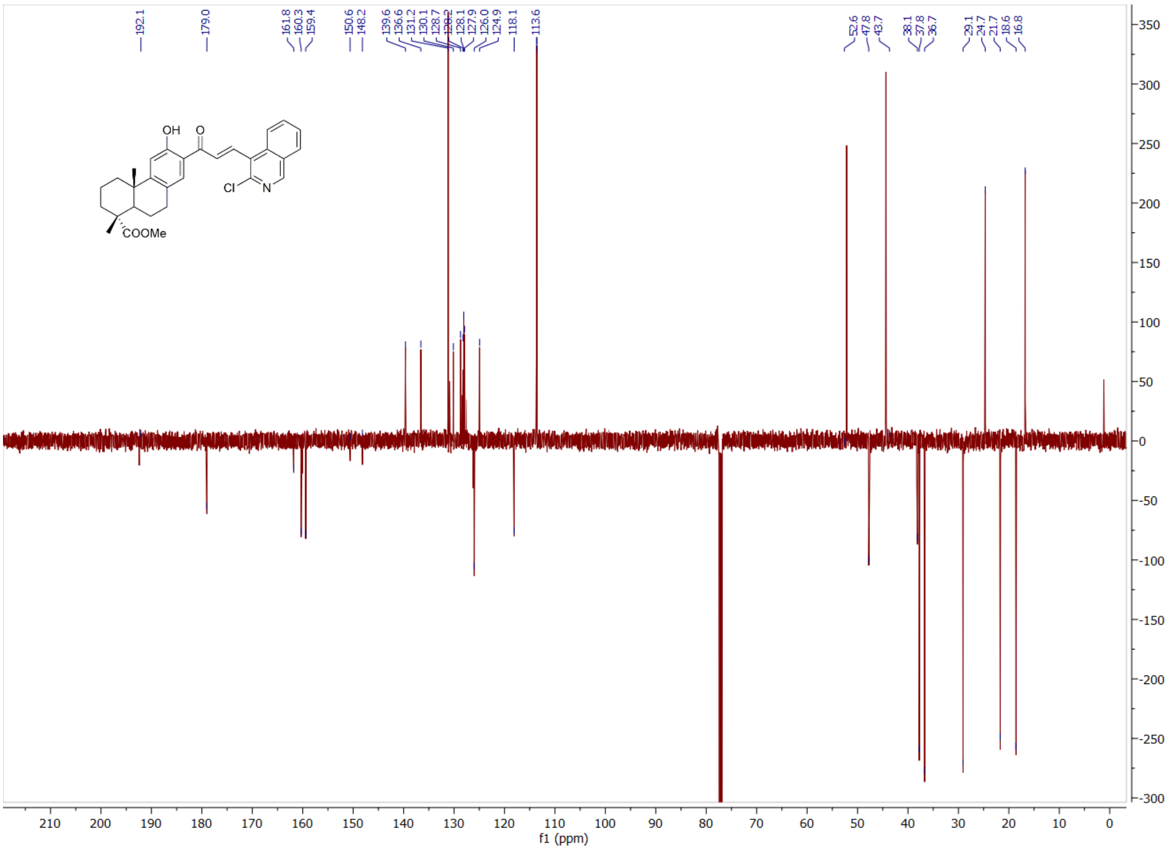

Figure S26. APT-NMR spectrum of compound 41.

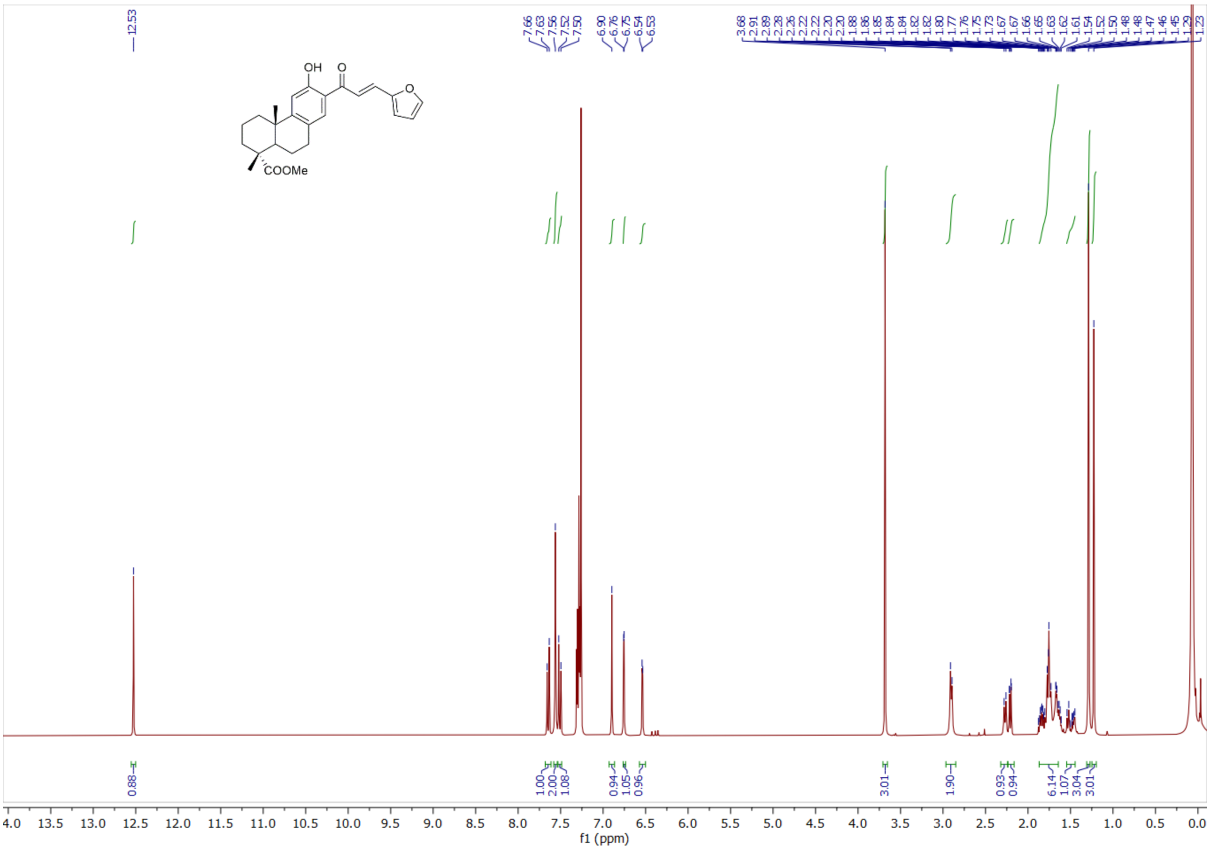

Figure S27. ^{1}H-NMR spectrum of compound 42.

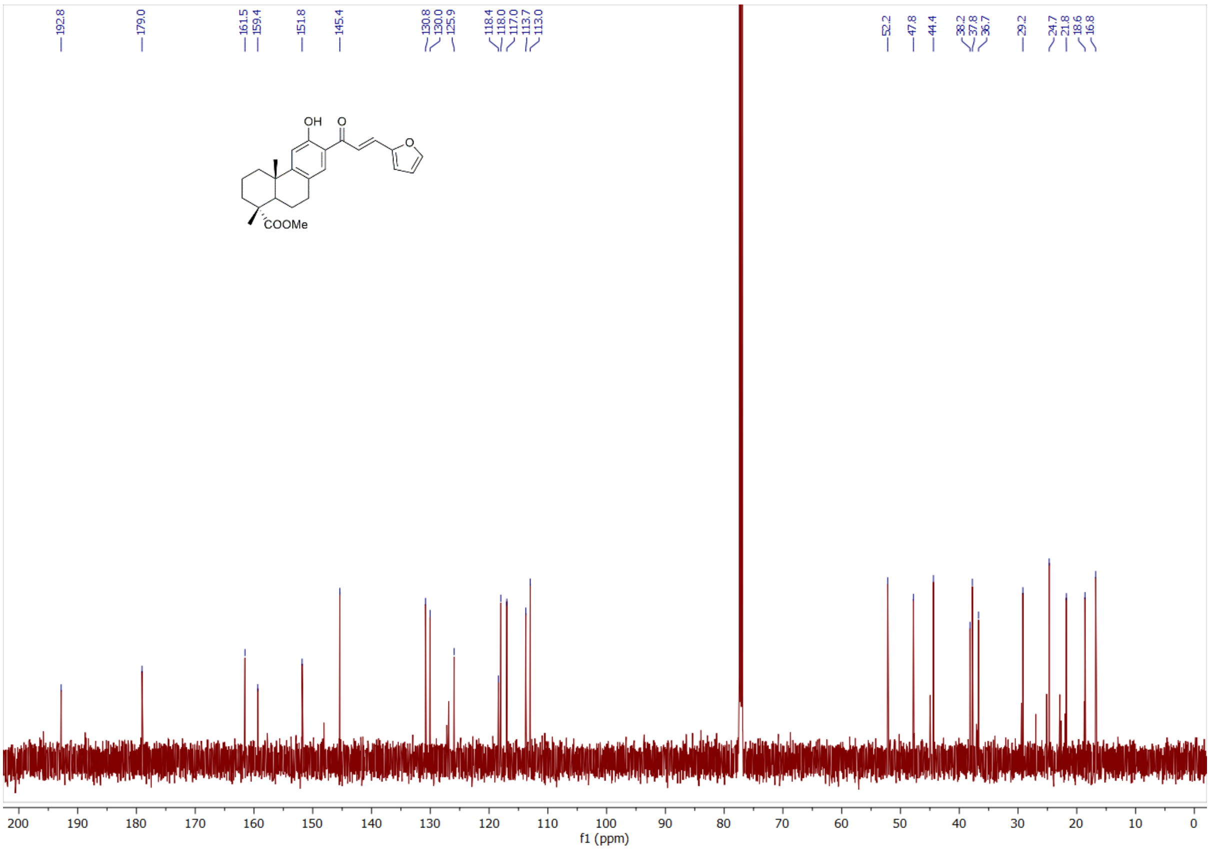

Figure S28. ^{13}C-NMR spectrum of compound 42.

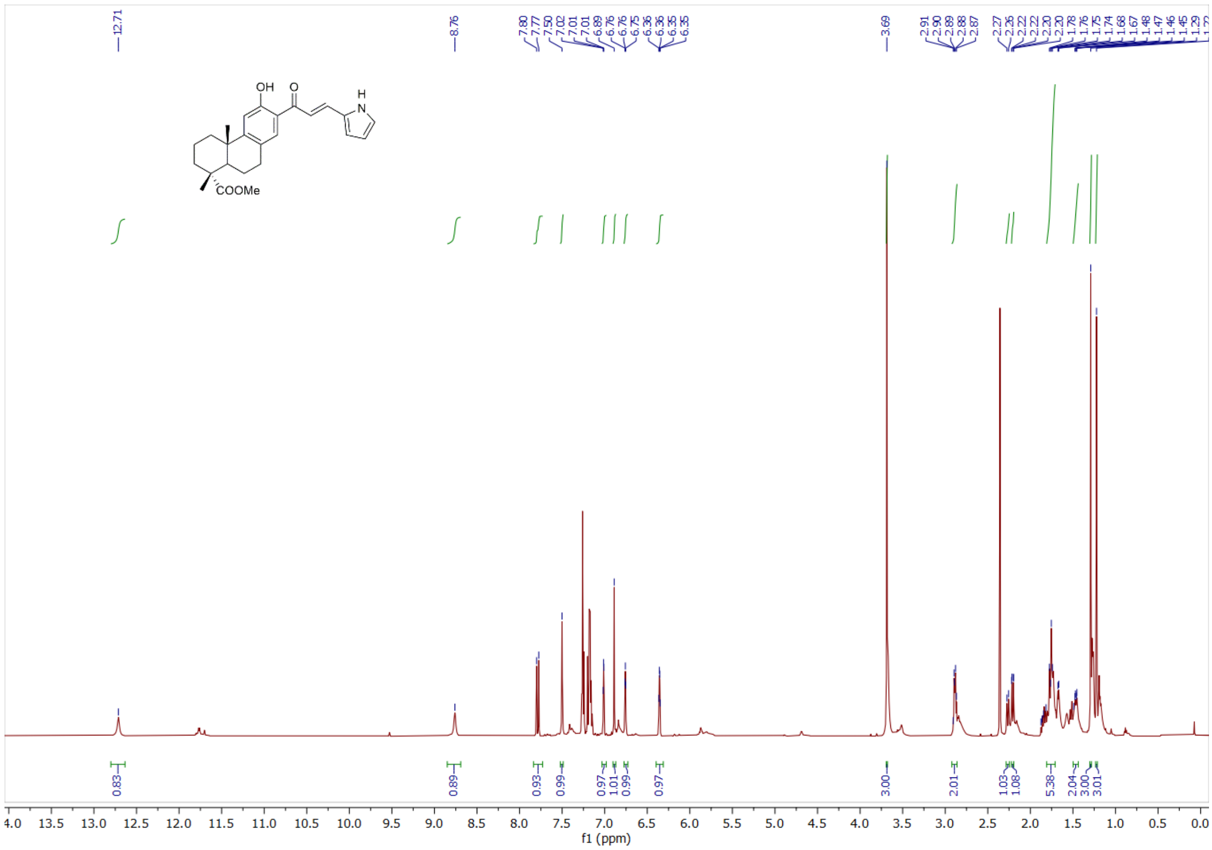

Figure S29. ^{1}H-NMR spectrum of compound 43.

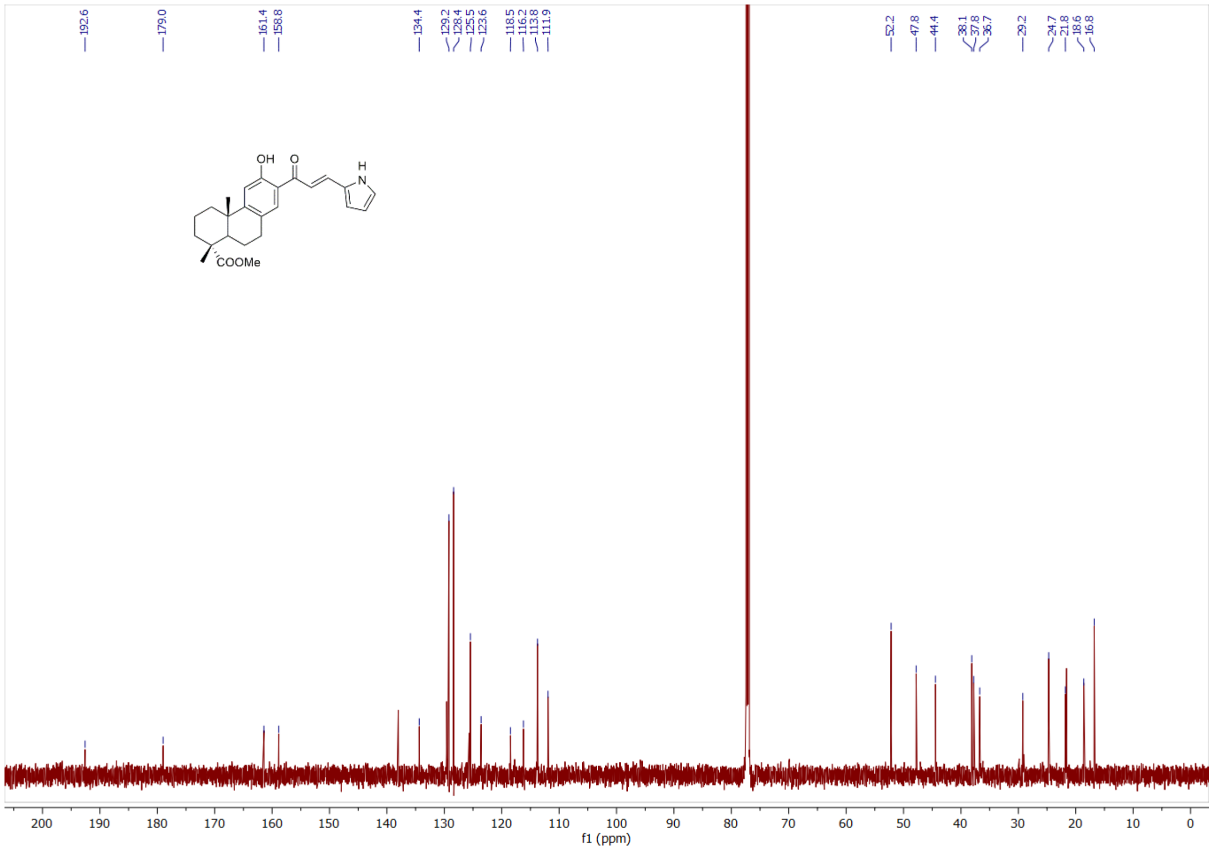

Figure S30. ^{13}C-NMR spectrum of compound 43.

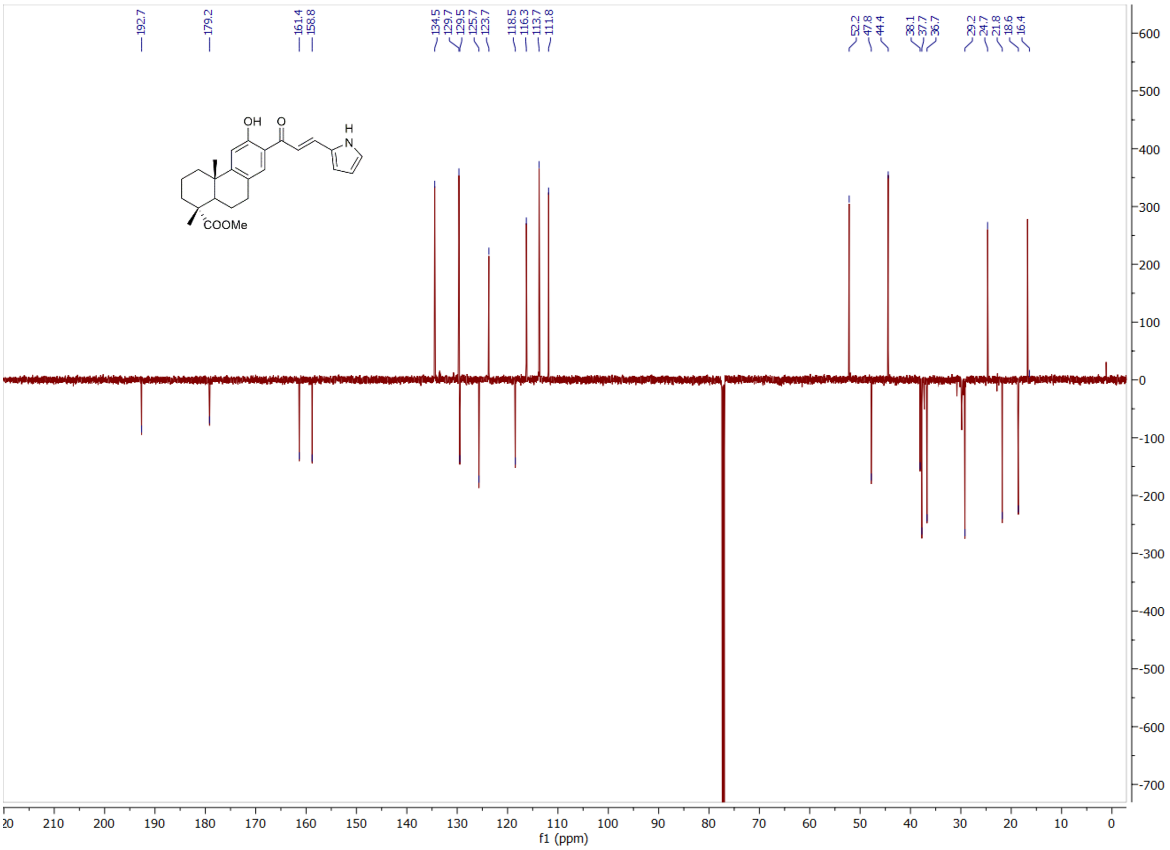

Figure S31. APT-NMR spectrum of compound 43.

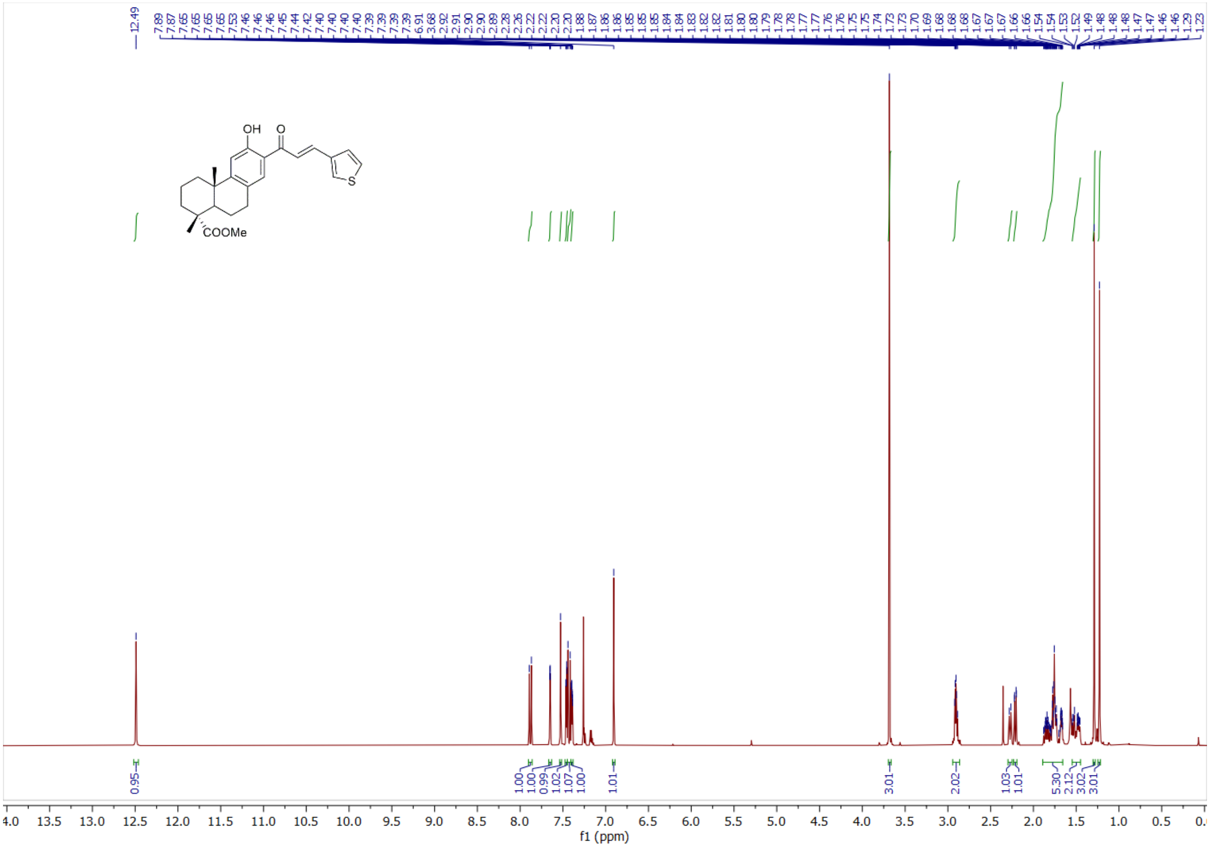

Figure S32. ¹H-NMR spectrum of compound 44.

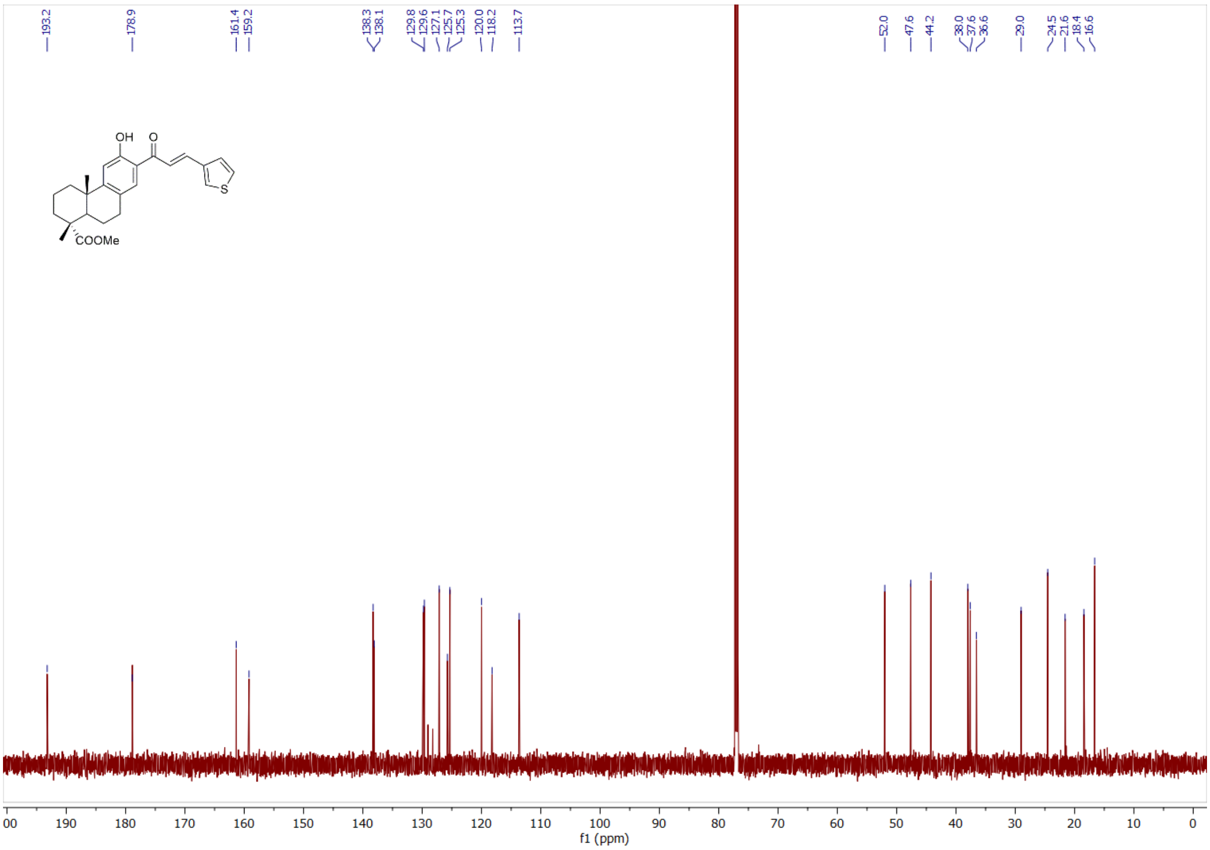

Figure S33. ^{13}**C-NMR spectrum of compound 44.**

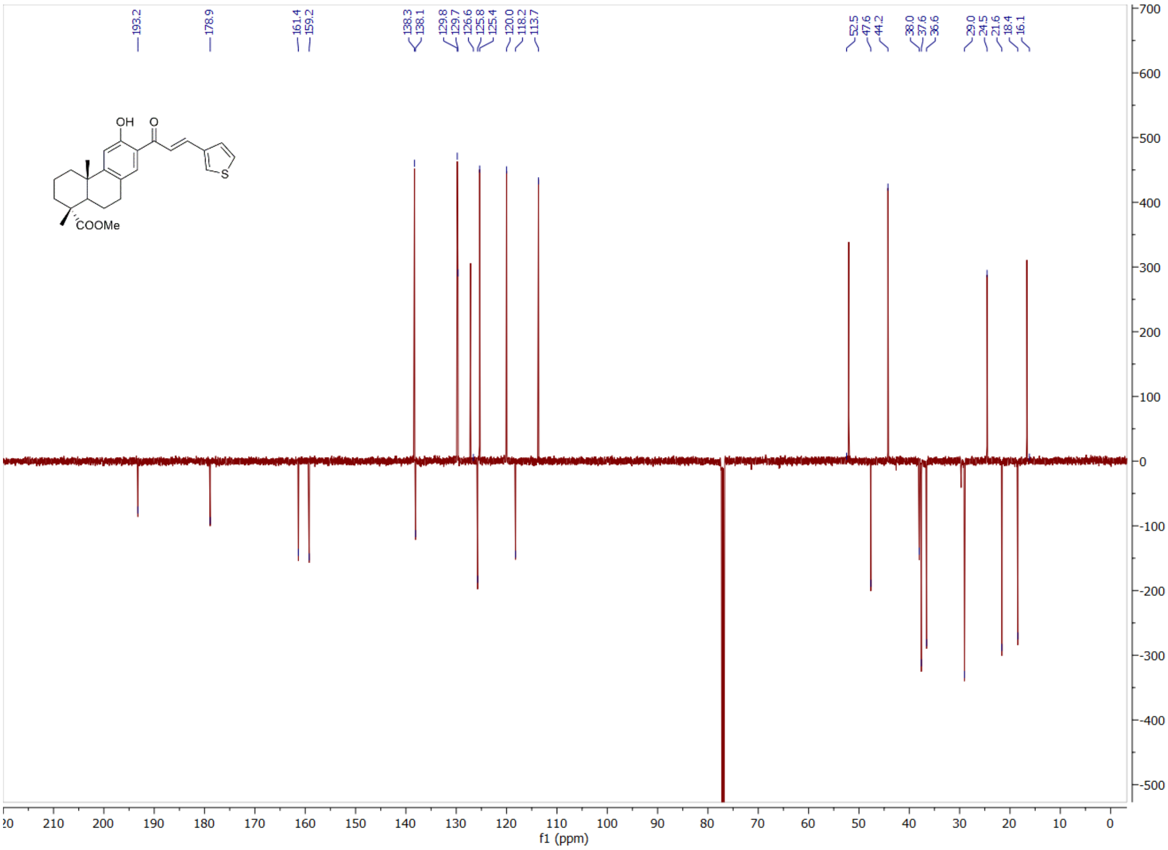

Figure S34. APT-NMR spectrum of compound 44.

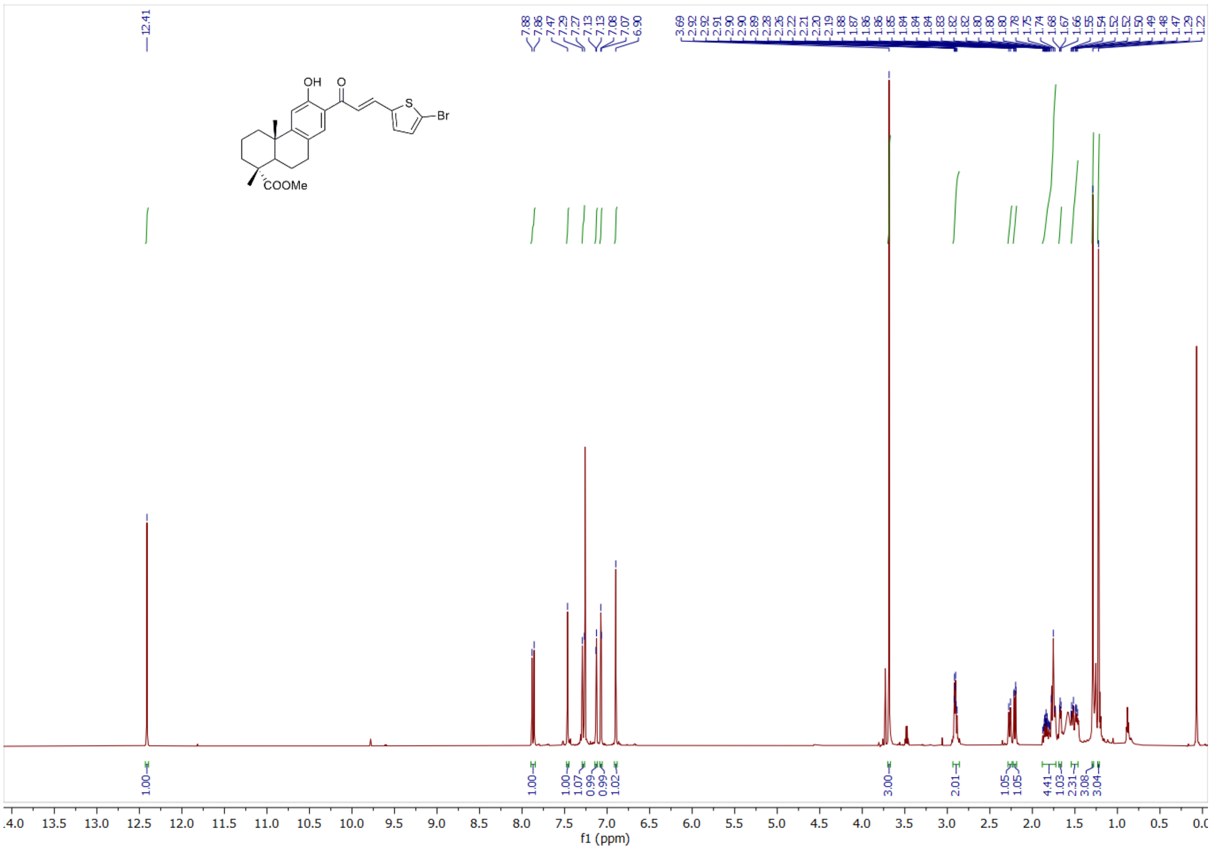

Figure S35. ^{1}H-NMR spectrum of compound 45.

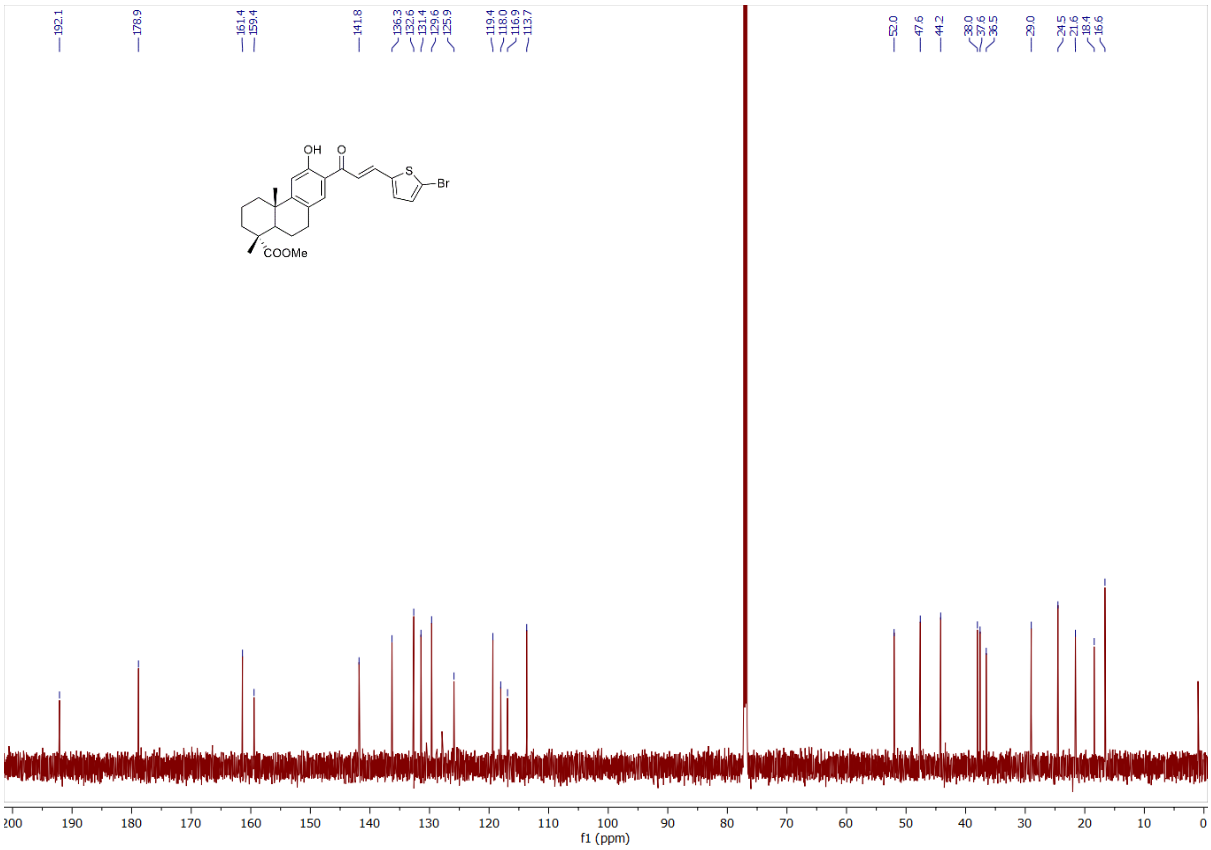

Figure S36. ^{13}C-NMR spectrum of compound 45.

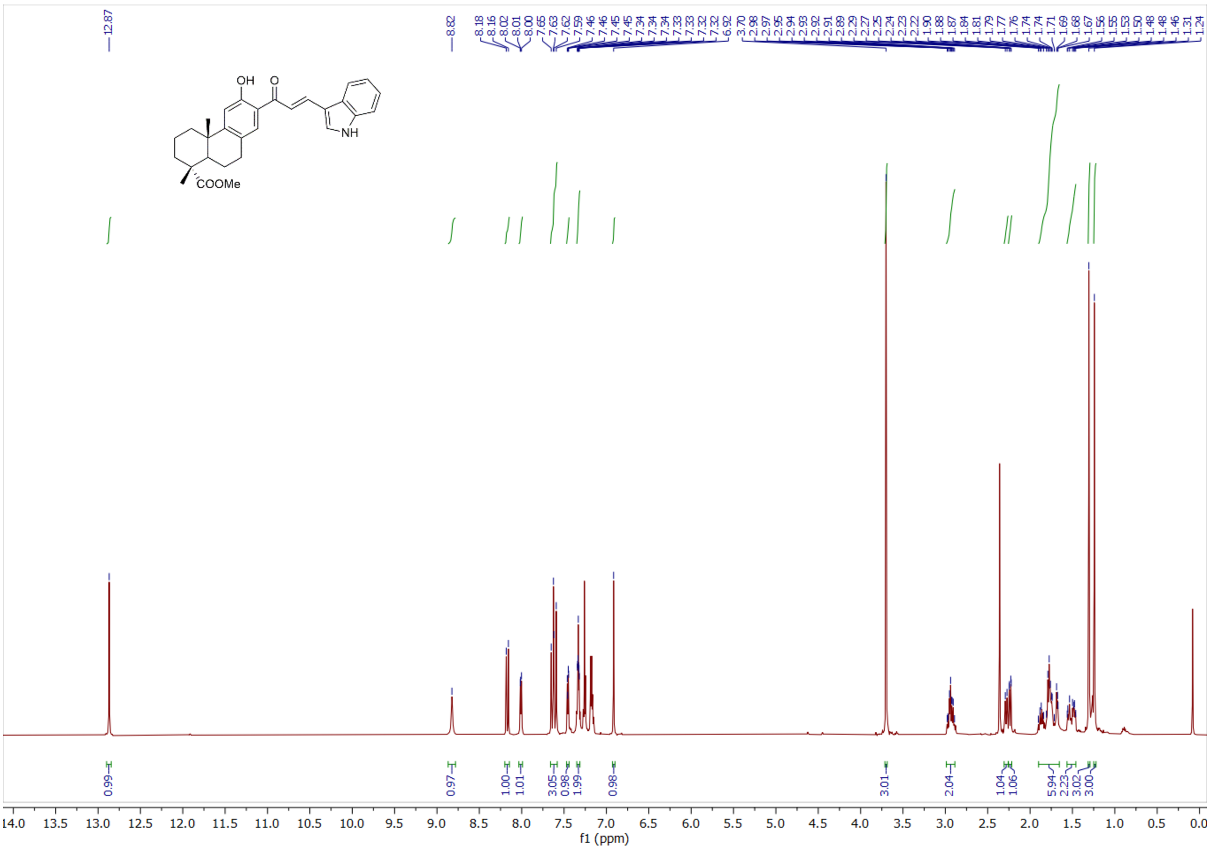

Figure S37. ^{1}H-NMR spectrum of compound 46.

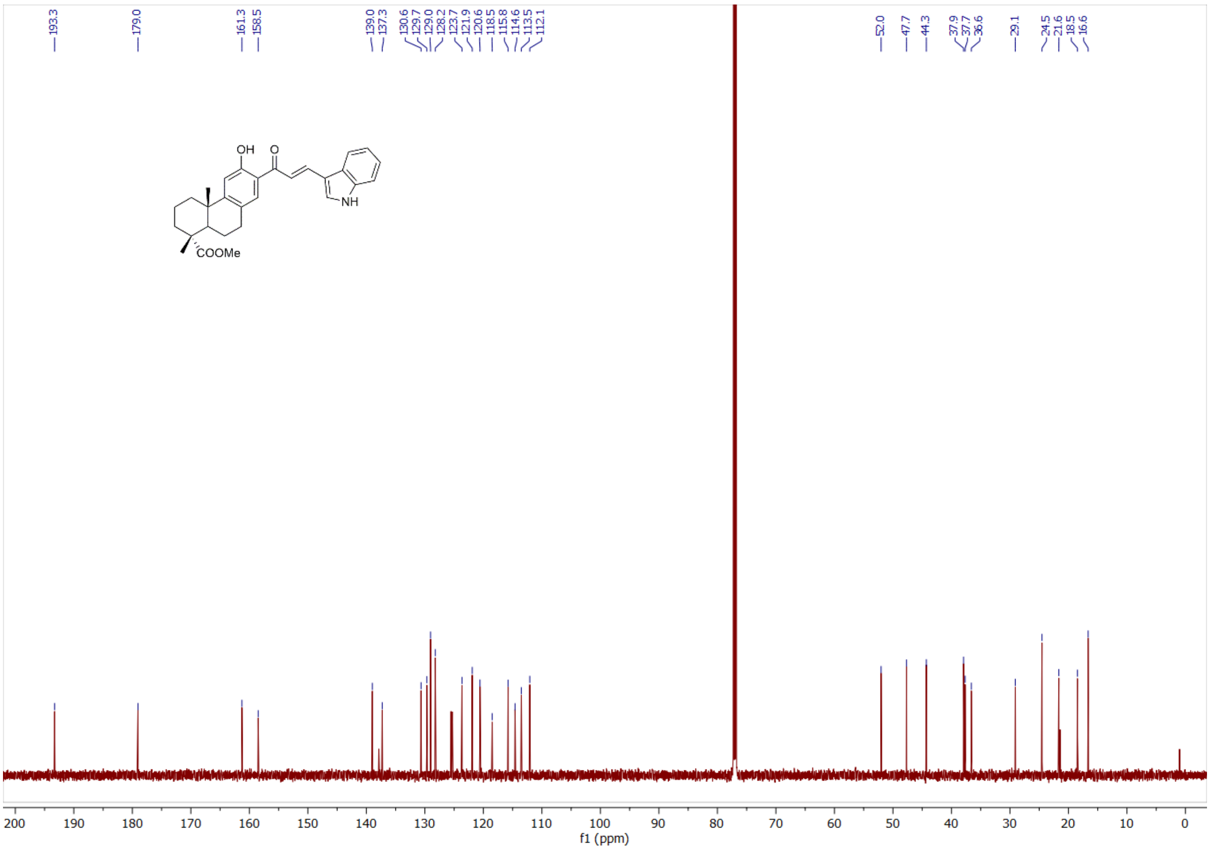

Figure S38. ^{13}C-NMR spectrum of compound 46.

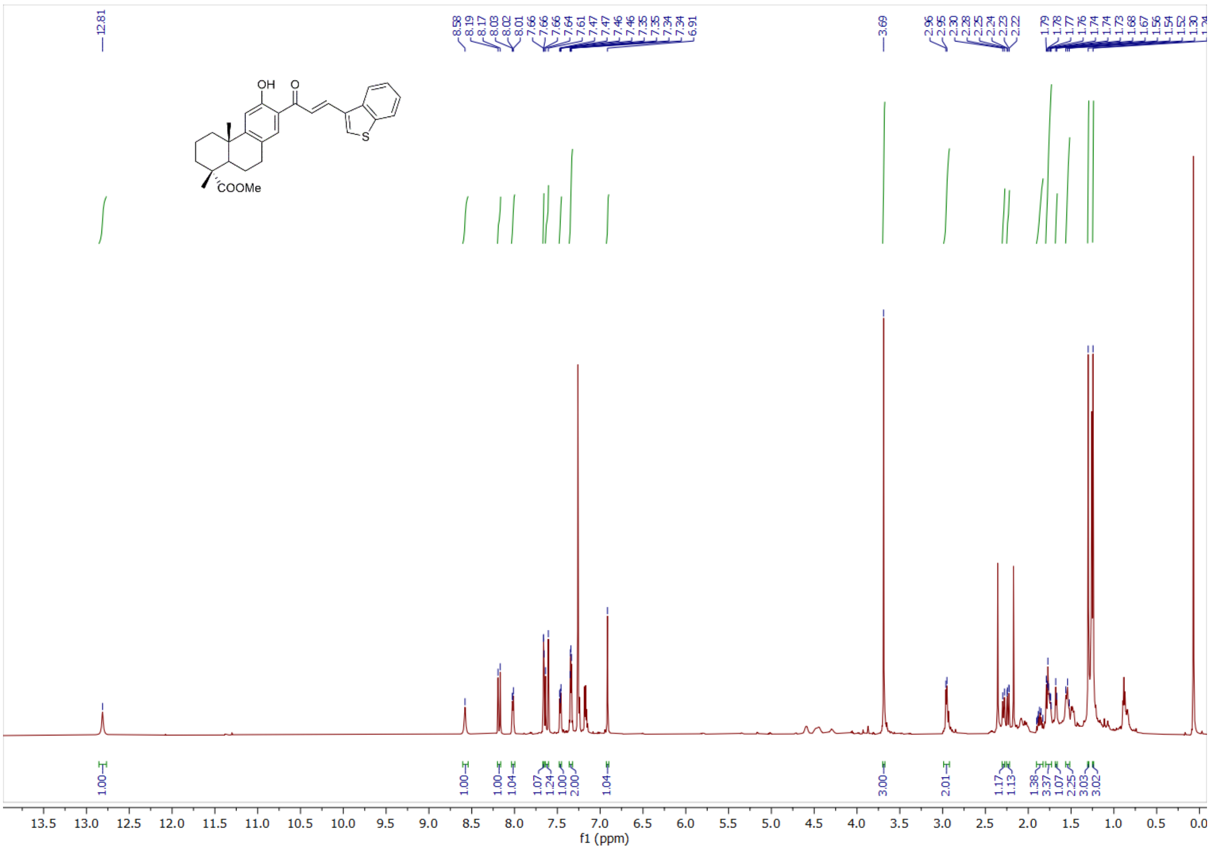

Figure S39. **^{1}H-NMR spectrum of compound 47.**

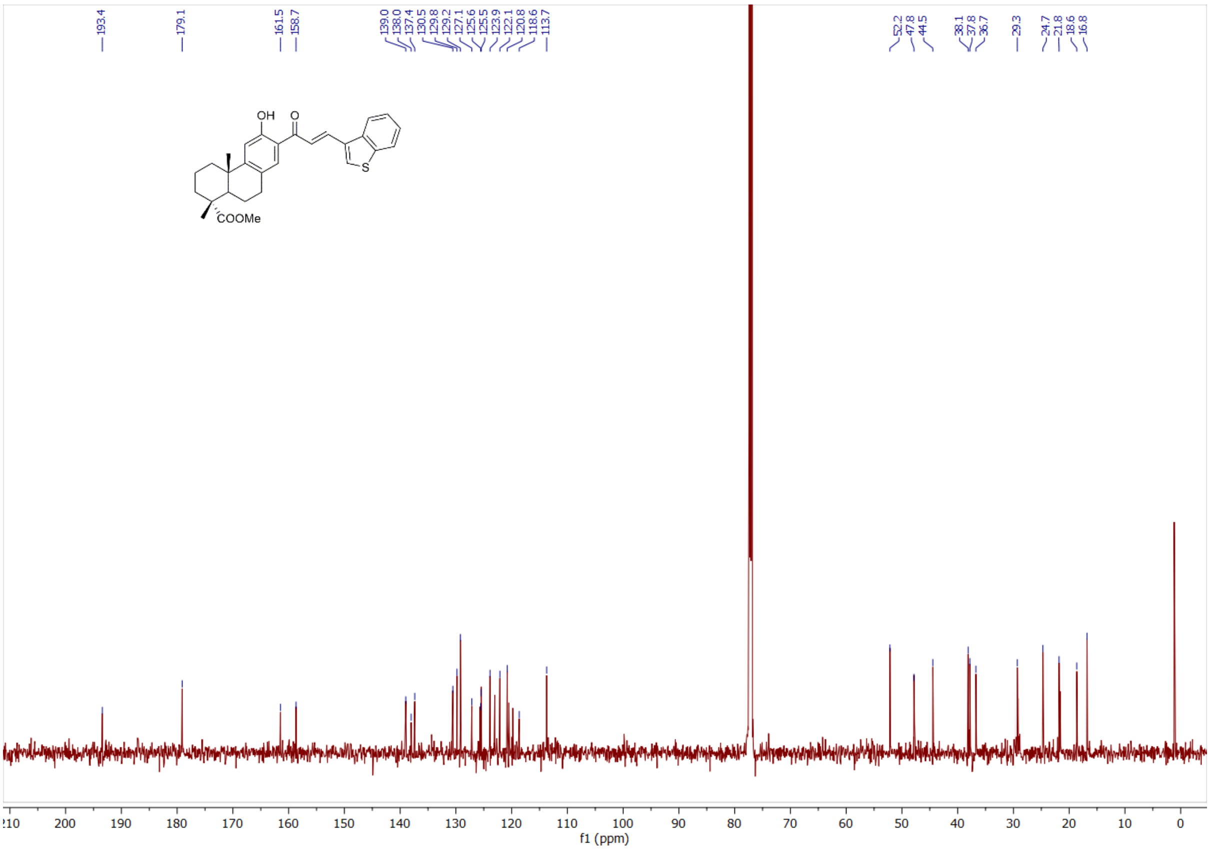

Figure S40. **^{13}C-NMR spectrum of compound 47.**

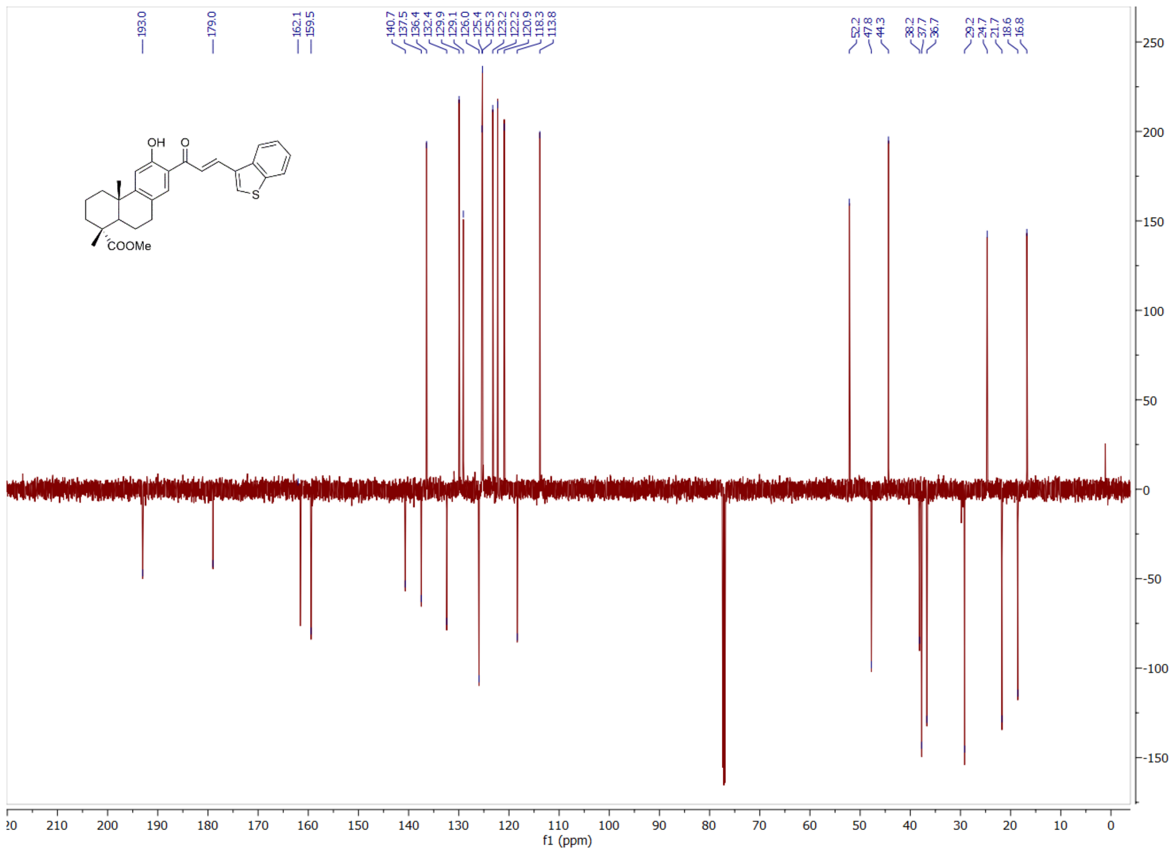

Figure S40. APT-NMR spectrum of compound 47.

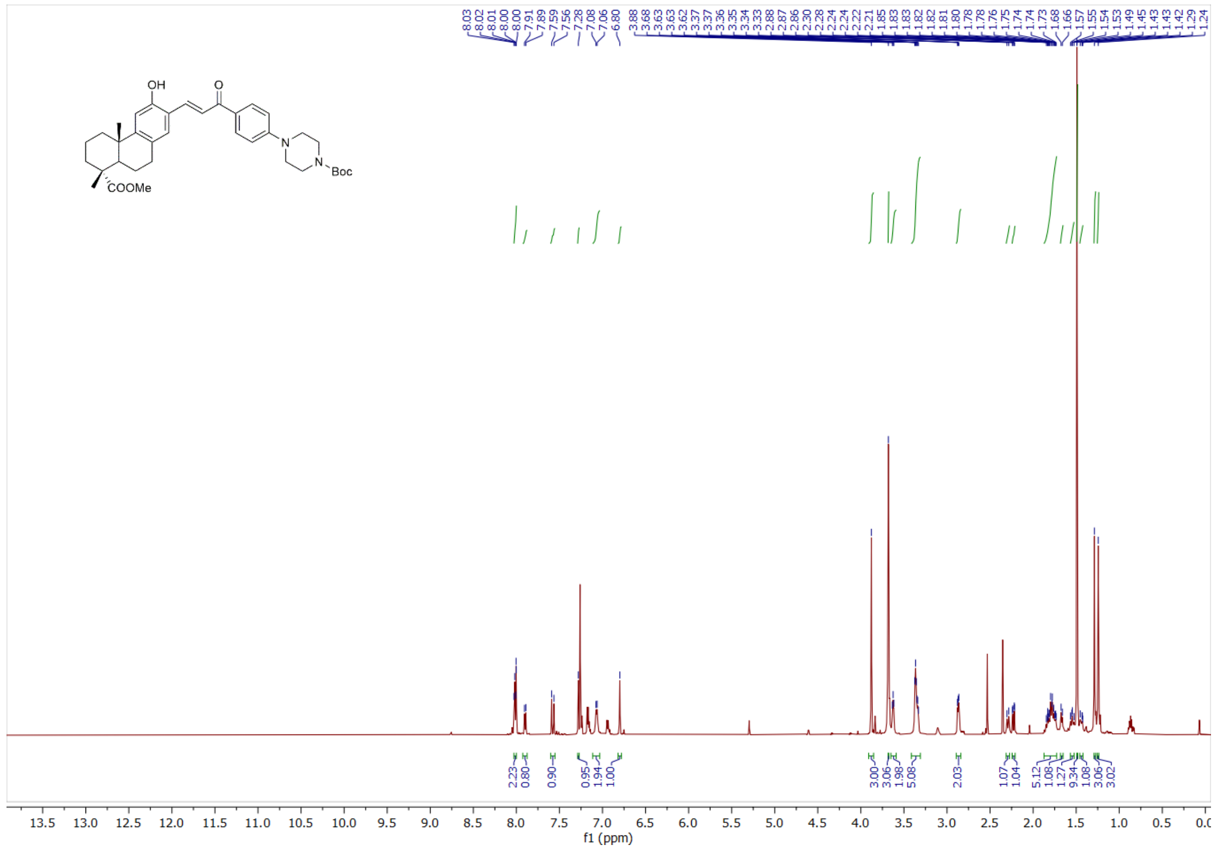

Figure S42. ^{1}H-NMR spectrum of compound 48.

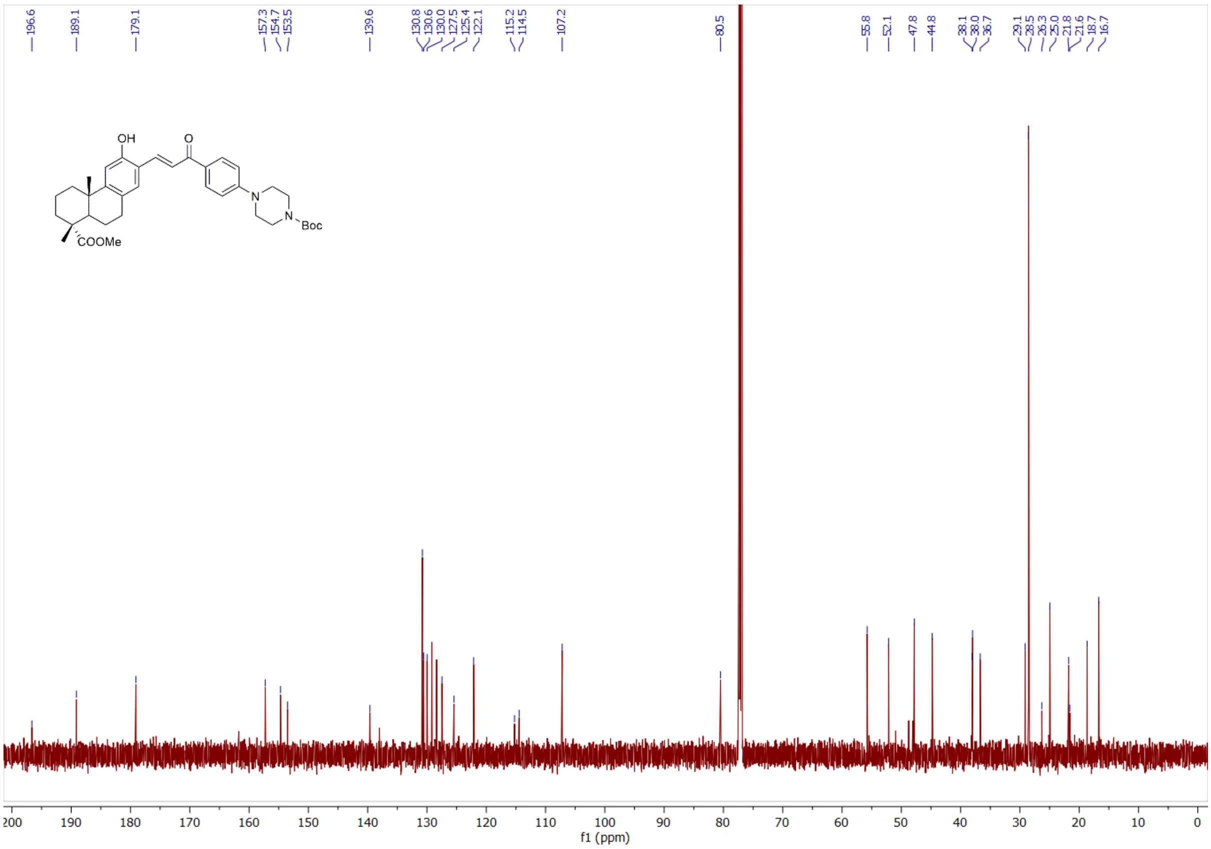

Figure S43. ^{13}**C-NMR spectrum of compound 48.**

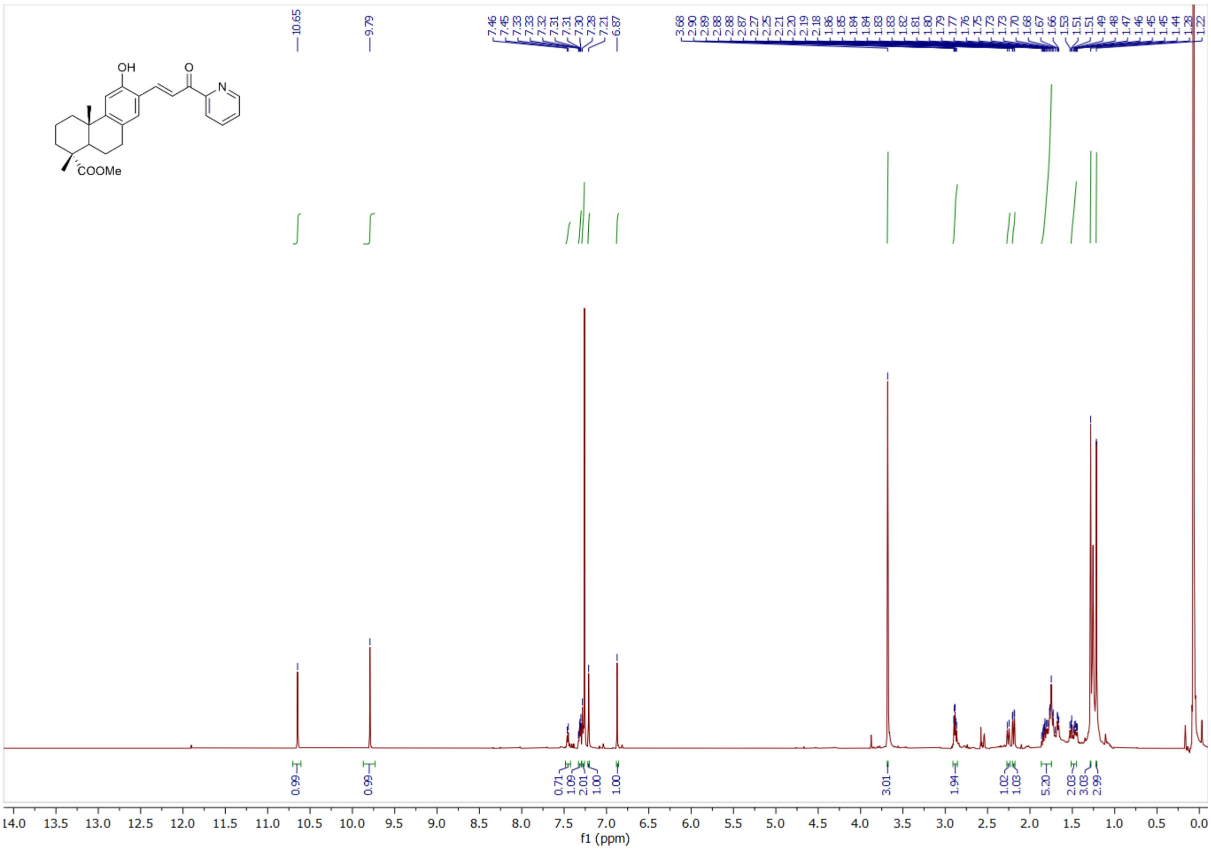

Figure S44. ^{1}**H-NMR spectrum of compound 49.**

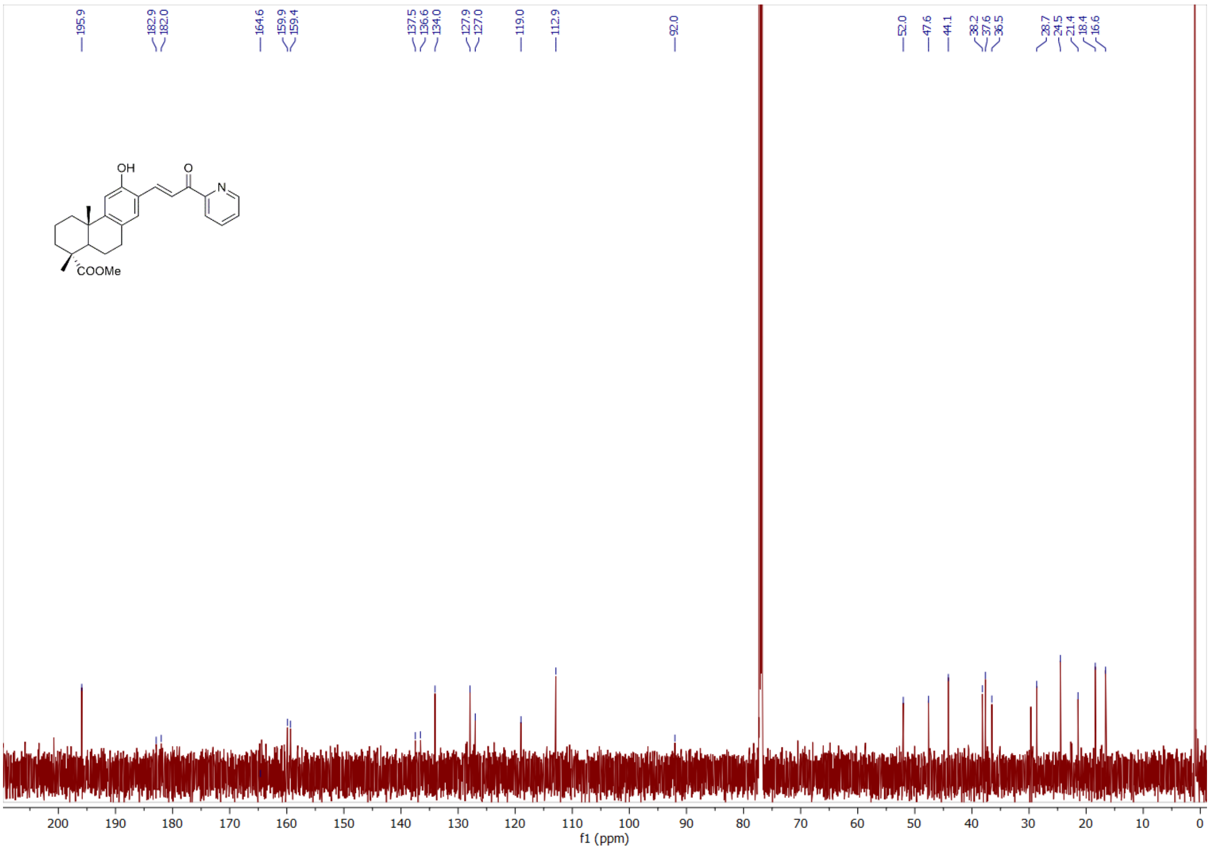

Figure S45. ^{13}C-NMR spectrum of compound 49.

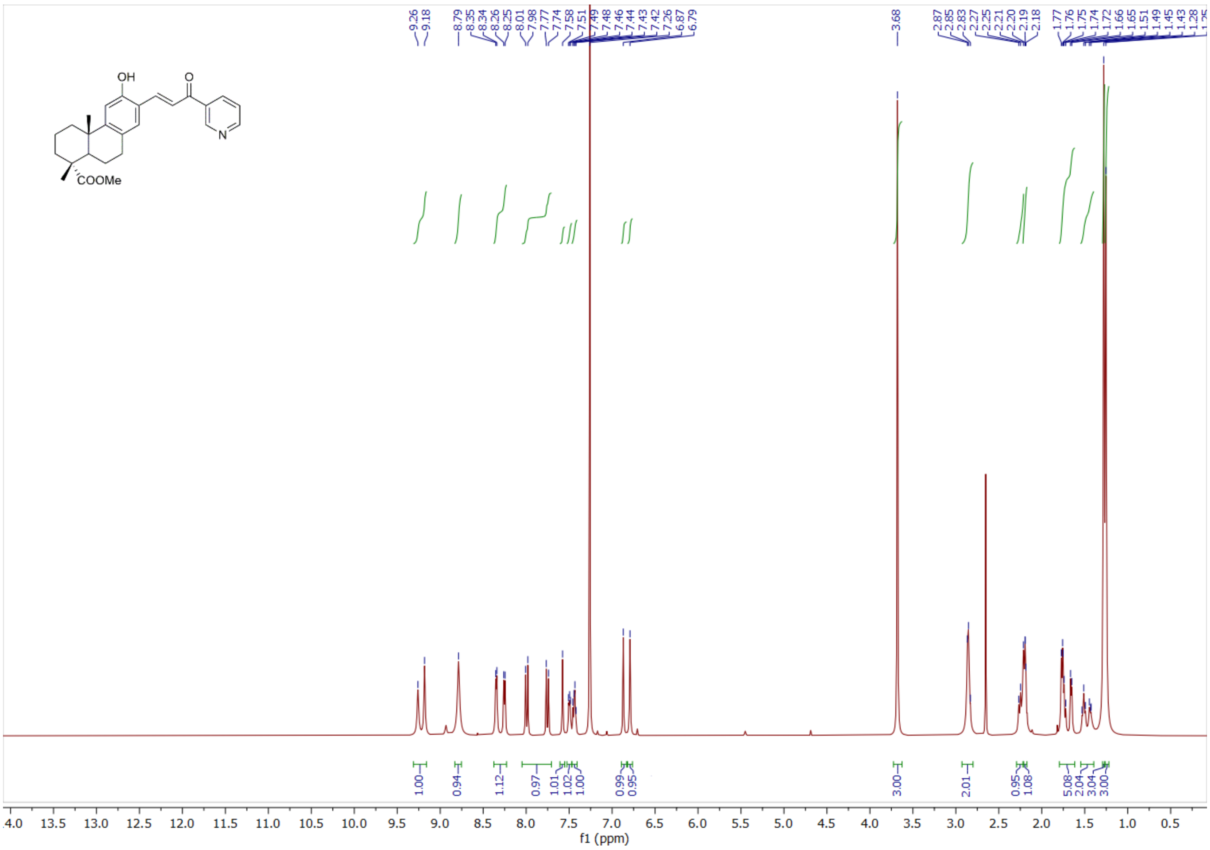

Figure S46. ^{1}H-NMR spectrum of compound 50.

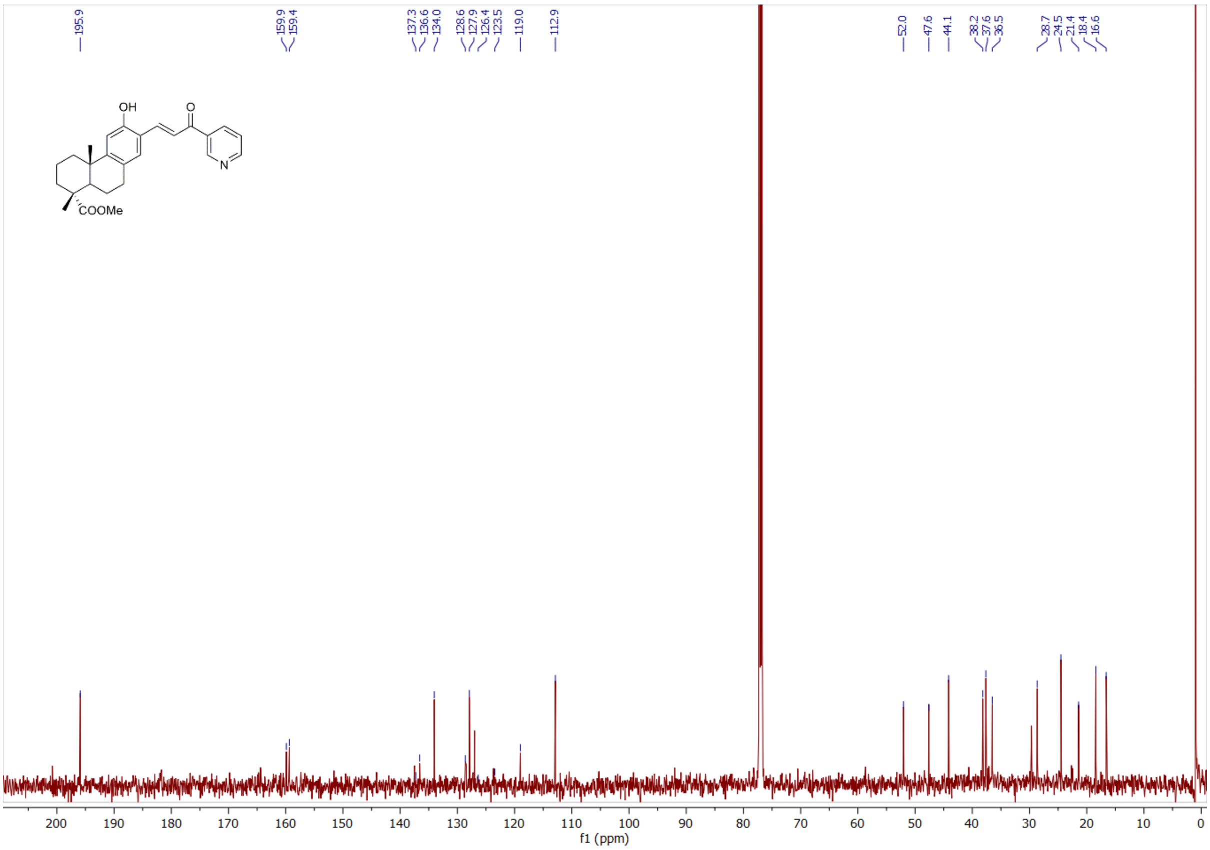

Figure S47. ^{13}**C-NMR spectrum of compound 50.**

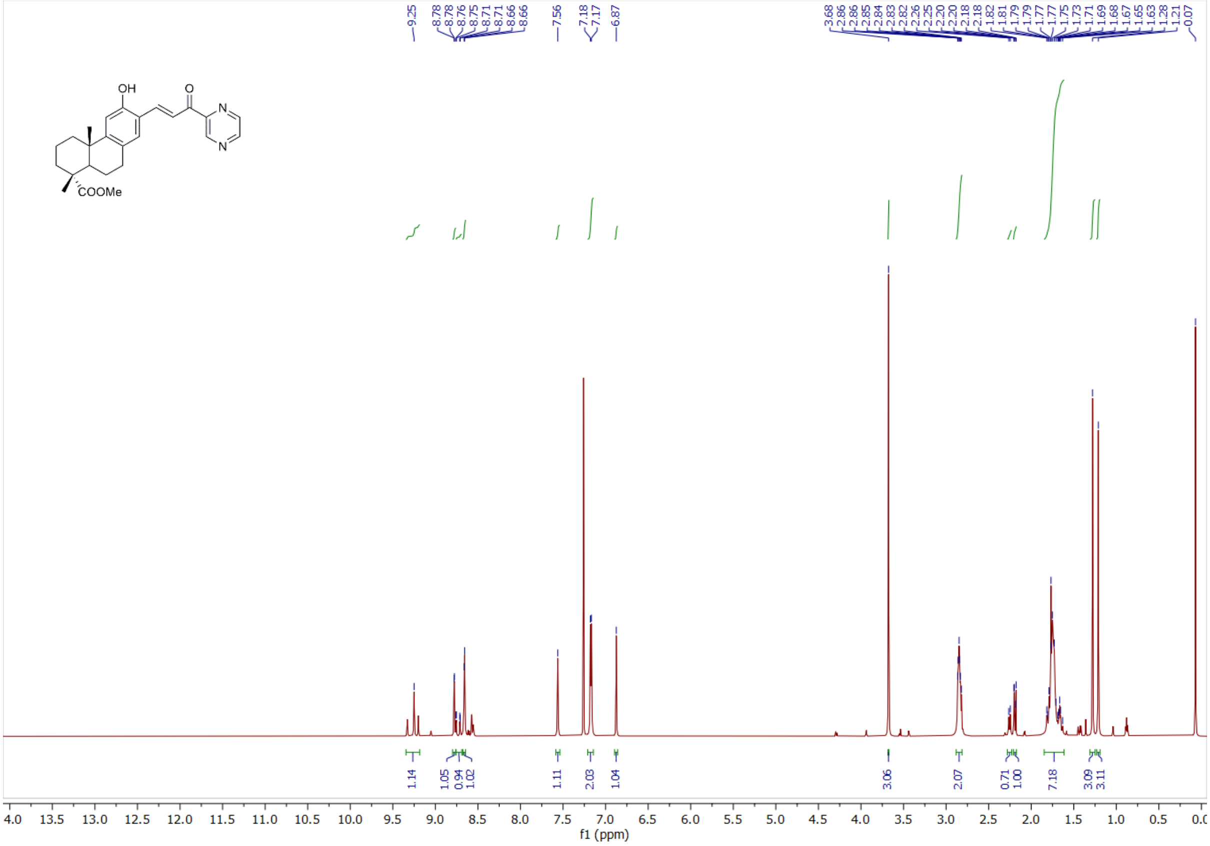

Figure S48. ^{1}**H-NMR spectrum of compound 51.**

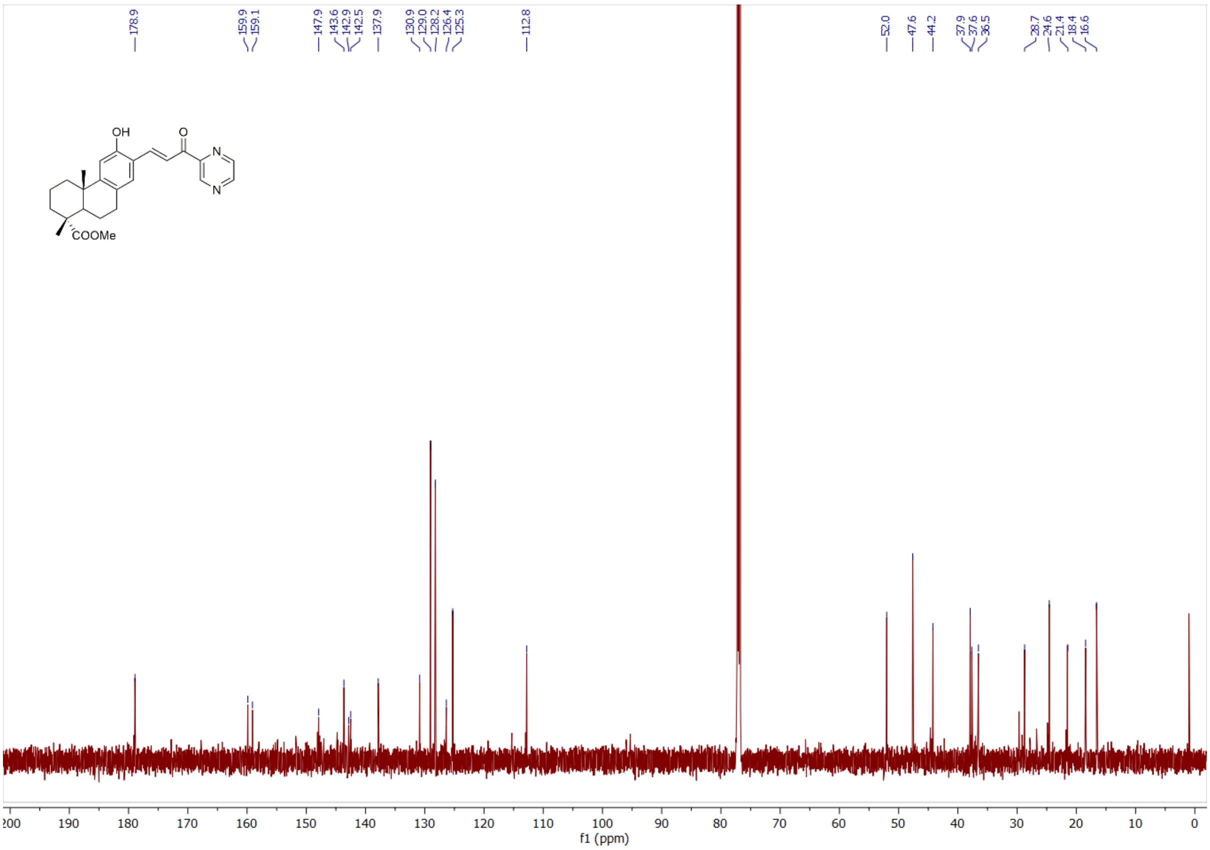

Figure S49. ^{13}C-NMR spectrum of compound 51.

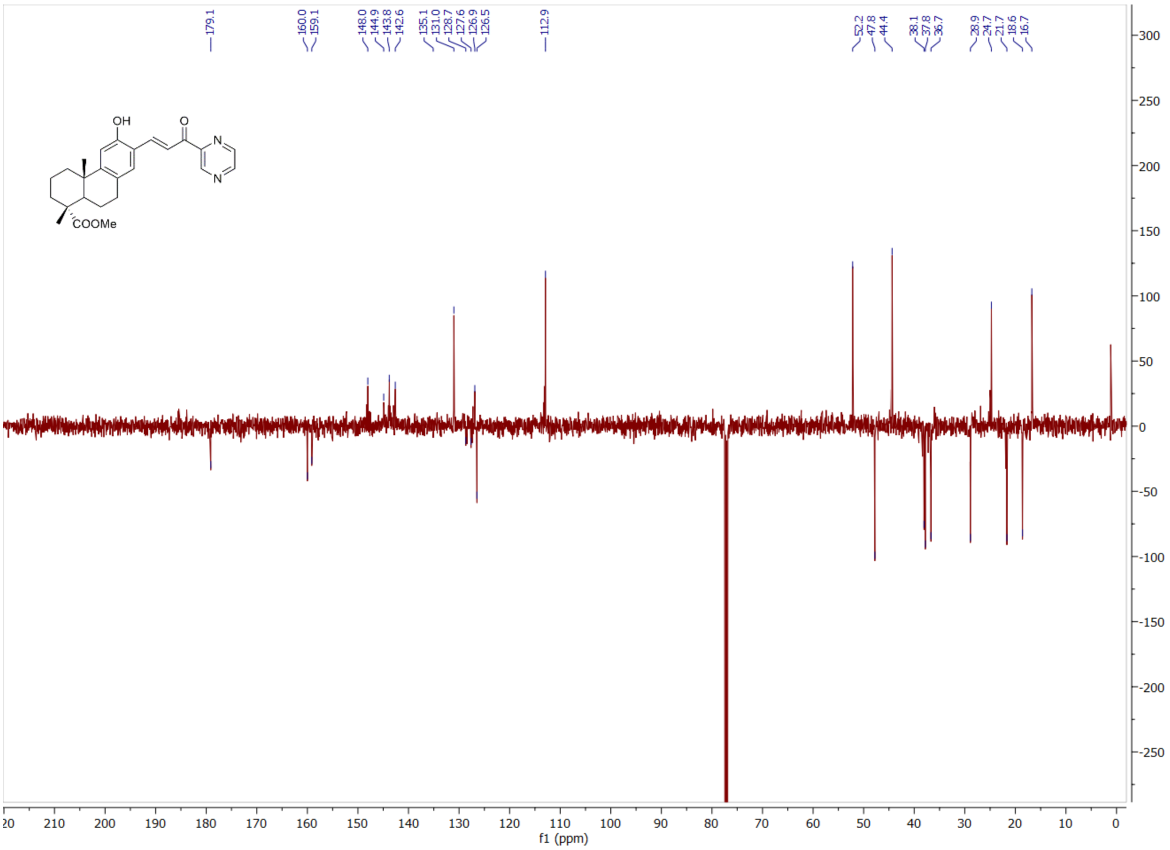

Figure S50. APT-NMR spectrum of compound 51.

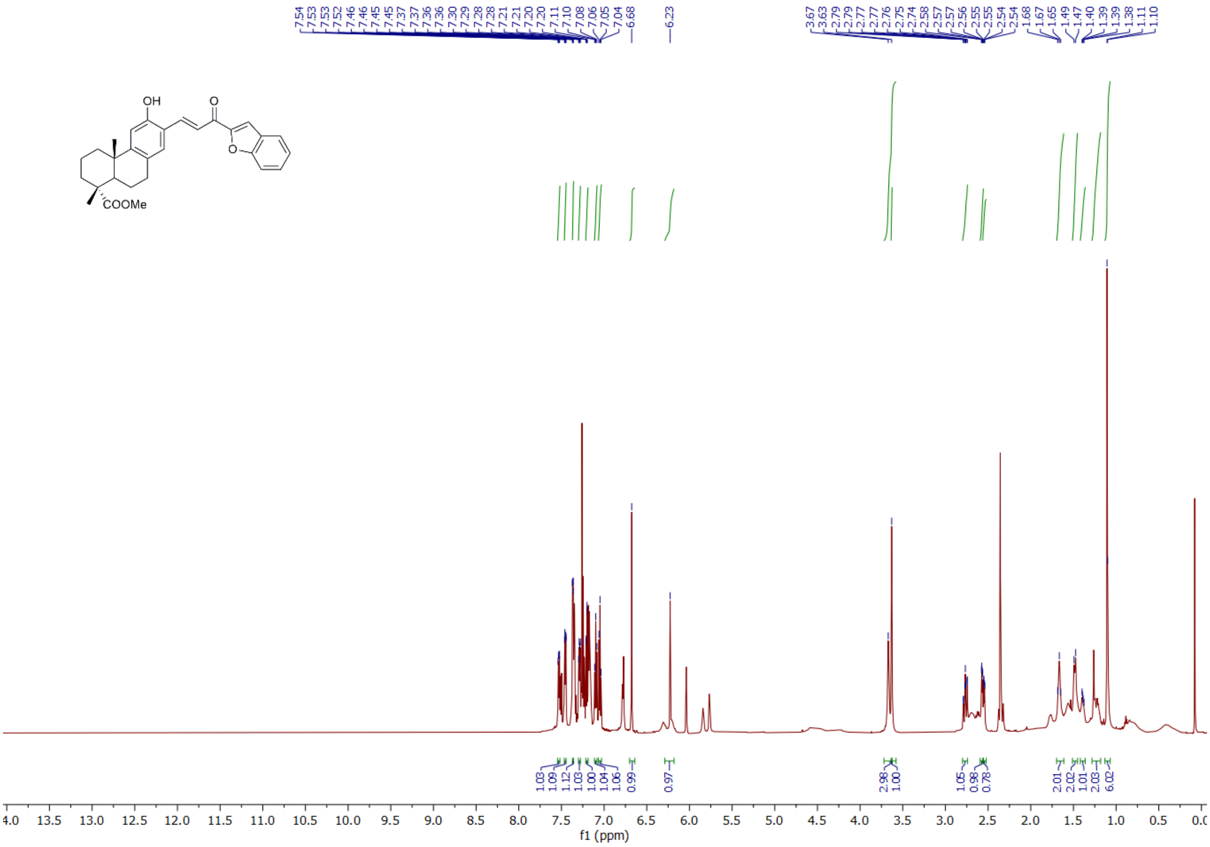

Figure S51 [1]**H-NMR spectrum of compound 52.**

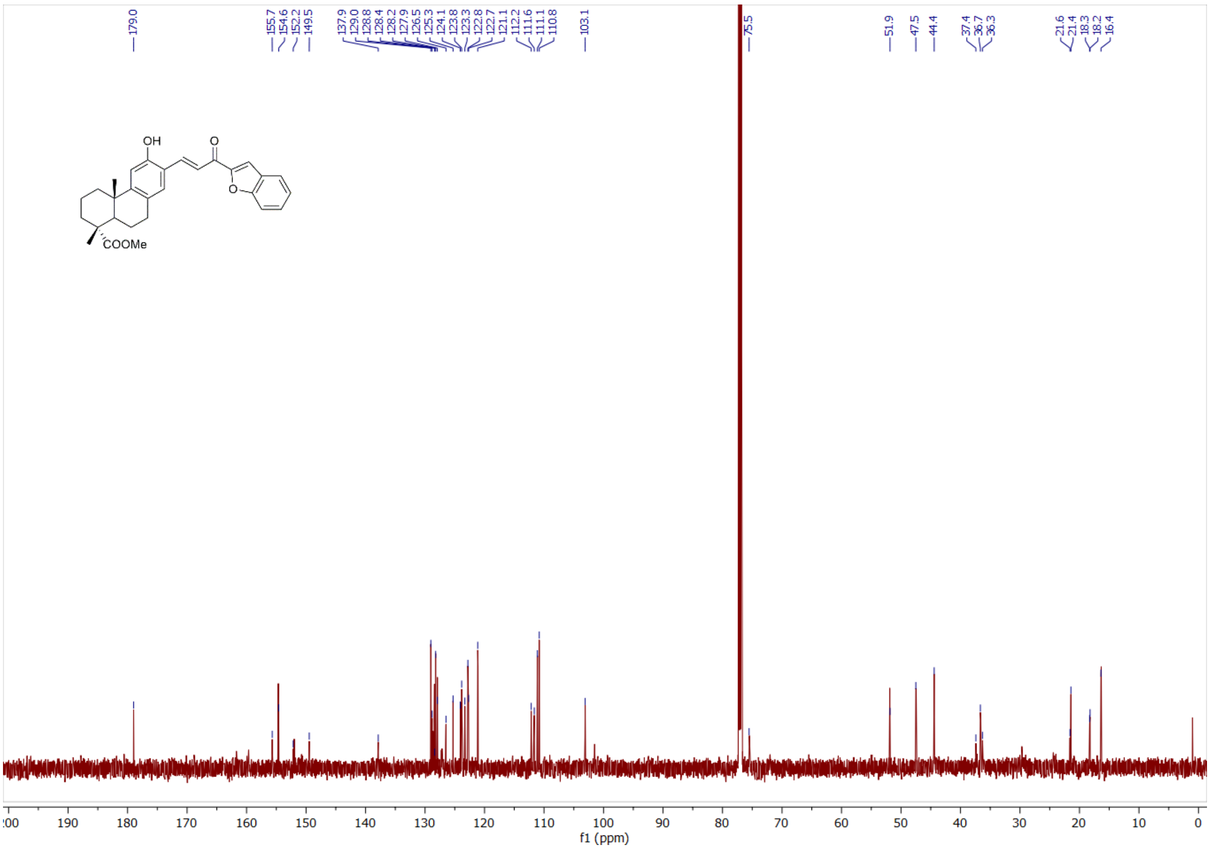

Figure S52. [13]**C-NMR spectrum of compound 52.**

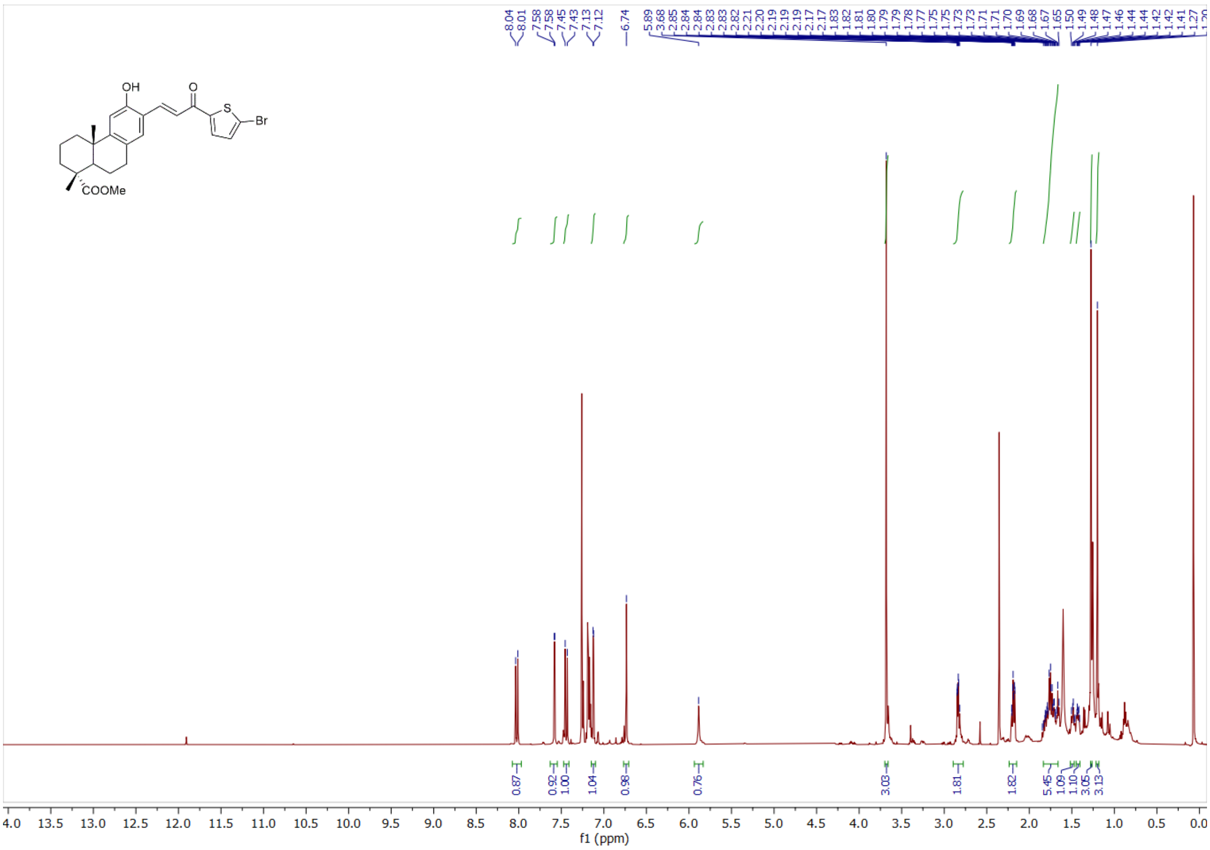

Figure S53. **^{1}H-NMR spectrum of compound 53.**

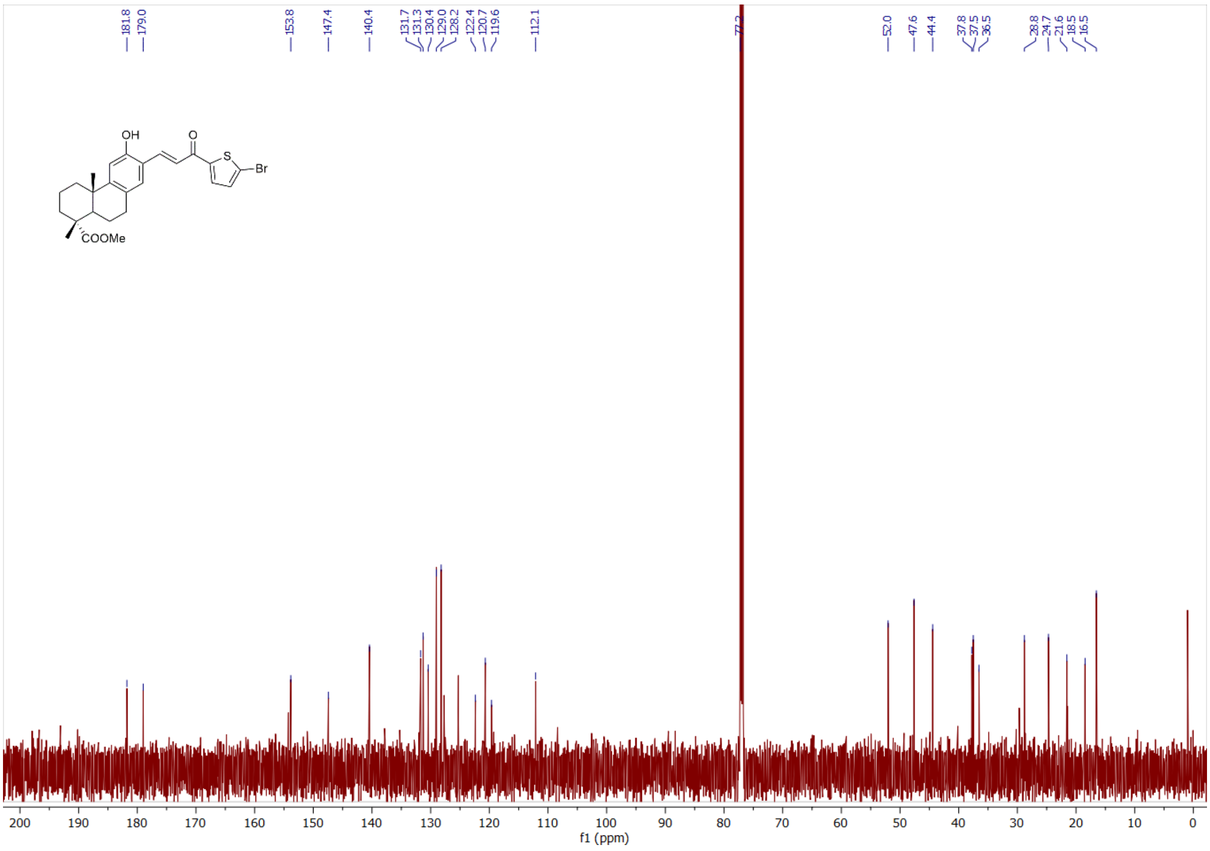

Figure S54. **^{13}C-NMR spectrum of compound 53.**
